# Systems pharmacology to reveal multi-scale mechanisms of traditional Chinese medicine for gastric cancer

**DOI:** 10.1038/s41598-021-01535-5

**Published:** 2021-11-12

**Authors:** Lulu Zhang, Yue Xiao, Ruijie Yang, Siyi Wang, ShuangXin Ma, Jianling Liu, Wei Xiao, Yonghua Wang

**Affiliations:** 1 Key Laboratory of Resource Biology and Biotechnology in Western China, Ministry of Education, School of Life Sciences, Northwest University, Xi’an, 710069 Shanxi China; 2 Lab of Systems Pharmacology, Center of Bioinformatics, College of Life Science, Northwest A&F University, Yangling, China; 3 State Key Laboratory of New-Tech for Chinese Medicine Pharmaceutical Process, Jiangsu Kanion Parmaceutical Co. Ltd., Lianyungang, 222002 China

**Keywords:** Cancer, Systems biology

## Abstract

Because of the complex etiology, the treatment of gastric cancer is a formidable challenge for contemporary medical. The current treatment method focuses on traditional surgical procedures, supplemented by other treatments. Among these other treatments, Traditional Chinese Medicine (TCM) plays an important role. Here, we used the systems pharmacology approach to reveal the potential molecular mechanism of PRGRC on gastric cancer which composes of *Pinellia ternata* *(Thunb.) Breit.*, *Rheum palmatum* *L.*, *Gentiana scabra* *Bunge*, *Radix Aucklandiae* and *Citrus aurantium L*. This approach combines pharmacokinetics analysis with pharmacodynamics evaluation for the active compounds screening, targets prediction and pathways assessing. Firstly, through pharmacokinetic evaluation and target prediction models, 83 potential compounds and 184 gastric cancer-related targets were screened out. Then, the results of network analysis suggested that the targets of PRGRC were mainly involved two aspects: apoptosis and inflammation. Finally, we verified the reliability of the above analysis at the cellular level by using naringenin and luteolin with good pharmacokinetic activity as representative compounds. Overall, we found that PRGRC could influence the development of gastric cancer from a multi-scale perspective. This study provided a new direction for analyzing the mechanism of TCM.

## Introduction

As one of the most common malignancies of the gastrointestinal tract, gastric cancer has a high incidence and poor prognosis^1^. Due to the atypical early symptoms, about 70% of patients with gastric cancer were in the late stage of diagnosis, which significantly limited the efficacy of surgery and radiotherapy. In the past decades, conventional chemotherapy drugs such as cisplatin (DDP) and 5-fluorouracil have brought great clinical benefits to patients with advanced gastric cancer. However, due to drug resistance and cytotoxicity, the clinical efficacy gradually deteriorates^2^. Traditional Chinese Medicine (TCM) is a natural medicine with low toxicity, high safety and efficacy and has curative effects on gastric cancer, affecting both basic pathophysiology and syndrome characteristics. And now, it has emerged as a research hotspot^3^.

Due to the synergistic pharmacological and pharmacokinetic effects of multiple active compounds, TCM formulas consisting of multiple herbs play a prominent role in the treatment of various diseases. Meanwhile, many herbs and formulas have been successfully used to treat gastric cancer. This article described one of these formulas—PRGRC which consists of five herbs: *Pinellia ternata* *(Thunb.) Breit.*, *Rheum palmatum* *L.*, *Gentiana scabra* *Bunge*, *Radix Aucklandiae* and *Citrus aurantium* *L*. Among these five herbs, it has been reported that *Pinellia ternata* *(Thunb.) Breit* can block SGC7901 cells in S phase, thereby inhibiting tumor activity by affecting cell cycle progression^4,5^. Then, previous researches have shown that *Rheum palmatum* *L.* and *Radix Aucklandiae* could inhibit cancer cell metastasis and induce apoptosis by regulating the expression of apoptosis-related proteins^6,7^. Reports in the literatures indicated that *Gentiana scabra* *Bunge* and *Citrus aurantium* *L.* have been commonly applied on diseases for their anti-inflammatory, anti-tumor and anti-oxidant properties^8,9^. For PRGRC, due to its complex composition, the molecular mechanism on gastric cancer was not yet clear. Therefore, a systematic approach to explain the mechanism of TCM is urgently needed.

Recently, the development of systems pharmacology provided a new way to explore TCMs across multiple scales of complexity ranging from molecular and cellular levels to tissue and organism levels^10^. In our previous studies, we have successfully constructed a new systems pharmacology approach that includes ADME characterization of drugs evaluation, target prediction and network analysis, which facilitated the identification of multiple mechanisms of drugs^11^. Through this platform, a variety of herbs including *Radix Salviae miltiorrhiza, Sophora flavescens*, *Sedum rosea* and *Glycyrrhiza uralensis* *Fisch*. have been verified to have potential mechanisms in the treatment of cancer, depression and cardiovascular diseases.

Therefore, in this article, we firstly analyzed the oral bioavailability (OB)^12^ and drug-likeness (DL)^13^ properties of compounds contained in PRGRC through systems pharmacology approach to obtain potential compounds. Next, based on the integrated target prediction methods, WES^14^ and SysDT^15^, which united the biological and mathematical models, we predicted the homologous targets of the screened potential compounds. Afterwards, the obtained targets were verified by functional enrichment analysis and the relevant networks were constructed. Ultimately, the network pharmacology and gastric cancer-related signaling pathways evaluation were carried out to systematically disclose the underlying reciprocity between active compounds, active targets and pathways. Based on the above, we explored the mechanism of PRGRC in the treatment of gastric cancer. In addition, naringenin and luteolin with good pharmacokinetic activities were used as representatives of the screened potential compounds for further verification in vitro. The results dissected the multi-scale mechanism of PRGRC, which provided examples for revealing the mechanism research of TCM.

## Results

### Active compounds screening

To reveal the multi-compound and multi-target features of PRGRC on gastric cancer, we collected a total of 442 compounds from TCMSP database of all five herbs in PRGRC. In order to screen for potential bioactive compounds of PRGRC, we evaluated the ADME (Absorption, Distribution, Metabolism, Excretion) properties of these compounds, including OB and DL. As shown in Table S1, 59 compounds reached the standard (OB ≥ 30%, DL ≥ 0.18). Besides, for more accurate results, some compounds that did not meet the criteria, but were reported to have anti-cancer activity in the literatures, were also added. Therefore, we screened a total of 83 potential compounds (accounting for 18.8% of all 442 compounds of PRGRC) (Supplementary Table S1).

### Drug targeting and analysis

In order to explore the targets of compounds for PRGRC in the treatment of gastric cancer, we identified a total of 365 direct and indirect targets associated with active compounds by means of WES and SysDT algorithms. The results showed that most compounds acted on multi-targets, proving various pharmacological effects of bioactive molecules. Afterwards, we mapped the 365 targets to the CTD database for disease enrichment analysis, thereby obtaining 184 targets associated with gastric cancer (Supplementary Table S2). Among the gastric cancer-related targets, B-Cell CLL/Lymphoma 2 (BCL2)^16^, Caspase 3 (CASP3)^17^, interleukin-10 (IL-10), nitric oxide synthase, inducible (NOS2), nuclear factor kappa-B (NFκB), Cyclooxygenase 2 (COX2)^18^ and other proteins were involved in the immunoregulation, apoptosis and inflammatory processes of gastrointestinal diseases, which may indicate the synergistic effect of compounds and targets on gastric cancer.

**Figure 1:**
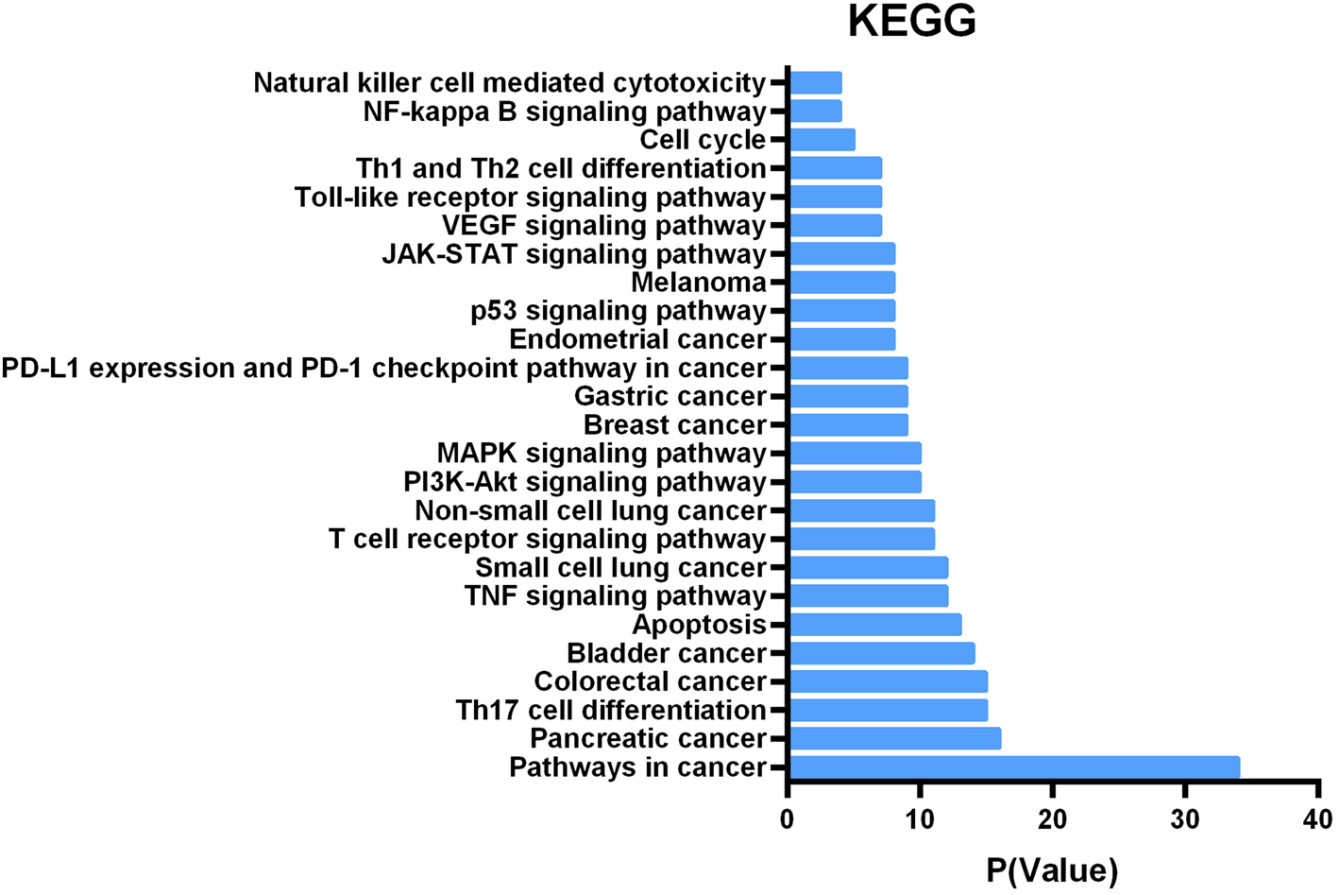
Kyoto Encyclopedia of Genes and Genomes (KEGG) analysis of potential targets. The Y-axis showed significantly enriched “pathway” categories in KEGG of the potential targets, and the X-axis showed the enrichment scores of these terms (P-value < 0.01).

We enriched the 184 targets in the KEGG database^19^, and the top 25 enriched KEGG terms were listed in Fig. 1, which indicated that most of these targets were closely related to various tumorigenic and immune-related signaling pathways, such as apoptosis, cell cycle, TNF signaling pathway, PI3K-AKT signaling pathway, Natural killer cell mediated cytotoxicity and so on. Besides, these targets were involved in multiple cancer pathways, including pancreatic cancer, colorectal cancer, bladder cancer, breast cancer, gastric cancer and so on. These suggested that PRGRC might exert the therapeutic effect on gastric cancer mainly by regulating immunity and apoptosis.

### Immune-related GO analysis

**Figure 2:**
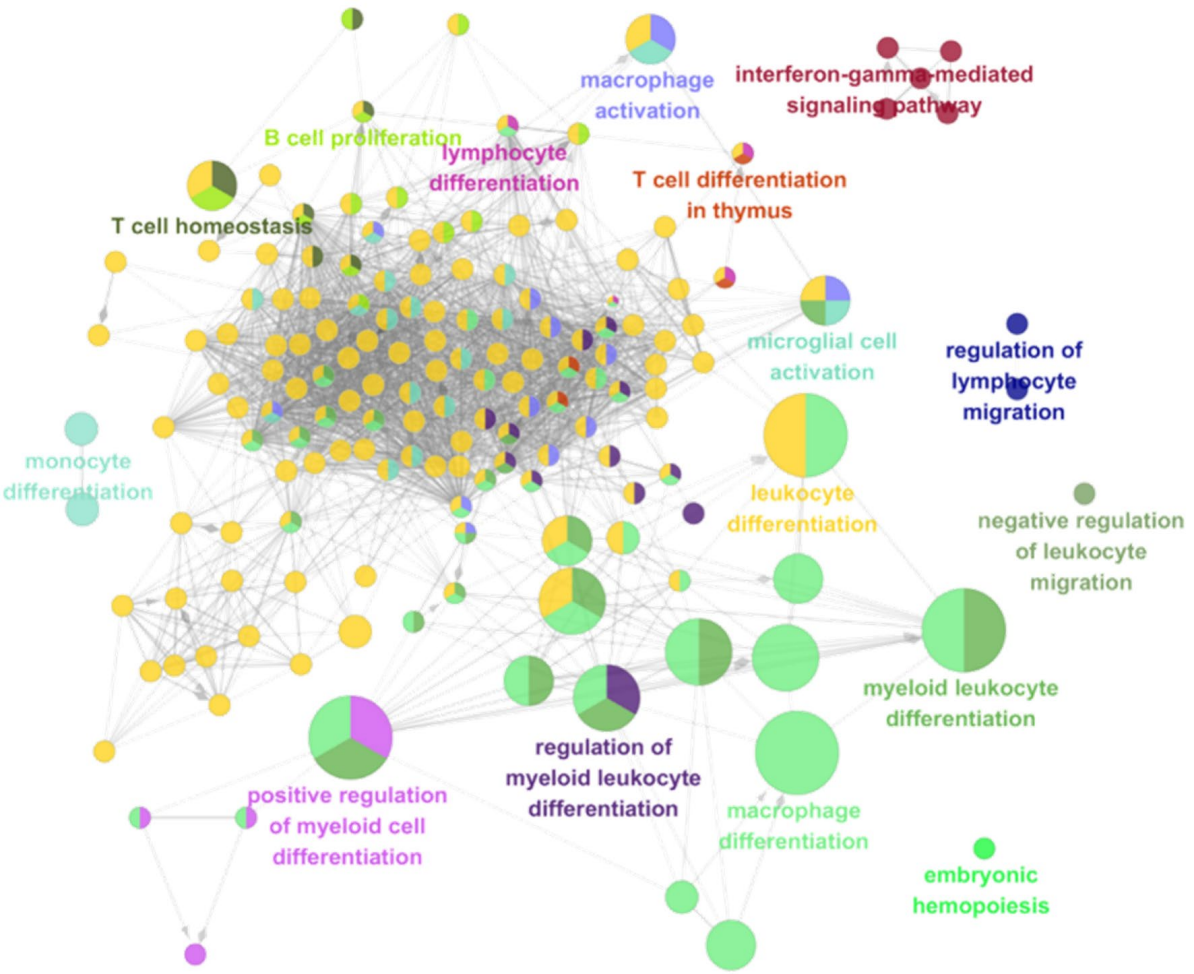
Immune-related GO analysis. The circles represented the targets involved in the immune-related process. The different colors represented the different corresponding immune-related biological processes. If a circle had more than one color, it meant that the target was involved in more than one immune-related process. And the size of the circle represented the degree of correlation between the target and the corresponding biological process. The larger the size of the circle, the greater the correlation between the target and the biological process. Gray lines represented interactions between compounds and targets.

We have enriched the possible pathways mediated by gastric cancer-related targets. To further illustrate the role of PRGRC on gastric cancer, we analyzed the biological process of 184 potential targets by cytoscape3.6.0. As shown in Fig. 2, we found that these targets were related to a variety of immune-related biological processes, such as: T cell differentiation, regulation of lymphocyte migration, leukocyte differentiation, B cell proliferation, T cell homeostasis, myeloid leukocyte differentiation, macrophage differentiation and so on. The results demonstrated that the potential immunomodulatory ability of PRGRC may be a part of its anti-cancer mechanism.

### Compound-target network and analysis

**Figure 3:**
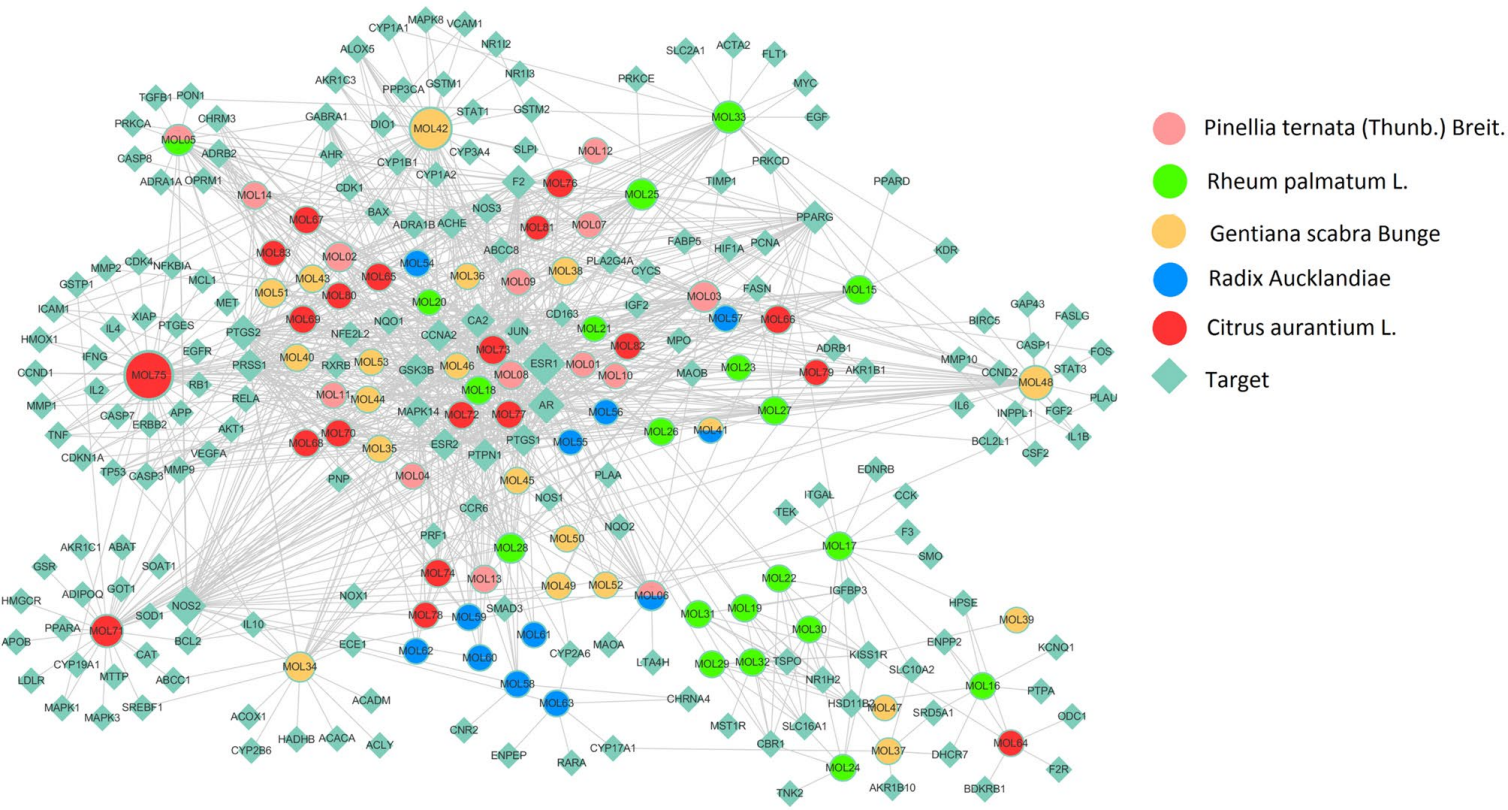
Compound-Target network. The olive green diamonds represented the potential targets. The other five colors represented the active molecules of five different herbs in PRGRC. If one circle had more than one color, it meant that this molecule exists in more than one herb. The larger the size of the circle, the greater the correlation between the target and the compound. Gray lines represented interactions between compounds and targets.

To directly reflect the compounds-targets relationship more, we constructed a compound-target (C-T) network by Cytoscape3.6.0. As shown in Fig. 3, the C-T network composed of 267 nodes and 962 sides. The 267 nodes consisted of 83 potential compounds and 184 targets. By analyzing the C-T network, we found that the average target degree of each compound is 12 which elucidated the multi-target properties of PRGRC. The targeting relationships of the compounds and targets were shown in Supplementary Table S3.

Among the 83 potential compounds, naringenin (MOL71) and luteolin (MOL75) were known to exert anti-inflammatory effects, as well as to inhibit the proliferation of various cancer cell lines. Naringenin (MOL71) with a degree of 36 and good pharmacokinetic and pharmacodynamic properties have been reported to exert anti-inflammatory effects as an inhibitor of PTGS2 and NOS2^20^. Meanwhile, the researches indicated that luteolin (MOL75) with the highest degree could suppress inflammation-associated gene expression by blocking the activation pathway of RELA and AP-1^21^. Gentiopicroside (MOL50)^22^ and Rhein (MOL18)^23^ have been proved to inhibit tumor development through regulating PI3K-Akt signaling pathway. And the researches indicated that beta-sitosterol (MOL05)^24^ acted as the activator of estrogen receptor (ESR1) and transcription factor AP-1 (JUN). By referring the relevant literatures, we found that kaempferol (MOL42) had the effect on regulating adaptive humoral immunity by managing primary B cells and T cells in vivo^25^ and it may reduce the intensity of inflammatory processes by inhibiting the secretion of proinflammatory cytokine TNF-α, additionally by increasing the secretion of anti-inflammatory cytokine IL-10^26^. Thus, these potential compounds of PRGRC acted by modulating inflammatory responses.

Of the 184 potential target nodes, many potential targets were linked with multiple compounds of different herbs, which may exhibit synergism effects or additive effects of PRGRC on gastric cancer. For example, NOS2 is associated with regulation of immune effector process^27^. COX2 is the inducible form of cyclooxygenase and is responsible for the rate-limiting step in the conversion of arachidonic acid to prostanoids (prostacyclins, prostaglandins, and thromboxanes). Prostanoids have been well-characterized as proinflammatory mediators^28^. Both CASP3 and BCL2 were apoptosis-related proteins, which inhibited tumor growth by regulating and inducing cancer cell apoptosis^29^. C-T network analysis showed that naringenin and luteolin could target these above-mentioned targets.

Therefore, these compounds and targets might be closely related to gastric cancer, which demonstrated the potential therapeutic effect of PRGRC on gastric cancer through modulating the reaction of multiple compounds and multiple targets.

### Target-pathway network and analysis

**Figure 4:**
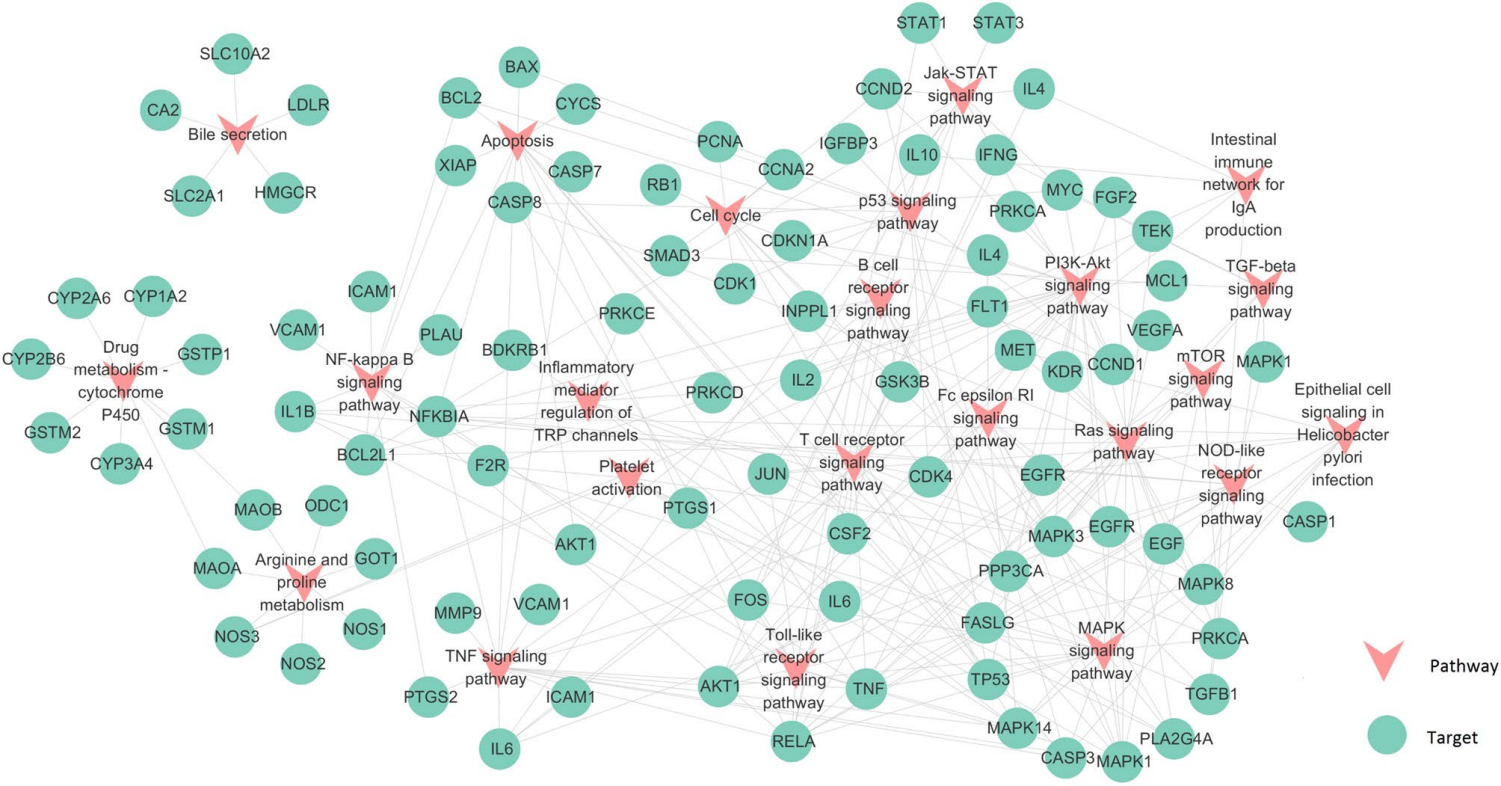
Target-Pathway network. The pink nodes represented pathways extracted by KEGG database which might be related to gastric cancer, the olive green circles represented targets. Gray lines represented interactions between pathways and targets.

Through integrating the KEGG and immune-related GO terms, we discovered 23 dramatically enriched pathways (P-value < 0.01) that might be the main pathways and play a key role in gastric cancer. As shown in Fig. 4, we mapped a T-P network which was mapped from 116 nodes and 268 sides and the average degree of per pathway is 11.7. The 116 nodes included 23 pathways and 93 targets (Supplementary Table S4). Meanwhile, the network illustrated that most of the potential targets were linked to more than one pathways. PI3K-Akt signaling pathway (degree = 29), NF-κB signaling pathway (degree = 11), TNF signaling pathway (degree = 17), Apoptosis (degree = 14), Toll-like receptor signaling pathway (degree = 14) and T cell reporter signaling pathway (degree = 17) might be the key pathways for PRGRC to produce anti-tumor effects. Actually, these pathways have been identified to regulate the expression of many genes, including those involved in responses ranging from inflammation and immunity to cell growth, proliferation and apoptosis^30,31^.

In summary, PRGRC played a role in the treatment of gastric cancer by acting on targets involved in cancer-related pathways.

### Pathway analysis

**Figure 5:**
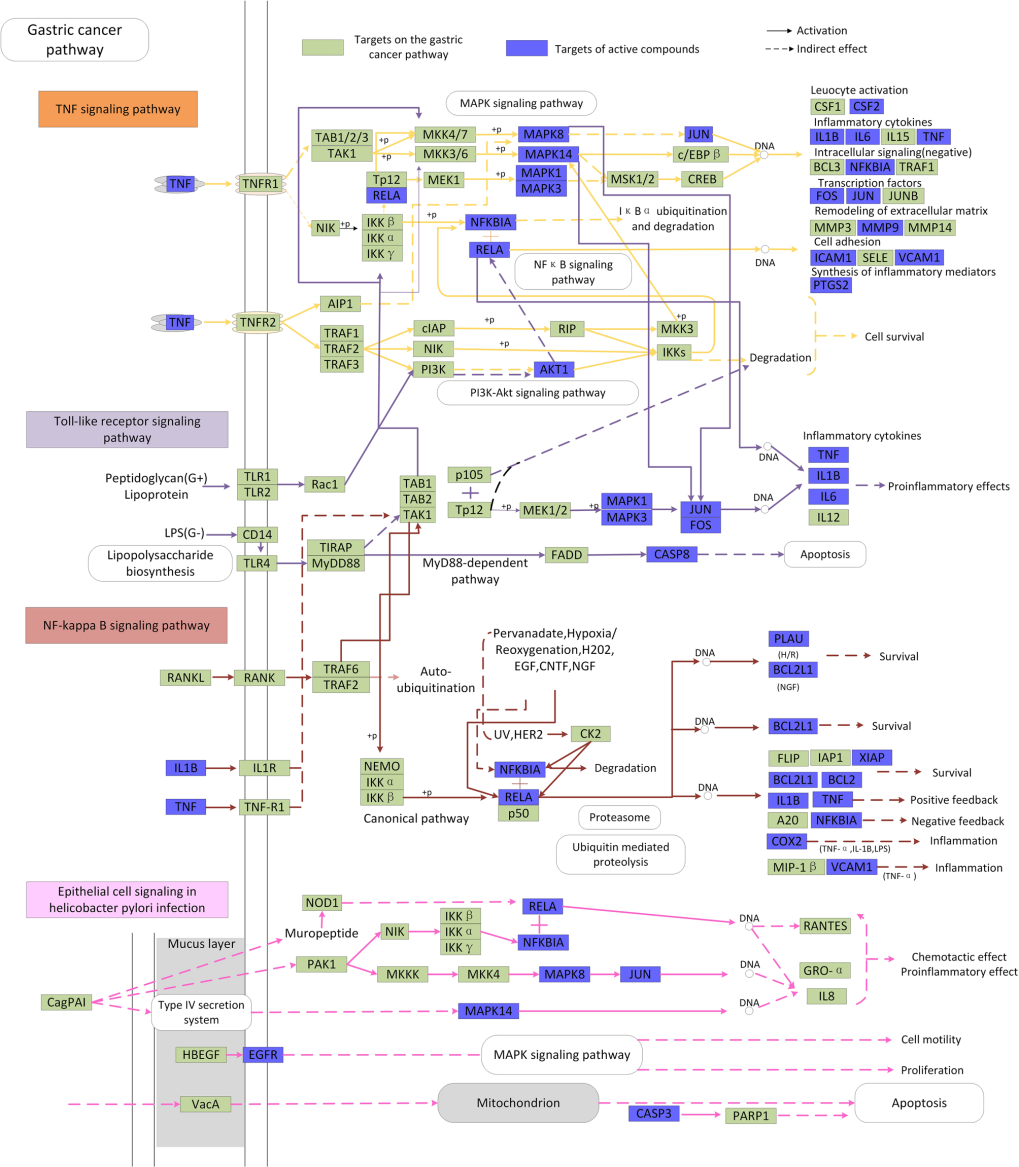
The gastric cancer pathway and therapeutic modules. The blue squares represented efficacy targets, the green squares represented the other targets in the gastric cancer pathway. Solid arrows indicated activation and dashed arrows indicated inhibition. Different pathways were represented by different colors.

All of the disorders have common features or conjoint cellular processes, like the activation of a stress signaling pathway and the concomitant production of inflammatory cytokines. Hence, to further elucidate the mechanism of PRGRC, we collected the targets and pathways closely related to gastric cancer. Then, we assembled the “gastric cancer pathway” according to the pathological and clinical data. As shown in Fig. 5, the gastric cancer pathway was separated into two representative therapeutic modules (Immunoregulation module and Apoptosis module).

#### Immunoregulation module

Humoral and cell-mediated immune mechanisms were likely to be involved in the treatment of gastric cancer. As shown in Fig. 5, many targets were associated with immune and inflammatory responses. For instance, NF-κB (RELA) has been shown to be one of the most important regulators of pro-inflammatory gene expression, mediating the expression of inflammatory cytokines such as IL-2, IL-4, IL-10^32^ and TNF-α^33^, and the activation of NF-κB caused inflammation of the gastrointestinal mucosa, leading to the occurrence of gastric cancer^34^. IL-2 was proved to be a T-cell growth factor that enhanced T cells and NK cell-mediated immune responses^35^. Besides, The literatures revealed that IL-10 acted as a multifunctional negative regulator participating in the bioregulation of immune cells, inflammatory cells and other cells^36^. As shown in Fig. 3 and Supplementary Table S3, we obtained that some compounds including naringenin (MOL71), luteolin (MOL75), kaempferol (MOL42), ursolic acid (MOL48), baicalein (MOL03) and isovitexin (MOL35) were all targeted at NF-κB which probably played a vital role in gastric cancer. Macrophages responded to bacterial infections or other immune responses and secreted TNF, which can synergize with interferon to kill *Helicobacter pylori*^37^. All these indicated that PRGRC might treat gastric cancer through the regulation of immune and inflammatory responses.

#### Apoptosis process

Apoptosis is a process of programmed cell death that occurs in multicellular organisms. Under normal circumstances, homeostasis is maintained by the balance between cell proliferation and apoptosis^38^. Abnormal apoptosis and excessive proliferation of gastric epithelial cells induced to the occurrence of gastric mucosal lesions, which in turn caused gastric cancer^39^. The literatures indicated that gastric cancer was closely related to *Helicobacter pylori* infection which increased the apoptosis of gastric epithelial cells^40^. Cytotoxin-associated gene A (CagA) and Vacuolating cytotoxin gene A (VacA) were the main pathogenic factors of *Helicobacter pylori*^39^, which could induce the production of various cytokines in the gastric mucosa, leading to apoptosis of mucosal epithelial cells^41,42^. It has been reported that aloe-emodin (MOL25) and emodin (MOL33) could inhibit the growth of *Helicobacter pylori* and reduce the apoptosis of gastric mucosa cells caused by CagA and VacA, thereby protecting the gastric mucosa and achieving the purpose of treating gastric cancer^43^.

Previous studies have confirmed that bcl-2 family played an important role in the apoptosis of gastric mucosa. When gastric cancer occurred, BCL2 inhibited the abnormal apoptosis of gastric mucosal cells by targeting pro-apoptotic proteins to the mitochondrial membrane, thereby preventing the development of gastric cancer^44,45^. Caspase-3, a key pro-apoptotic factor, could be activated as cleaved-CASP3 to regulate the abnormal apoptosis of gastric mucosal epithelial cells^46^. Studies have found that BCL2 could not only act as an upstream factor of CASP3, but also as a direct substrate of CASP3 in the downstream, both of which were interrelated and mutually restricted in the process of apoptosis transmission^47,48^.

PARP (poly (ADP-ribose) polymerase) family proteins were essential in the repair of single-stranded DNA breaks via the base excision repair pathway. PARP1 was a highly conserved DNA-binding protein and was the most ubiquitously expressed member of the PARP family. Numerous studies suggested that PARP1 was involved in maintaining genomic stability as well as regulating DNA repair and transcriptional processes. PARP1 was now considered to be a central regulatory hub of cell survival and cell death as well as a key component of a number of transcription factors involved in tumor development and inflammation. CASP3 could cleave PARP1 with full length of 116 kDa into two fragments of 31 kDa and 85 kDa, resulting in PARP1 not functioning properly and causing DNA breakage. The degradation of PARP1 was one of the hallmarks of apoptosis^49^.

Naringenin (MOL71) and luteolin (MOL75) regulated the expression of BCL2 and CASP3 and indirectly regulated the expression of PAPR1 to inhibit the development of gastric cancer. Thus, all above suggested that the active ingredients in PRGRC were able to activate various cytokines and pathways and might treat gastric cancer by regulating immunity and apoptosis.

### Experimental validation

#### Cell viability analysis treated with compounds

From the pathways analysis, the synergistic effects of active compounds on gastric cancer were identified. Naringenin (MOL71) (OB = 59.29%, DL = 0.21, degree = 36) and luteolin (MOL75) (OB = 36.16%, DL = 0.25, degree = 49), screened via systems pharmacology approach, have been identified to exhibit good pharmacodynamic properties. Thus, we selected naringenin and luteolin as representative compounds to design the in vitro experiments to investigate the efficacy on cancer cells and inflammatory cells.

**Figure 6:**
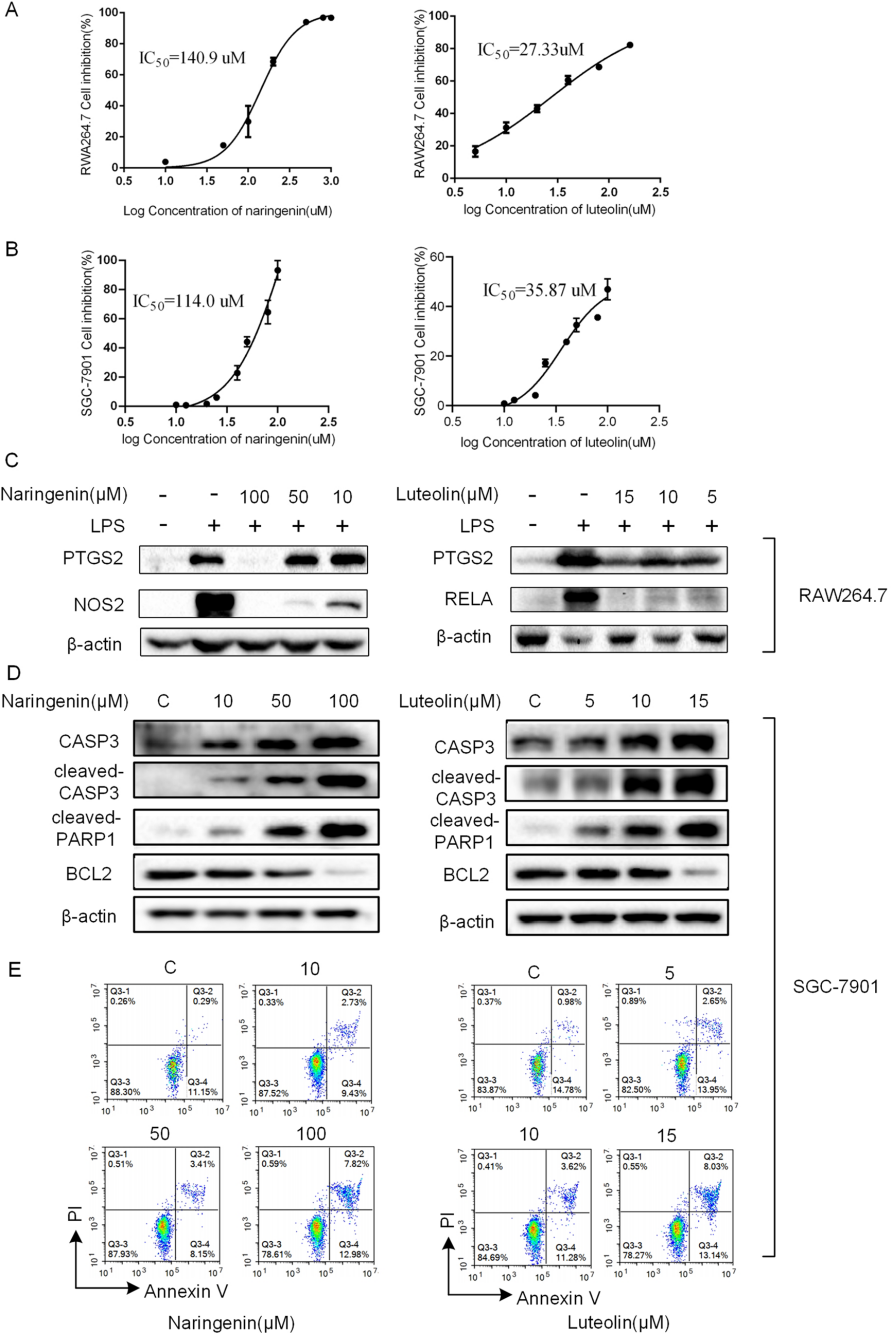
In vitro verification of potential effects of compounds associated with anti-inflammatory and apoptosis. (**A,B**). CCK-8 assay of the inhibition of RAW264.7 cells (**A**) and SGC-7901 cells (**B**) treated with naringenin (Left) and luteolin (Right), respectively. The X-axis showed the drug concentration. The Y-axis showed the cell inhibition. (**C**) RAW264.7 cells were pre-treated with naringenin (Left) and luteolin (Right) for 2 h, then exposured to LPS (1 μg/ml) for 18 h. Then NOS2, RELA and PTGS2 were measured by western blot. β-actin was used as a loading control. The blots were cut prior to hybridization with antibodies. The full-length blots/gels were presented in Supplementary Figs. S2–S7. (**D**) Western blot showed the expression of apoptotic proteins (CASP3, cleaved-CASP3 and cleaved-PARP1, BCL2) in SGC-7901 cells. The blots were cut prior to hybridization with antibodies. The full-length blots/gels were presented in Supplementary Figs. S8–S16. (**E**) Apoptosis in SGC-7901 cells was assessed after 24 h of treatment with naringenin (Left) and luteolin (Right) by Annexin V-FITC/PI binding and measured by flow cytometry analysis.

CCK-8 assay showed that the IC_50_ of RAW264.7 cells treated with naringenin and luteolin were 140.9 μM and 27.33 μM, respectively (Fig. 6A). Then, the IC_50_ of SGC-7901 cells treated with naringenin and luteolin were 114 μM and 35.87 μM, respectively (Fig. 6B). As shown in Supplementary Fig. S1A, the IC_50_ of AGS cells treated with naringenin and luteolin were 140.6 μM and 34.38 μM, respectively. These results indicated that naringenin and luteolin have a significant inhibitory effect on inflammatory cells and gastric cancer cells. As the lower doses of naringenin and luteolin did not inhibit the viability of above cells, the concentrations of naringenin (10, 50 and 100 μM) and luteolin (5, 10 and 15 μM) were selected for the follow-up experiments.

#### In vitro verification of potential effects of compounds associated with anti-inflammatory and apoptosis

To further assess the potential anti-inflammatory effects of naringenin and luteolin, we used RAW264.7 cells treated by LPS with or without naringenin and luteolin in vitro. Thereafter, we verified the protein expression levels of PTGS2, NOS2 or RELA by western blot to confirm whether these two compounds have anti-inflammatory effects. The results in Fig. 6C illustrated that the protein expression levels of PTGS2, NOS2 and RELA in the RAW264.7 cells treated with LPS were significantly higher than those in control (Fig. 6C). After 18 h treatments of naringenin and luteolin, we found production of the three proteins was diminished standing in sharp contrast to the group of LPS, as expected (Fig. 6C). These results suggested that PRGRC exerted the therapeutic effect on gastric cancer by modulating the immune response.

Meanwhile, we performed western blot on BCL2, CASP3, cleaved-CASP3 and cleaved-PARP1 proteins in SGC-7901 and AGS cells to evaluate the anti-apoptotic effect of naringenin and luteolin. The results for SGC-7901 cells were shown in Fig. 6D and the results for AGS cells were shown in Supplementary Fig. S1B. WB analysis of the two cell lines both displayed that the expression of BCL2 protein decreased with the treatment of naringenin and luteolin. However, given the distinct properties of these two proteins, the protein expression of CASP3 and cleaved-CASP3 appeared to be the opposite of BCL2, which was consistent with our expected results. Meanwhile, as the expression of CASP3 increased in the administered group, PARP1 was cleaved resulting in elevated expression of cleaved-PARP1 (85 kDa). Furthermore, by flow cytometry analysis, compared with control group, naringenin and luteolin dramatically triggered apoptosis in human SGC-7901 cells (Fig. 6E) and AGS cells (Supplementary Fig. S1C). These results revealed that PRGRC could induce apoptosis of gastric cancer cells.

Taken together, in vitro experiments indicated the molecular mechanisms of PRGRC on gastric cancer including regulation of immunity and promotion of apoptosis, which further validated the results obtained through systems pharmacology and ensured the reliability of in silico screen strategy.

## Discussion and conclusion

Gastric cancer has become one of the most common cancers due to high mortality and morbidity. Clinically, adverse drug reaction, drug resistance and cancer recurrence frequently occur, causing poor prognosis in gastric cancer patients. Therefore, the search for safe drugs with low side effects is necessary and urgent.

In this study, we selected PRGRC commonly used in clinical practice as an example, and proposed the systems pharmacology approach to interpret the synergistic pharmacological mechanism of the TCM formula in the treatment of gastric cancer. Firstly, based on the evaluation methods, 83 active ingredients were obtained, and 184 potential disease-related targets were predicted. The results indicated that PRGRC has the features of multiple compounds and multiple targets. Both active compounds and C-T analysis then attested that several active compounds in PRGRC were imperative for the treatment of gastric cancer, including naringenin and luteolin. In addition, some targets such as PTGS2, NOS2, CASP3, and BCL2 have been confirmed to have anti-inflammatory and pro-apoptotic effects on gastric cancer cells. Moreover, the results of pathway and GOBP enrichment analysis, T-P analysis and integrated the "gastric cancer pathway" suggested that PRGRC primarily treated gastric cancer by modulating inflammatory response and apoptosis. Finally, in vitro cytotoxicity test showed that naringenin and luteolin had obvious antiproliferative effect on gastric cancer cells. Besides, we verified that naringenin and luteolin has significant anti-inflammatory effect and regulate apoptosis of gastric cancer cells by western blot and flow cytometry.

In summary, the systems pharmacology approach revealed the complex molecular mechanisms of PRGRC for gastric cancer and elucidated the multi-molecular and multi-target synergistic effects of TCM. Moreover, this strategy provided a potential approach for rational discovery of new drugs. In the development of new drugs, we can try to determine the pharmacological activity of compounds by predicting their relevant targets and diseases. This approach accelerated the process of new drug development and promoted the modernization of TCM.

## Materials and methods

### Molecular database building

The compounds contained in PRGRC were collected from the Traditional Chinese Medicine Systems Pharmacology Database which has been developed by our previous studies^50^ (TCMSP, http://lsp.nwu.edu.cn/).

### Screening active compounds

PRGRC is an oral formulation and must undergo the ADME process to reach the target organs and tissues to exert their efficacy. In this article, we employed OB and DL properties as parameters for screening bioactive compounds^51^. And the thresholds for OB and DL were respectively set to 30% and 0.18.

### Drug targets

Targets are usually bioactive macromolecules in the body that recognize and bind pharmacodynamic molecules for the purpose of treating disease. Hence, target prediction plays an important role in elucidating the molecular mechanisms and drug actions associated with gastric cancer. In this work, we predicted the direct and indirect efficacy targets of PRGRC through the WES and SysDT algorithms, respectively.

The WES algorithm used chemical molecular fingerprints and Dragon parameters to calculate the weighted Tanimoto similarity between compounds and protein ligands, and then described the Global Similarity of compounds and protein ligands by pooled similarity. As a novel tool, the obtained model performed well in predicting the binding with average sensitivity of 85% (SEN) and the non-binding patterns with 71% (SPE) with the average areas under the receiver operating curves (ROC, AUC) of 85.2% and an average concordance of 77.5%^14^.

SysDT is an *in-house* computer model based on two powerful mathematical tools of Random Forest (RF) and Support Vector Machine (SVM) to model drugs and targets and their interactions by integrating information from large-scale chemical, genomic and drug data. The model had an overall accuracy of 97.3%, 87.7% accuracy in activation prediction and 99.8% accuracy in inhibition prediction. To capture more potential components, this study defined the filtering criteria as RF value ≥ 0.7 or SVM ≥ 0.8^15^.

We mapped the targets predicted above to the CTD database (CTD, http://ctdbase.org/) for disease enrichment analysis to obtain the potential targets associated with gastric cancer.

### GO enrichment and analysis of targets

In order to further probe the involved pathways and the biological processes of the targets obtained above, we mapped the targets to the KEGG (www.kegg.jp/kegg/kegg1.html)^52,53^ and DAVID (http://david.abcc.ncifcrf.gov) database^54^. Only P-value < 0.01 were selected.

### Network and pathway construction and analysis

In this study, for the sake of verifying the molecular mechanisms of PRGRC in the treatment of gastric cancer, we constructed the C-T network and T-P network by Cytoscape3.6.0^55^. In networks, the compounds, targets and pathways were represented by nodes, and the interaction between nodes were represented by edges. In addition, degree, a key topology parameter for evaluating nodes in the network, was analyzed by plugin Network Aalyzer of Cytoscape3.6.0. The degree of a node was the number of edges associated with the node. In order to further prove the integrative mechanisms of PRGRC, the key pathways obtained from the analysis of T-P network and C-T network were assembled into a “gastric cancer pathway” based on the current pathological information of gastric cancer.

### Experimental validation

#### Sample preparation

Naringenin and luteolin were purchased from Shanghai Yuanye Biological Technology Co., Ltd. (Shanghai, China). The samples were dissolved in DMSO to obtain a stock solution at a concentration of 100 mM and then stored at 4 °C. The dilution volume of DMSO finally added to the medium did not exceed 1% of the total volume, to ensure that DMSO has no effect on cell viability.

#### Cell culture

Mouse macrophage cells RAW264.7, human gastric cancer cells SGC7901 and AGS were purchased from Chinese Academy of Sciences Shanghai cell bank. RAW264.7 cells were cultured in Dulbecco’s modified eagle’s medium (DMEM) with 10% foetal bovine serum (FBS) (Gibco BRL). SGC7901 and AGS cells were cultured in RPMI-1640 medium with 10% FBS. All experiments cells were maintained in a humidified atmosphere of 5% CO_2_ at 37 °C.

#### Cell viability assay

Cell viability was estimated by CCK-8 assay (BestBio). RAW264.7, SGC-7901 and AGS cells were seeded into 96-well plate at a density of 8 × 10^4^ cells/ml for 24 h, and then cells were treated with or without various indicated concentrations of naringenin or luteolin, and then cells viability were measured by CCK-8 after 24 h. The various indicated concentrations of naringenin or luteolin were respectively 10, 50 and 100 μM or 5, 10 and 15 μM, respectively.

#### Inflammation model

RAW264.7 cells (1.5 × 10^6^) were cultured in 100 mm diameter dishes for 24 h and then treated with or without various indicated concentrations of naringenin or luteolin for 2 h before treated with 1 μg/ml LPS (Sigma, *E. coli* (O111:B4)), after that, cells were cultured in a humidified incubator with 5% CO_2_/95% O_2_ at 37 °C for 18 h. The cells were collected at the end of the culture for western blot, which were used as a detection of inflammatory mediators.

#### Western blot

Protein samples were separated by SDS-PAGE on 10% polyacrylamide gel and transferred to PVDF membranes. Subsequently, the membranes were blocked in 3% bovine serum albumin at room temperature and placed in primary antibody, NOS2, PTGS2, BCL2, RELA, CASP3, cleaved-CASP3 and cleaved-PARP1 (Abcam) overnight. Blots were then incubated in secondary antibody for 1 h before being developed with ECL chemiluminescence detection kit (Bio-Rad Laboratories). The images of the original blots were obtained by Image Lab software.

#### Flow cytometry analysis

Cell apoptosis was determined using a FITC Annexin V apoptosis kit (BD Bioscience) according to the manufacturer’s instructions. Following treatment with various concentrations of naringenin and luteolin for 24 h, the cell suspension was prepared using trypsin and centrifuged at 1000 rpm for 3 min then rinsed with ice-cold PBS. Cells were then resuspended in binding buffer at a concentration of 1 × 10^5^ cells/ml. Cells were stained with annexin V-FITC for 15 min and propidium (PI) for 5 min in the dark before analysis by a flow cytometer (NovoCyte 3130, ACEA).

### Statistical analysis

Statistical analysis were performed GraphPad Prism 8.0 (Graph Pad Software Inc). All data were presented as means ± standard error and repeated at least two or three independent experiments with the same result.

## Supplementary Information

Supplementary Figures.

Supplementary Tables.

## Data Availability

The datasets used and/or analyzed during the current study were available from the corresponding author on reasonable requests.
