# Supplementary material for: Systems pharmacology to reveal multi-scale mechanisms of traditional Chinese medicine for gastric cancer
Source: Sci Rep. 2021 Nov 12;11:22149. doi: 10.1038/s41598-021-01535-5 (PMC8589993; doi:10.1038/s41598-021-01535-5)

**Supplementary Figures**

Supplementary Figure S1. *In vitro* verification of potential effects of compounds associated with apoptosis in AGS cell line. (A). CCK-8 assay of the inhibition of AGS cells treated with naringenin (Left) and luteolin (Right), respectively. The X-axis showed the drug concentration. The Y-axis showed the cell inhibition. (B) Western blot showed the expression of apoptotic proteins (CASP3, cleaved-CASP3 and cleaved-PARP1, BCL2) in AGS cells. The blots were cut prior to hybridization with antibodies. The full-length blots/gels were presented in Supplementary Figure S17-S25. (C) Apoptosis in AGS cells was assessed after 24h of treatment with naringenin (Left) and luteolin (Right) by Annexin V-FITC/PI binding and measured by ﬂow cytometry analysis.


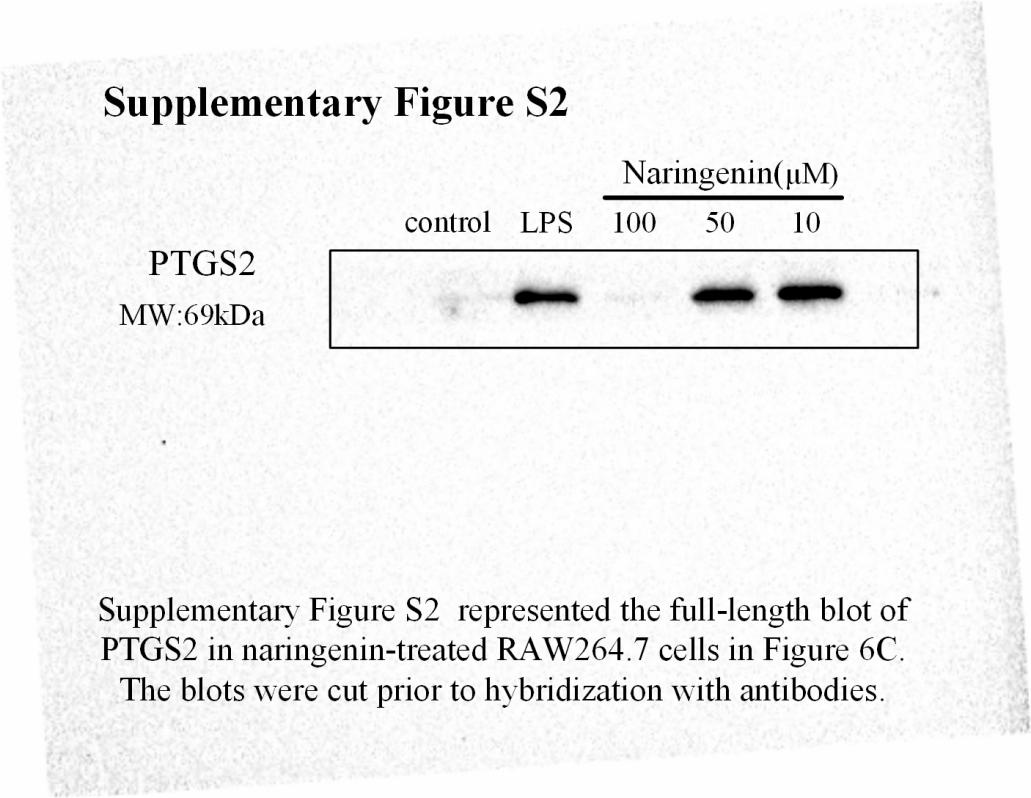


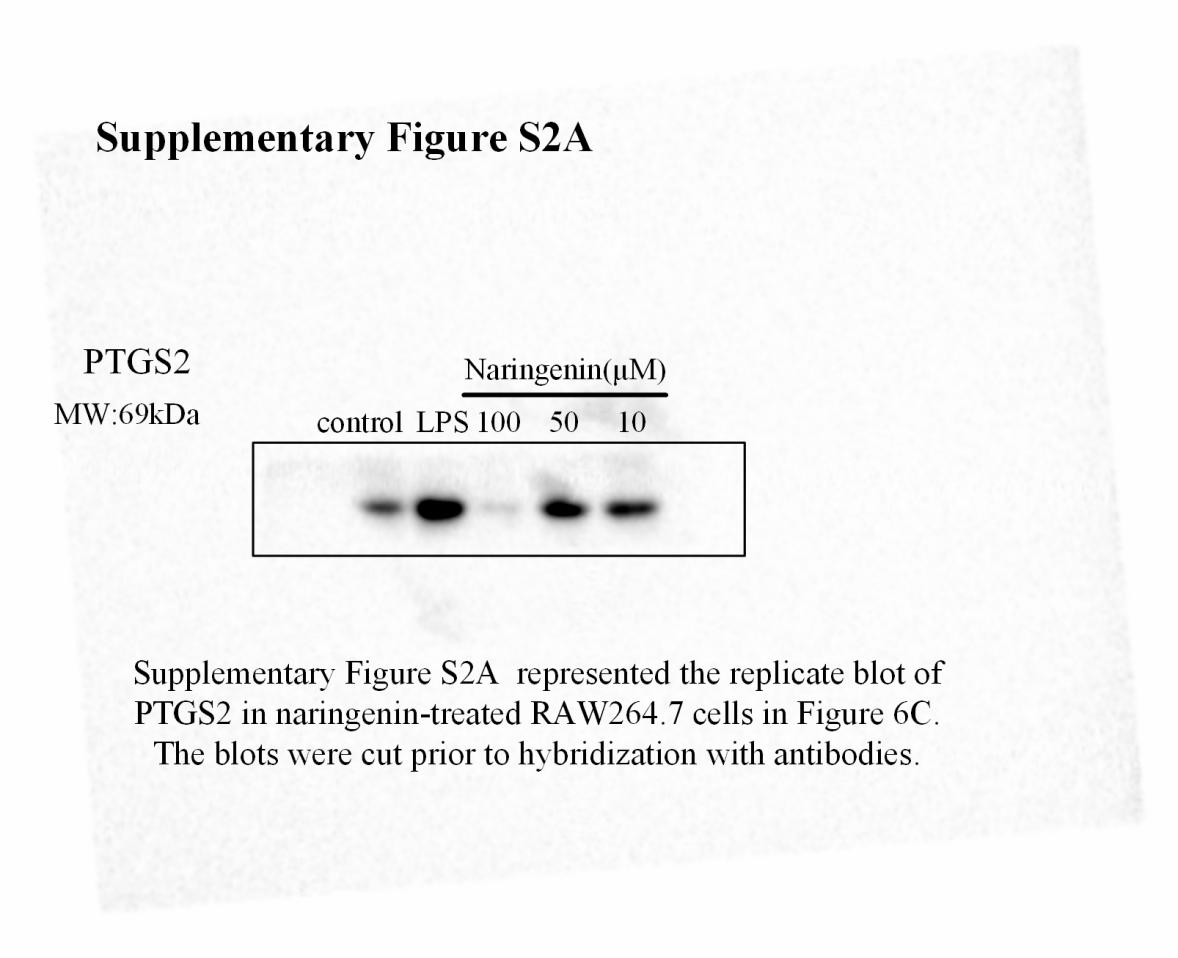


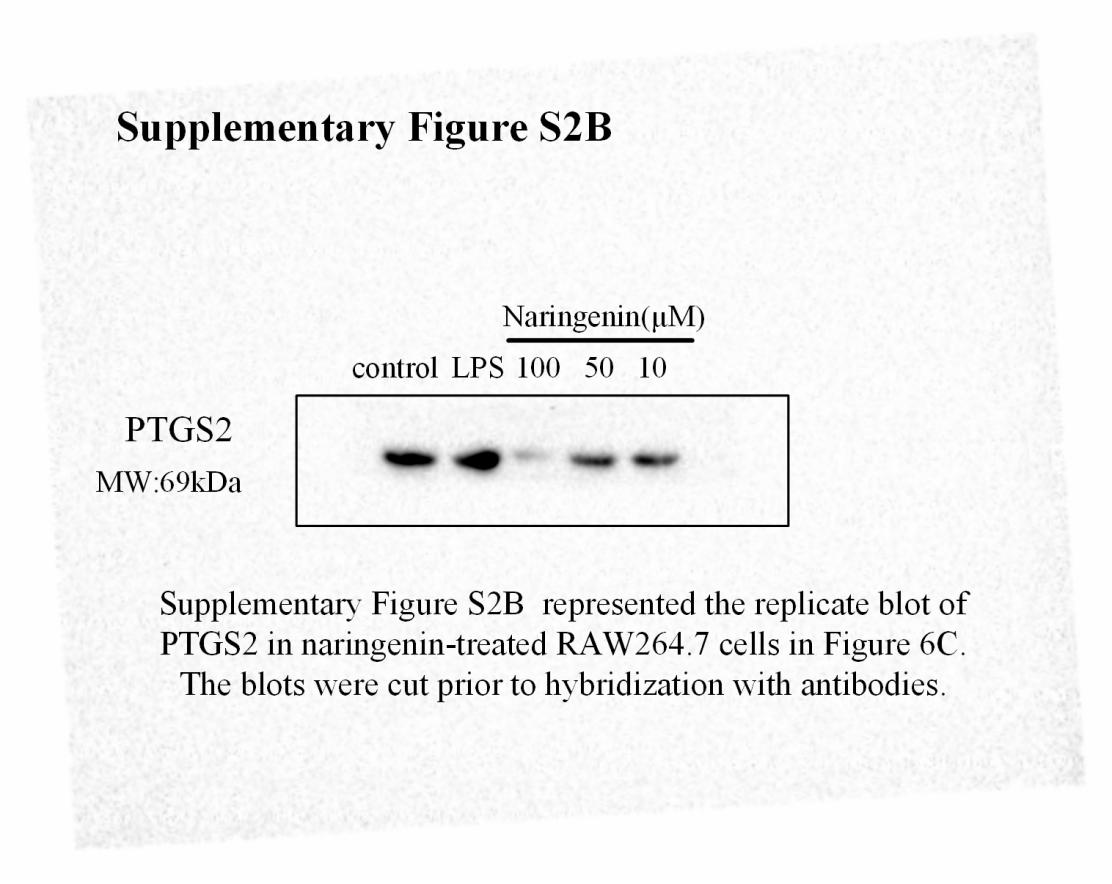


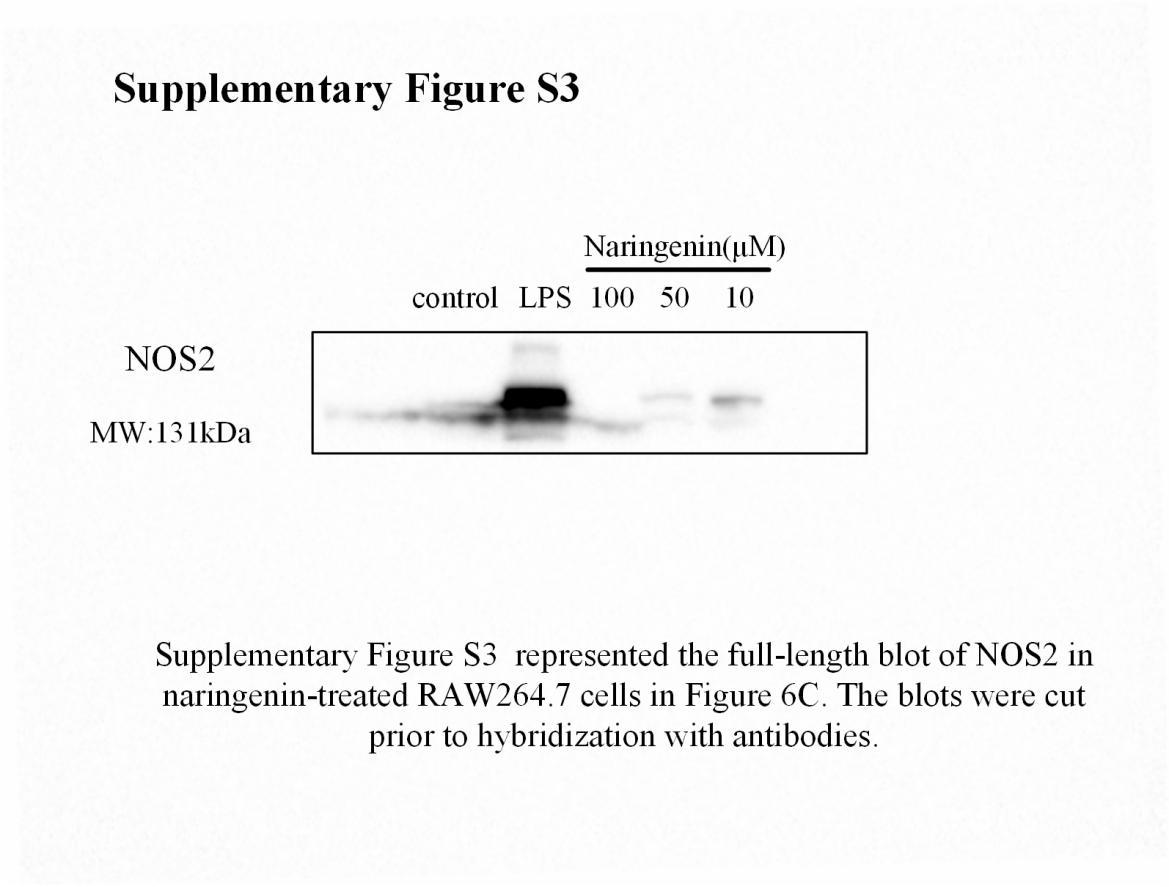


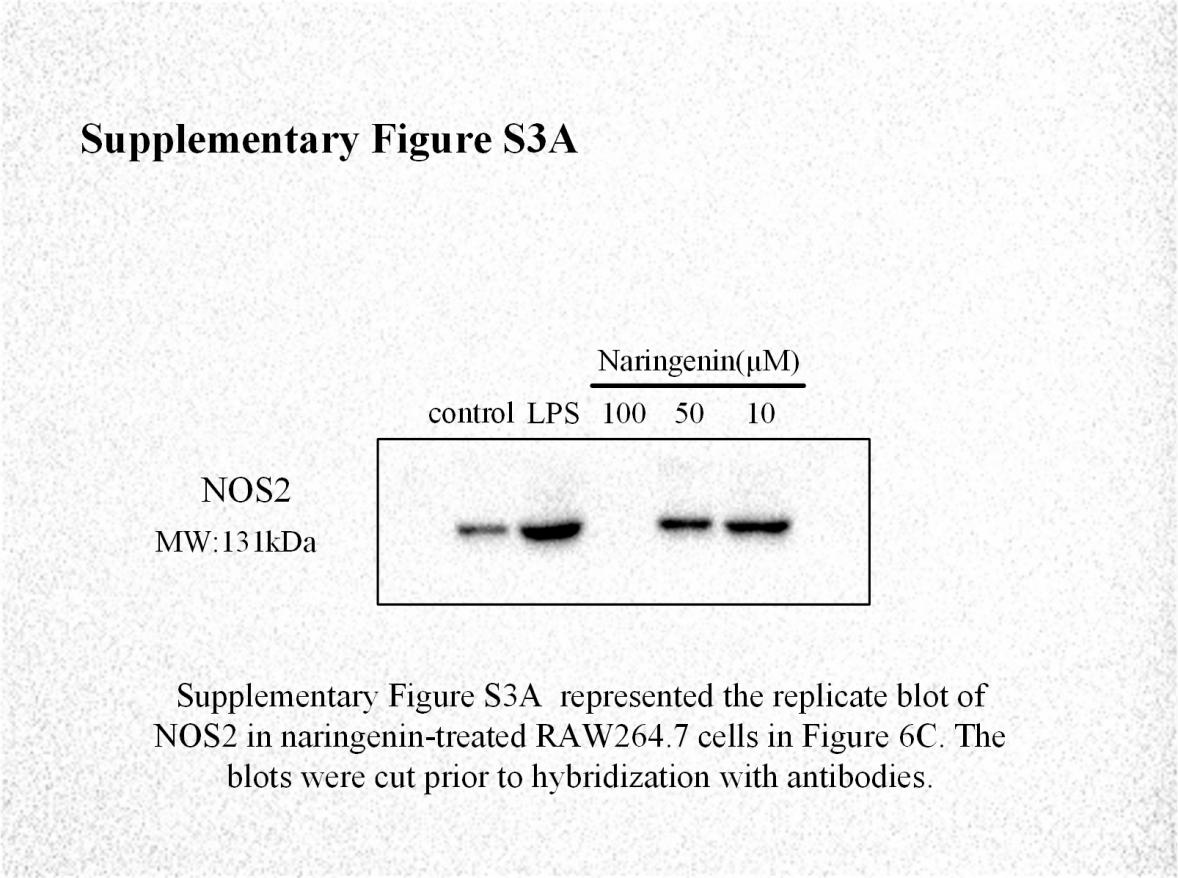

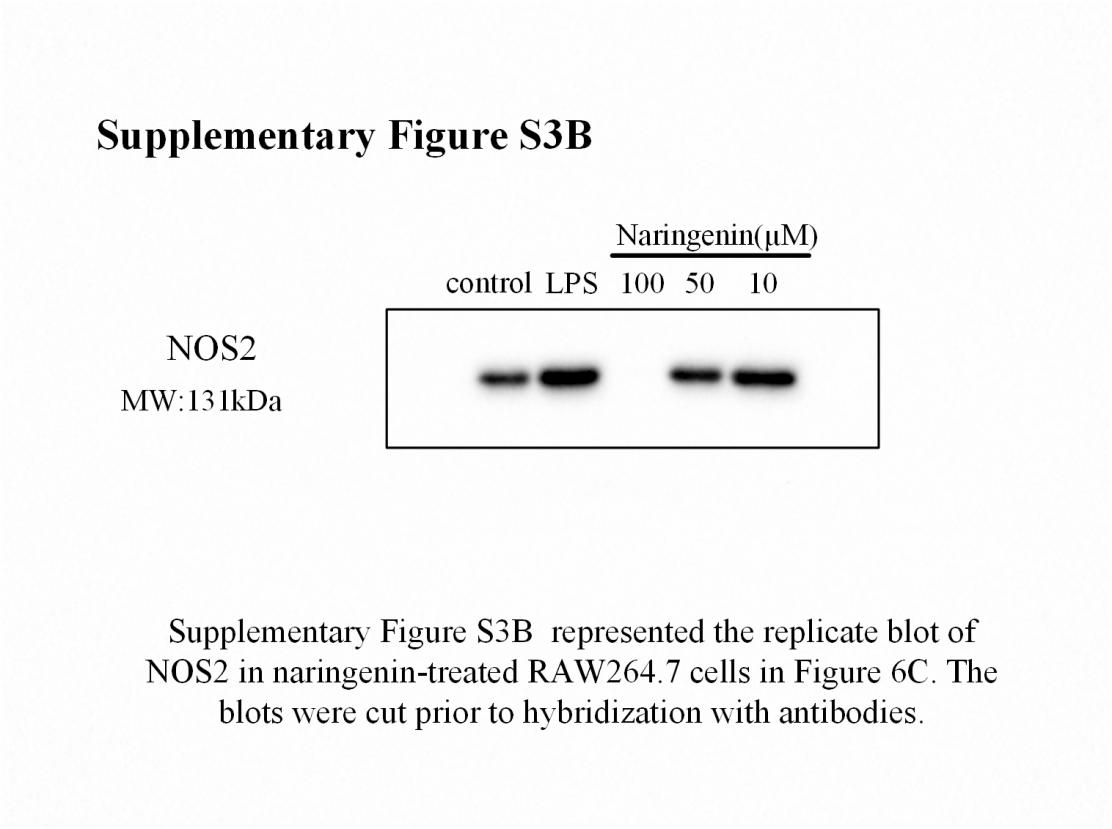


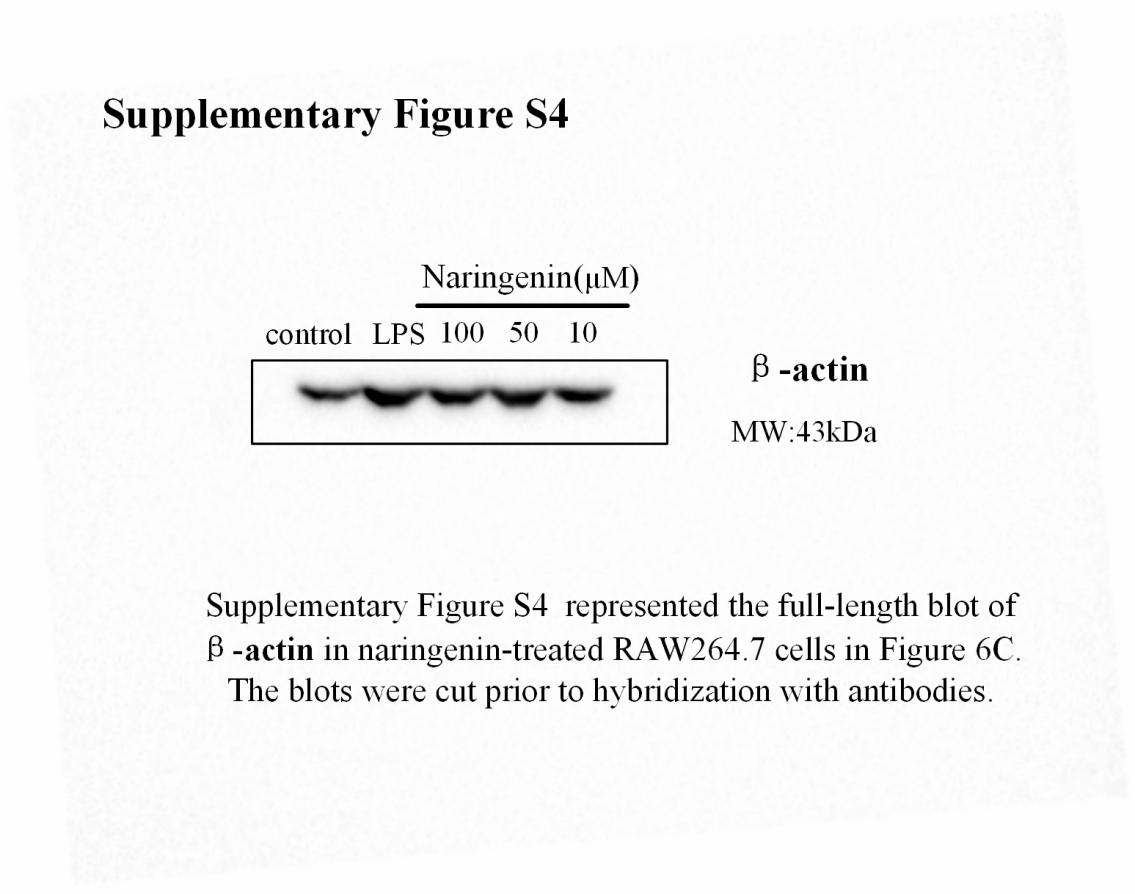


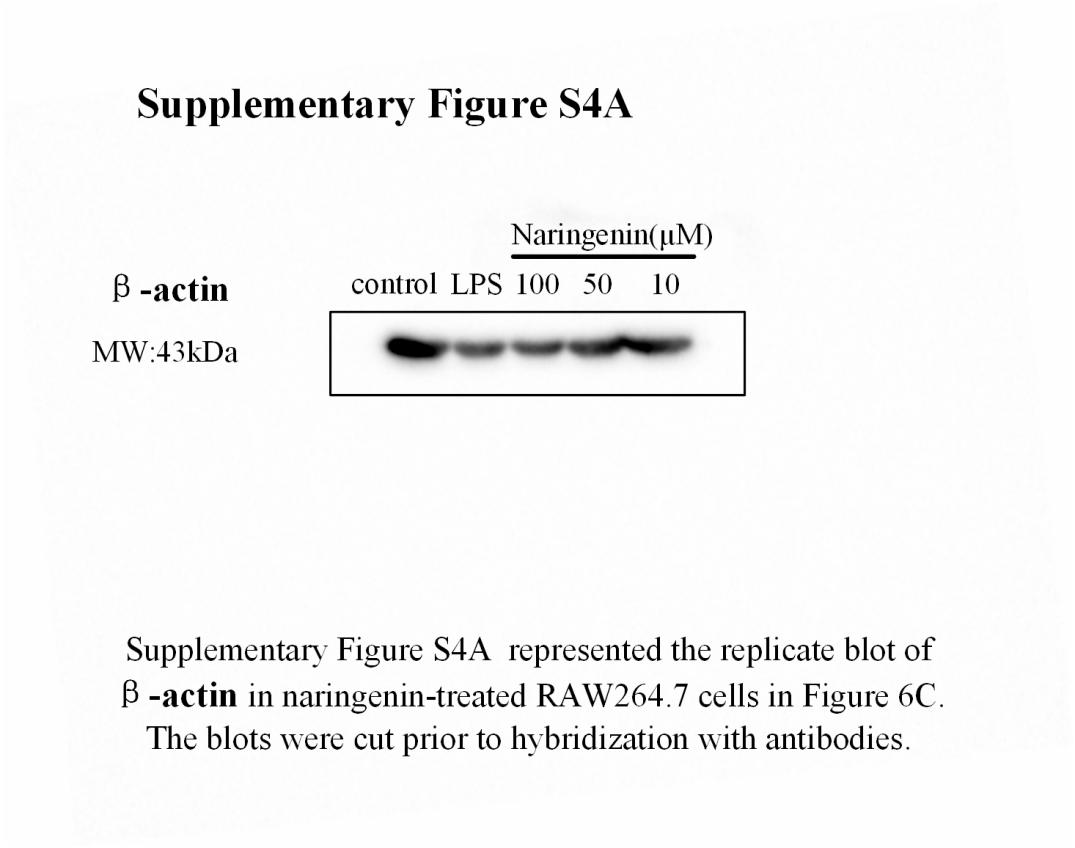

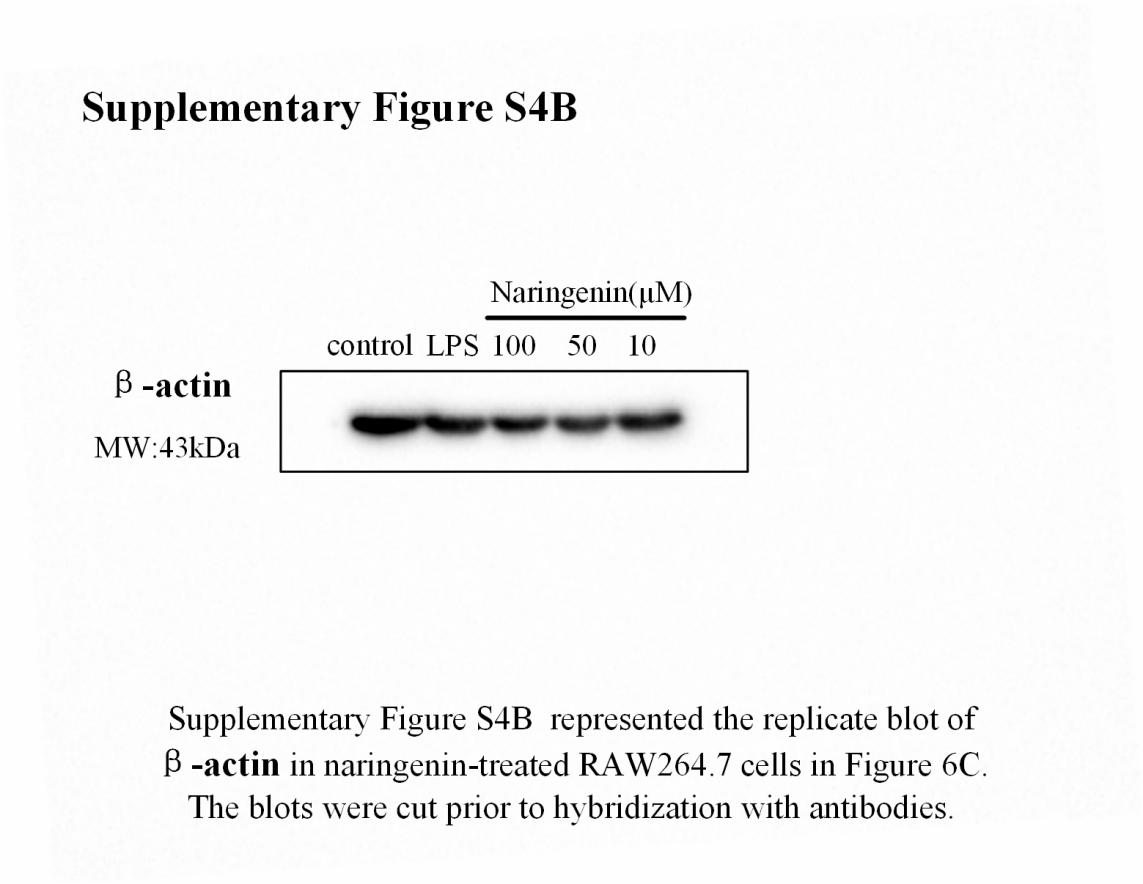


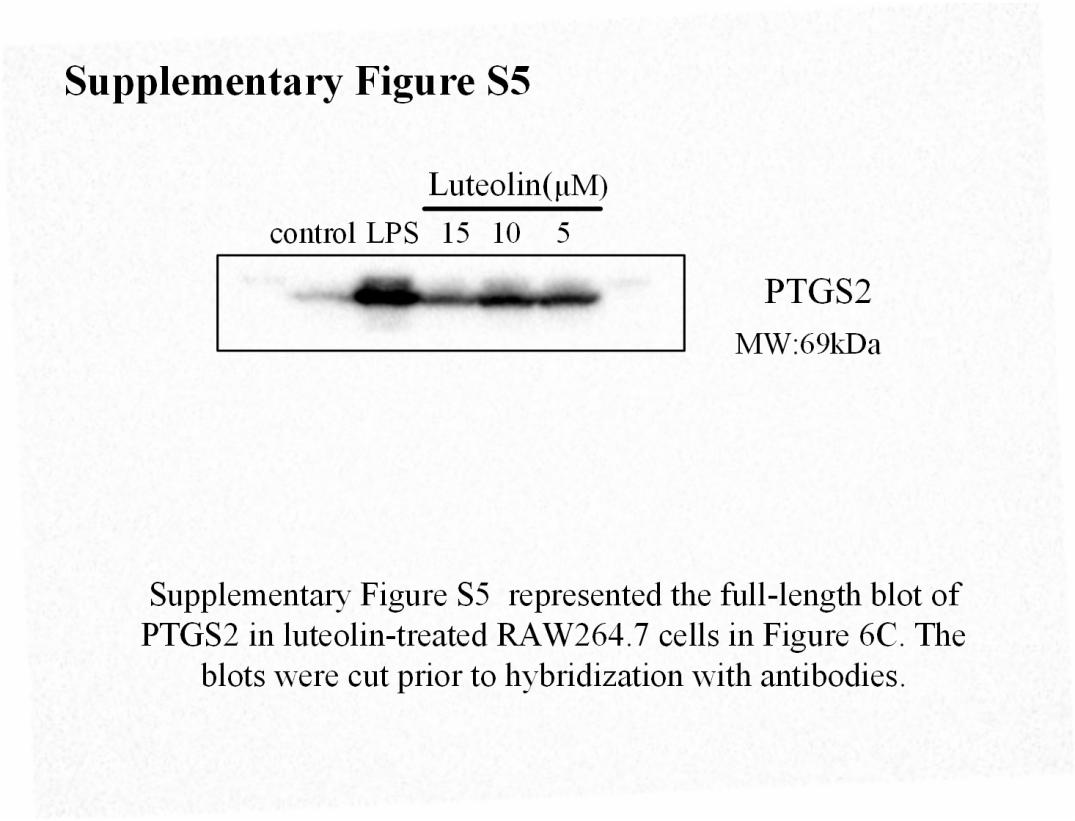


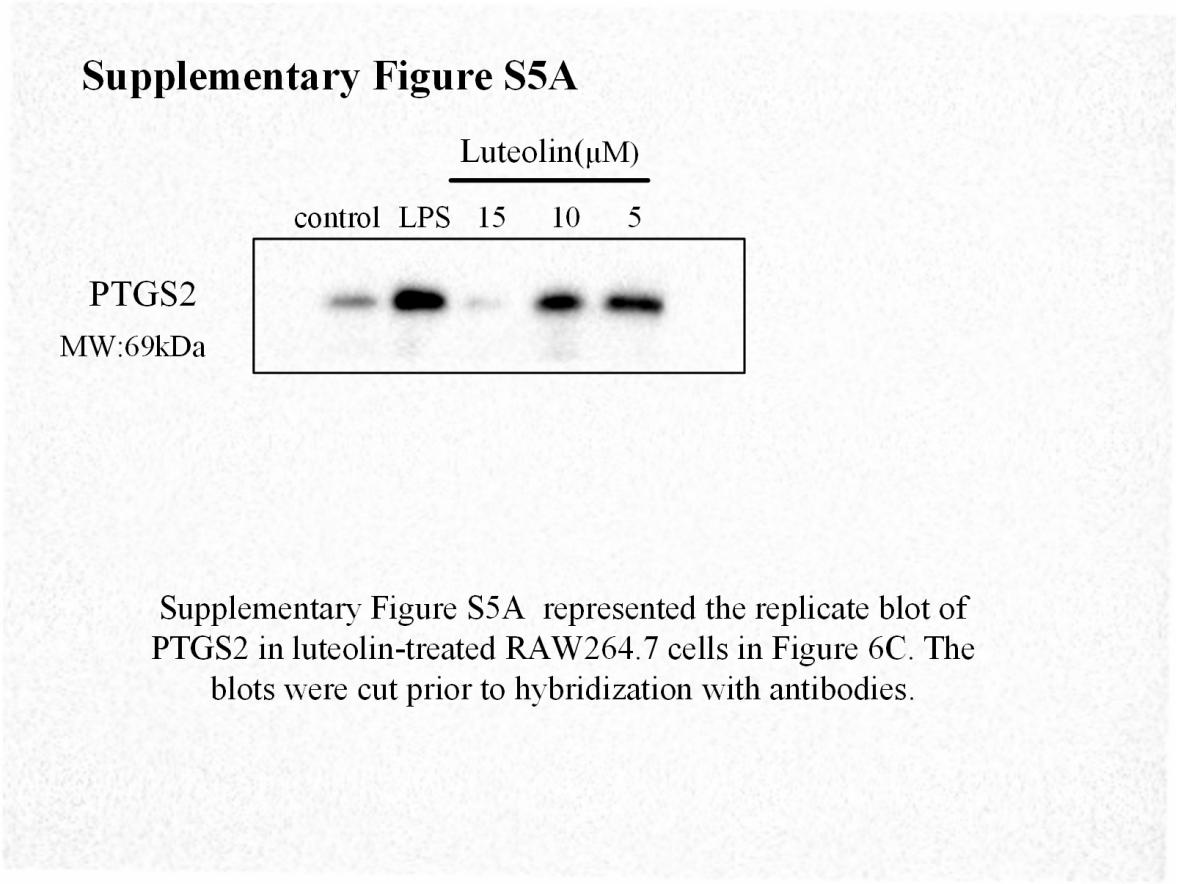

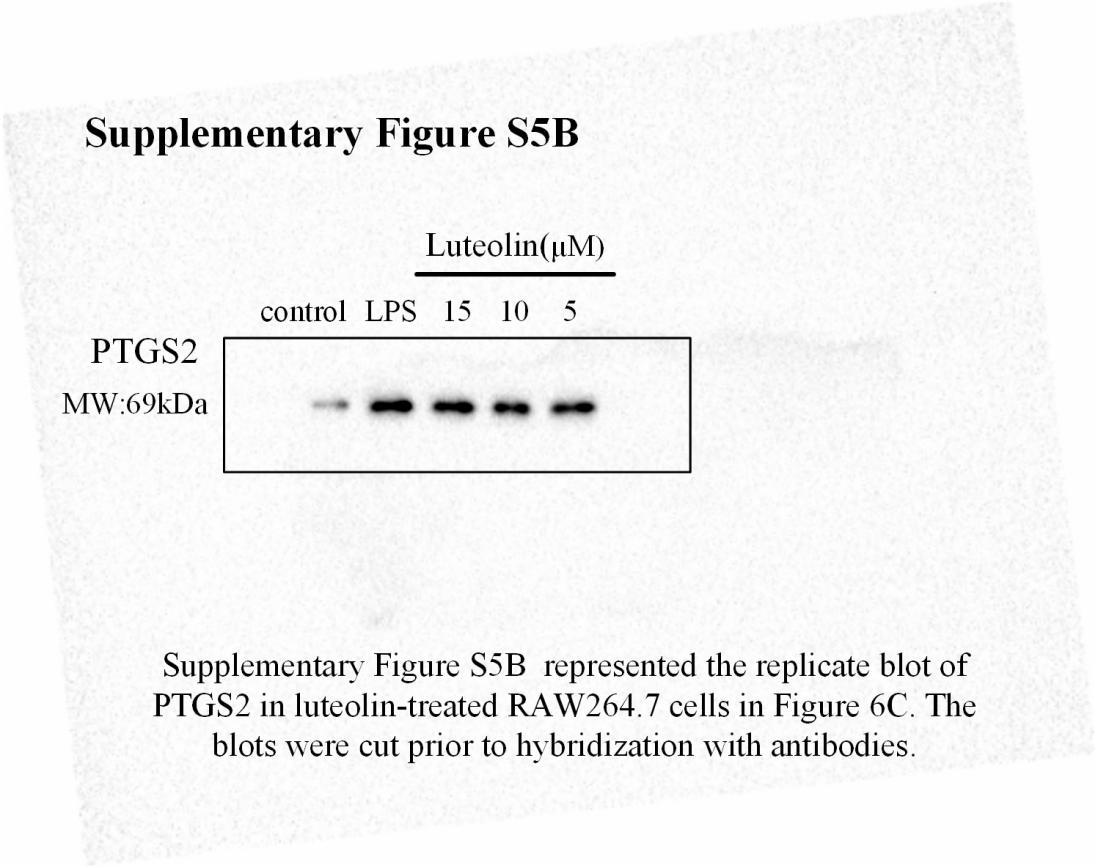


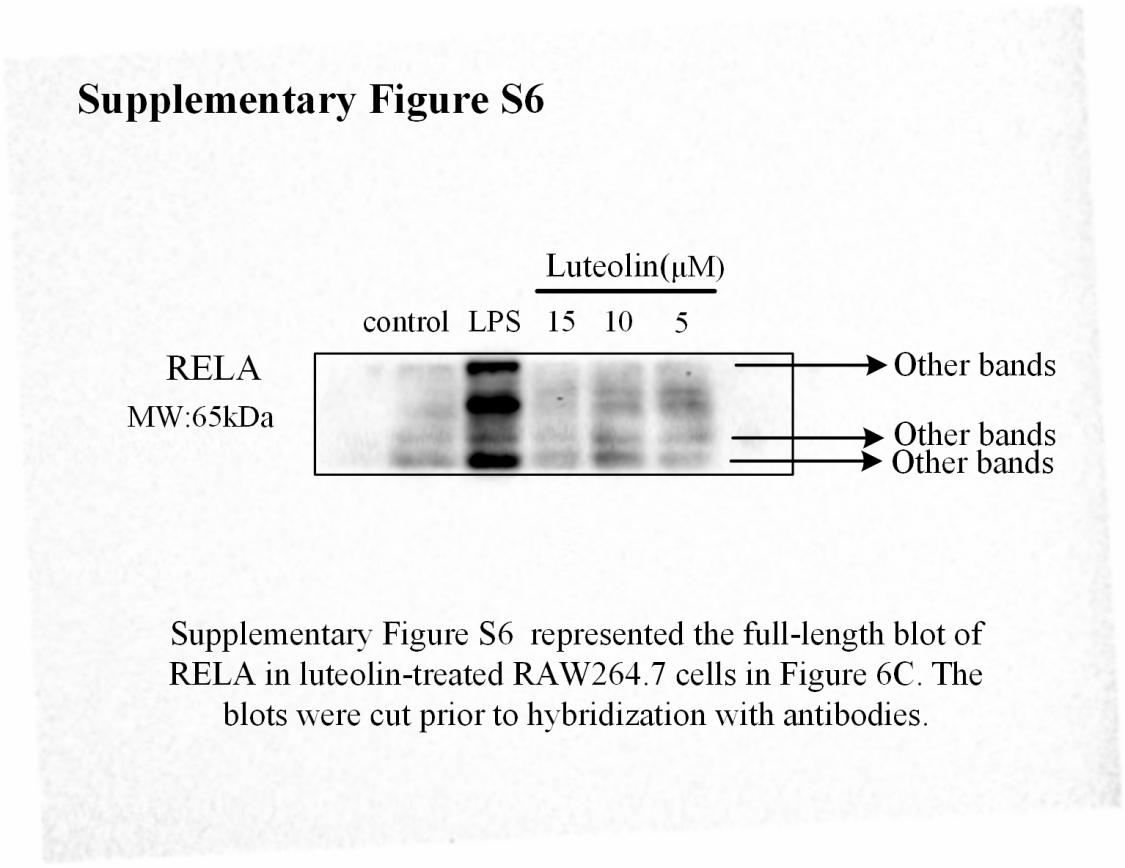


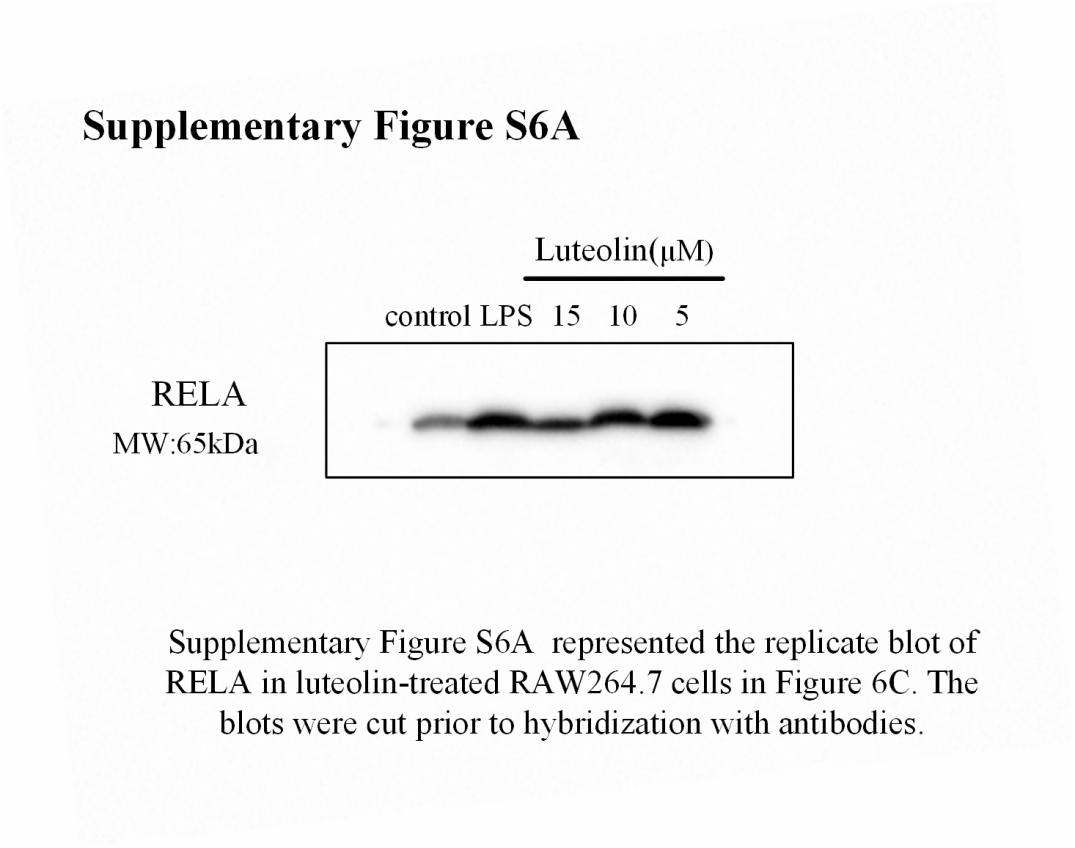

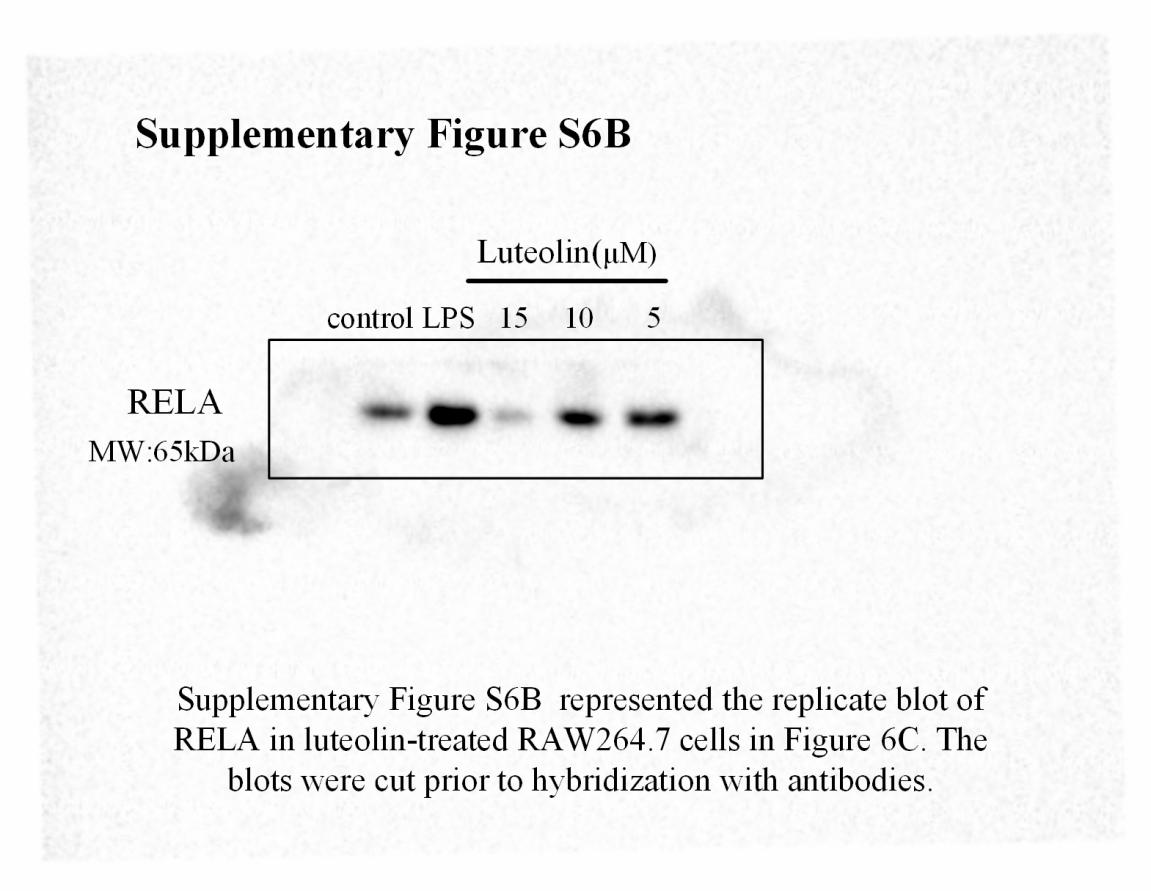


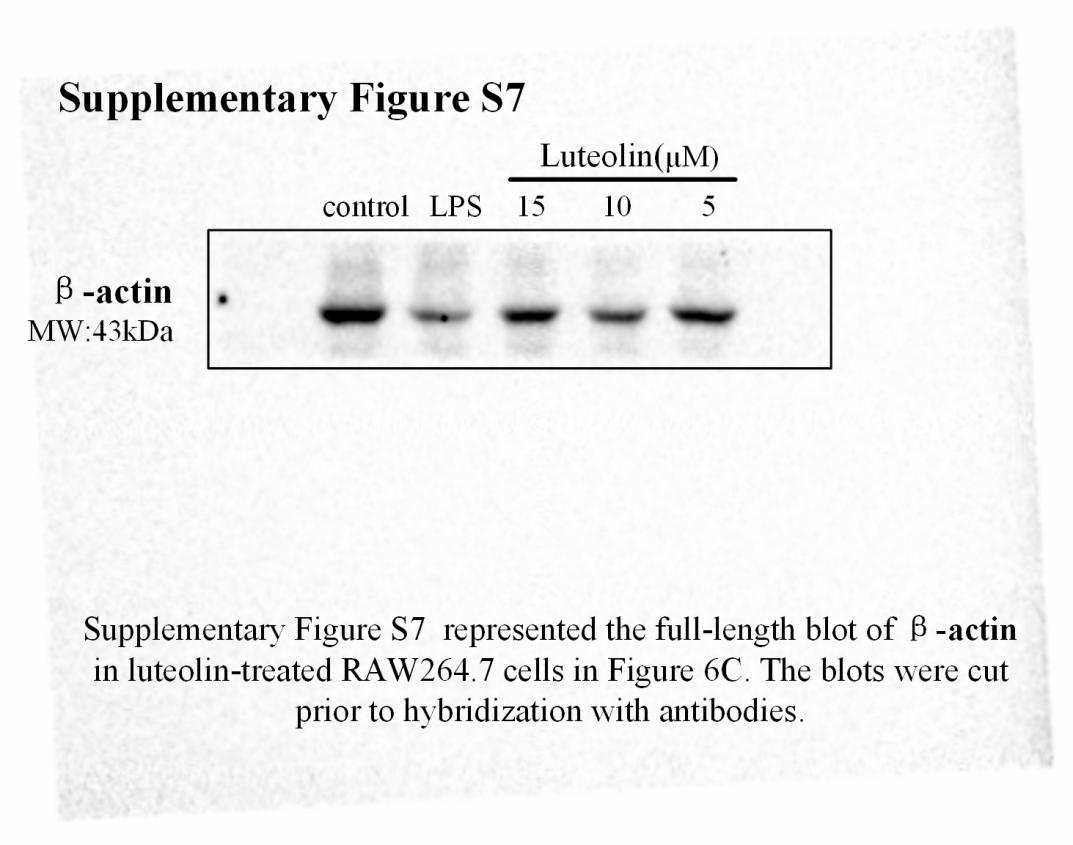


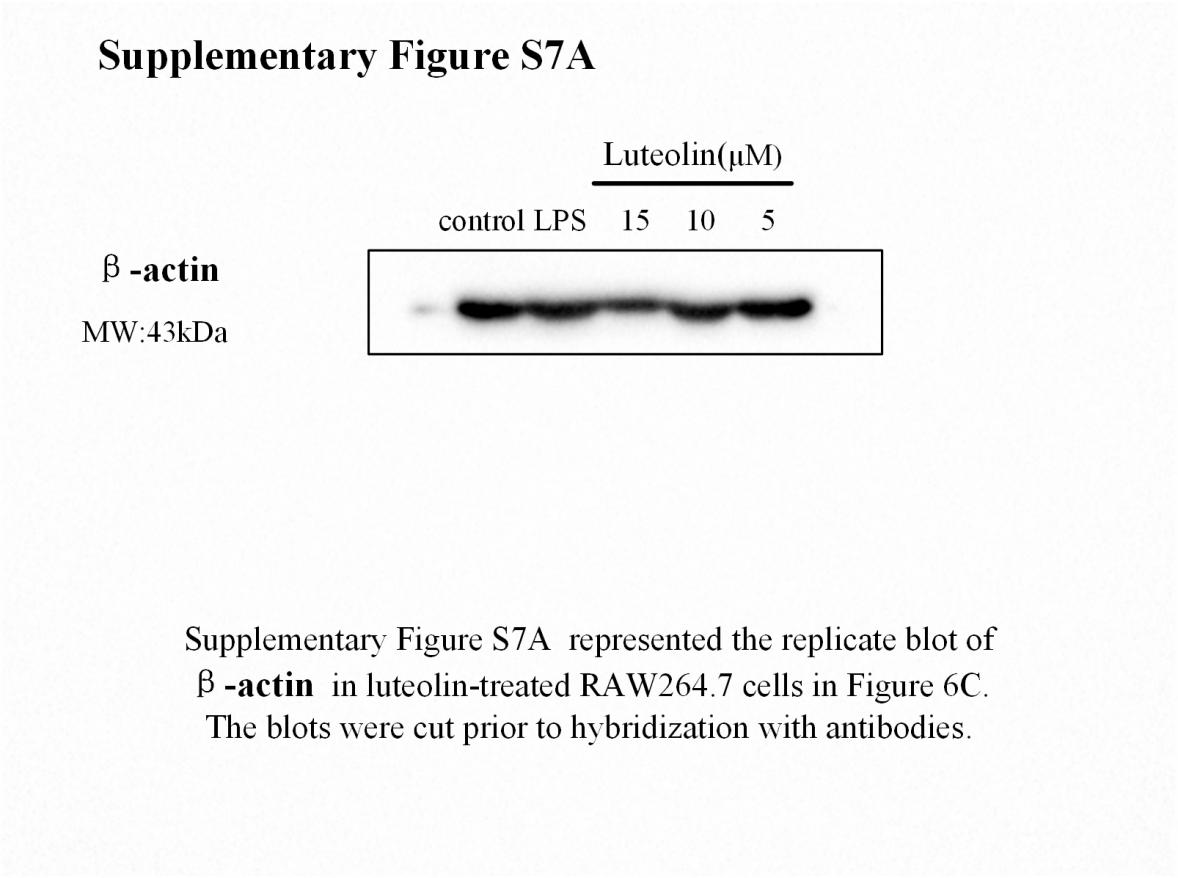

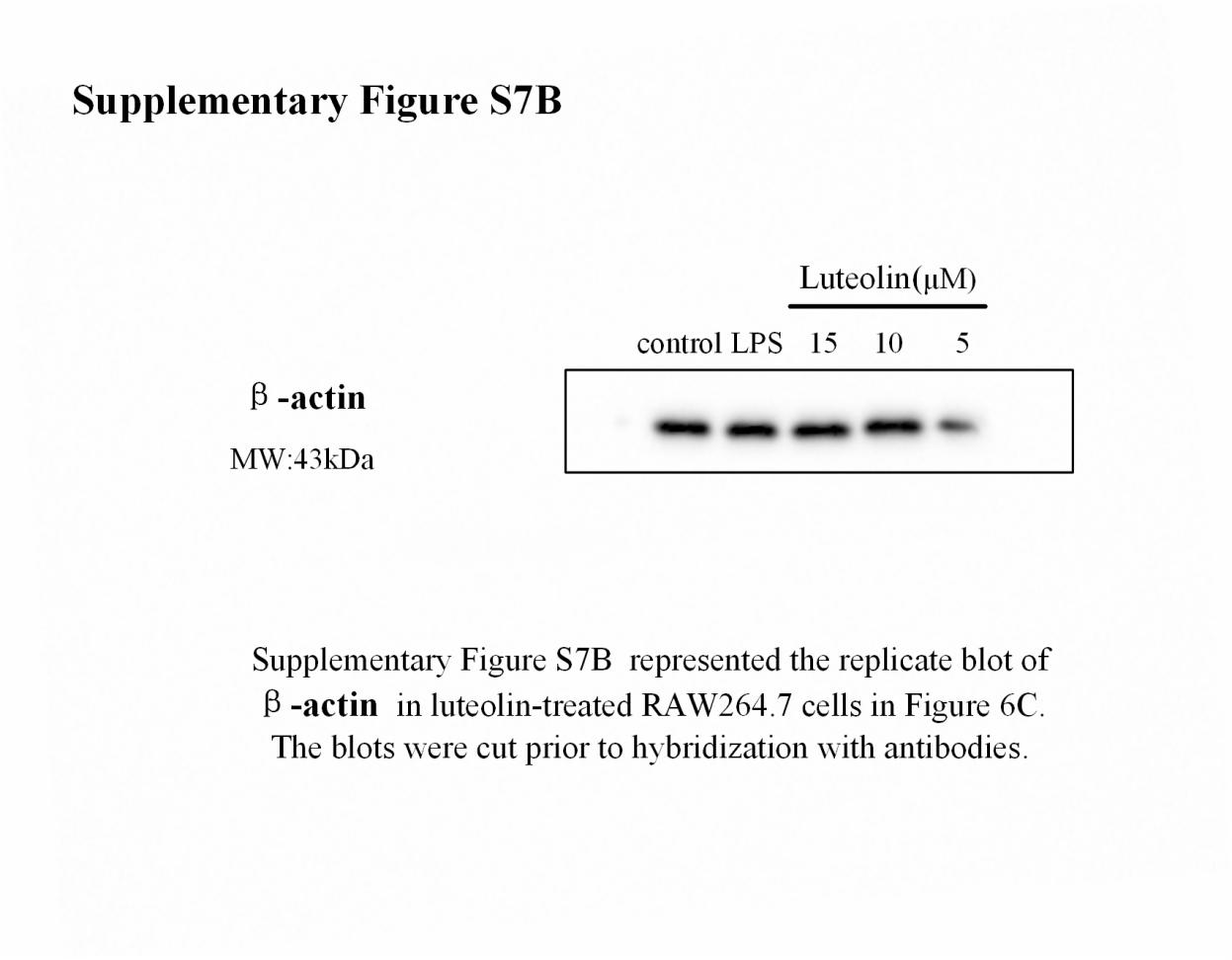


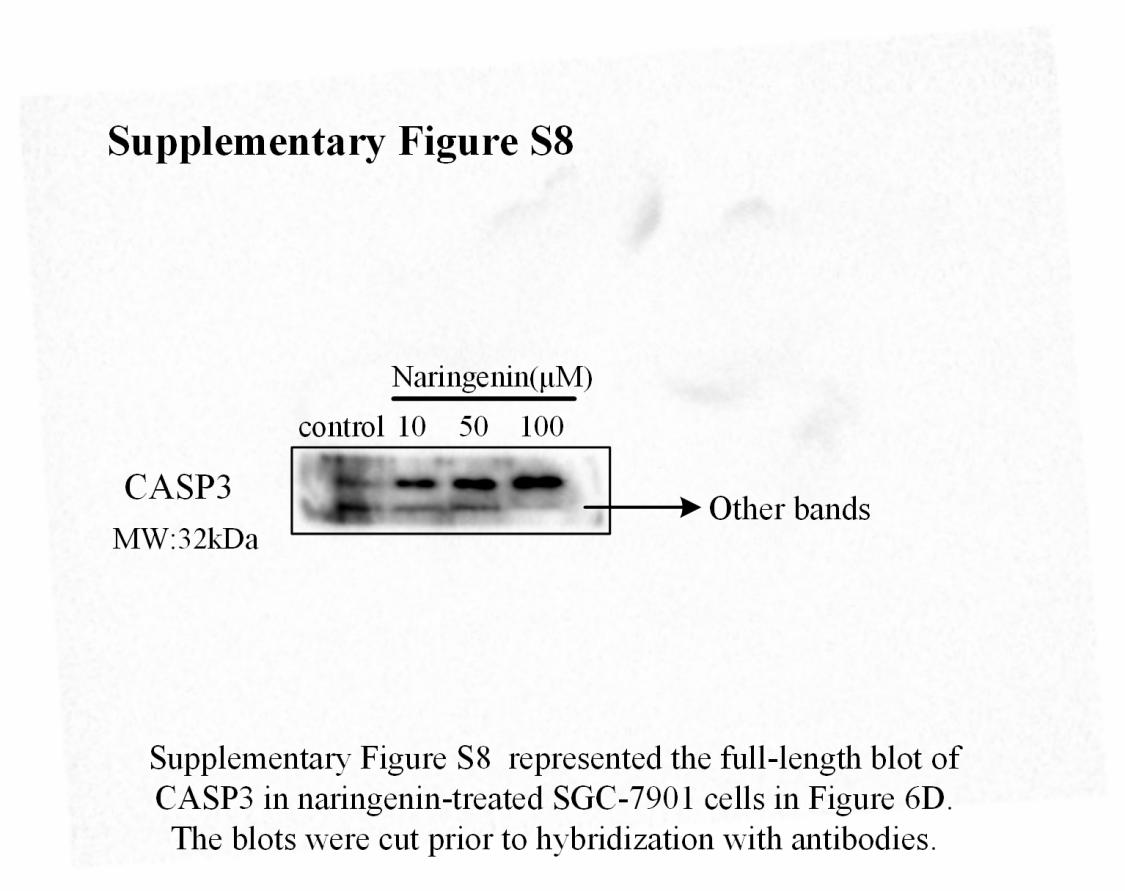


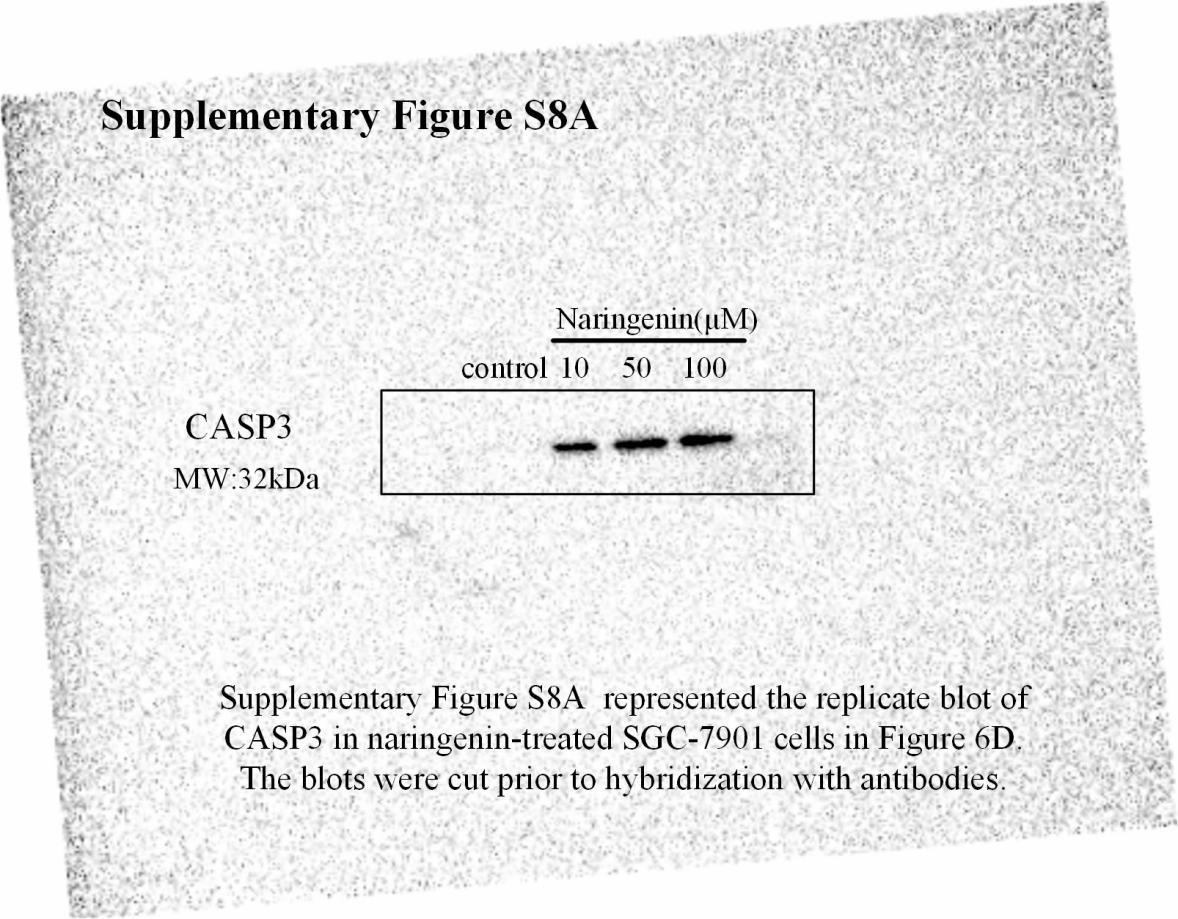

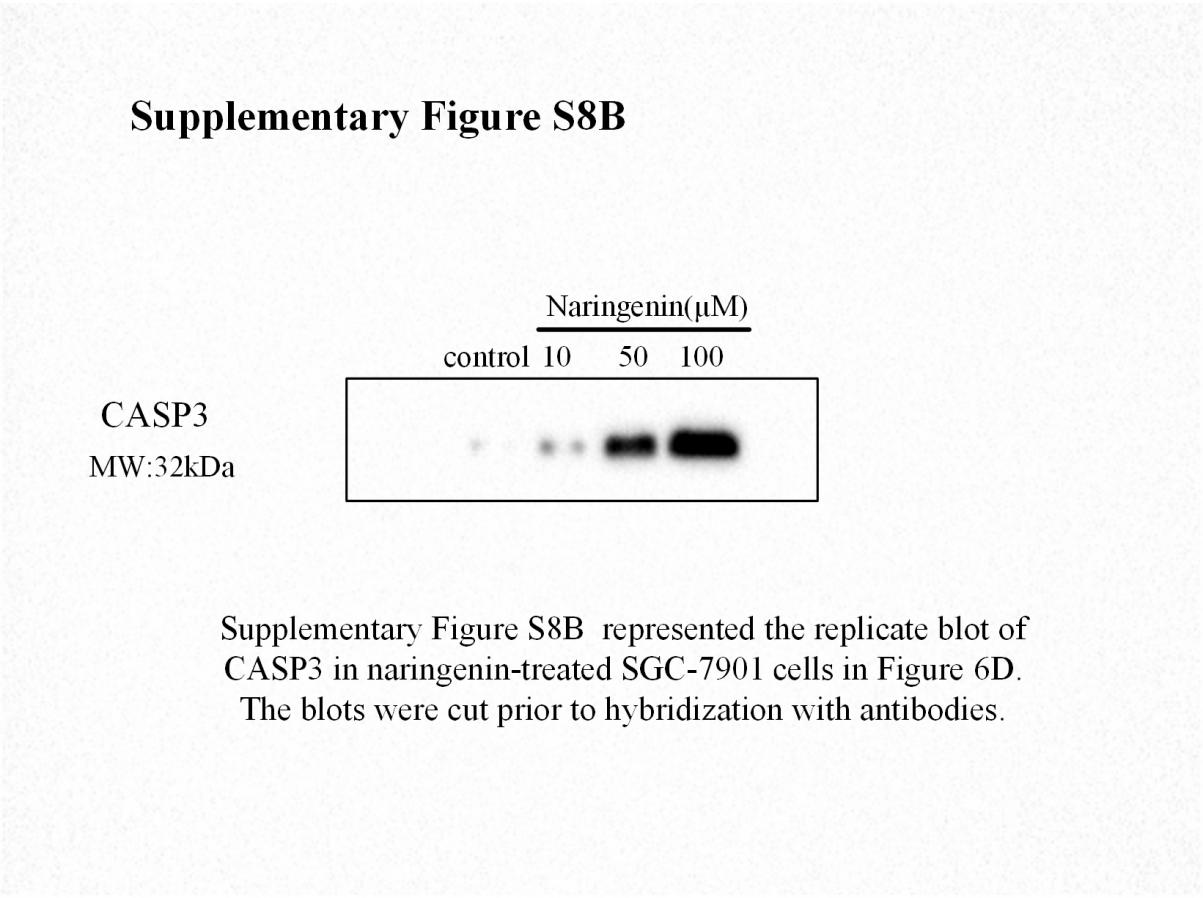


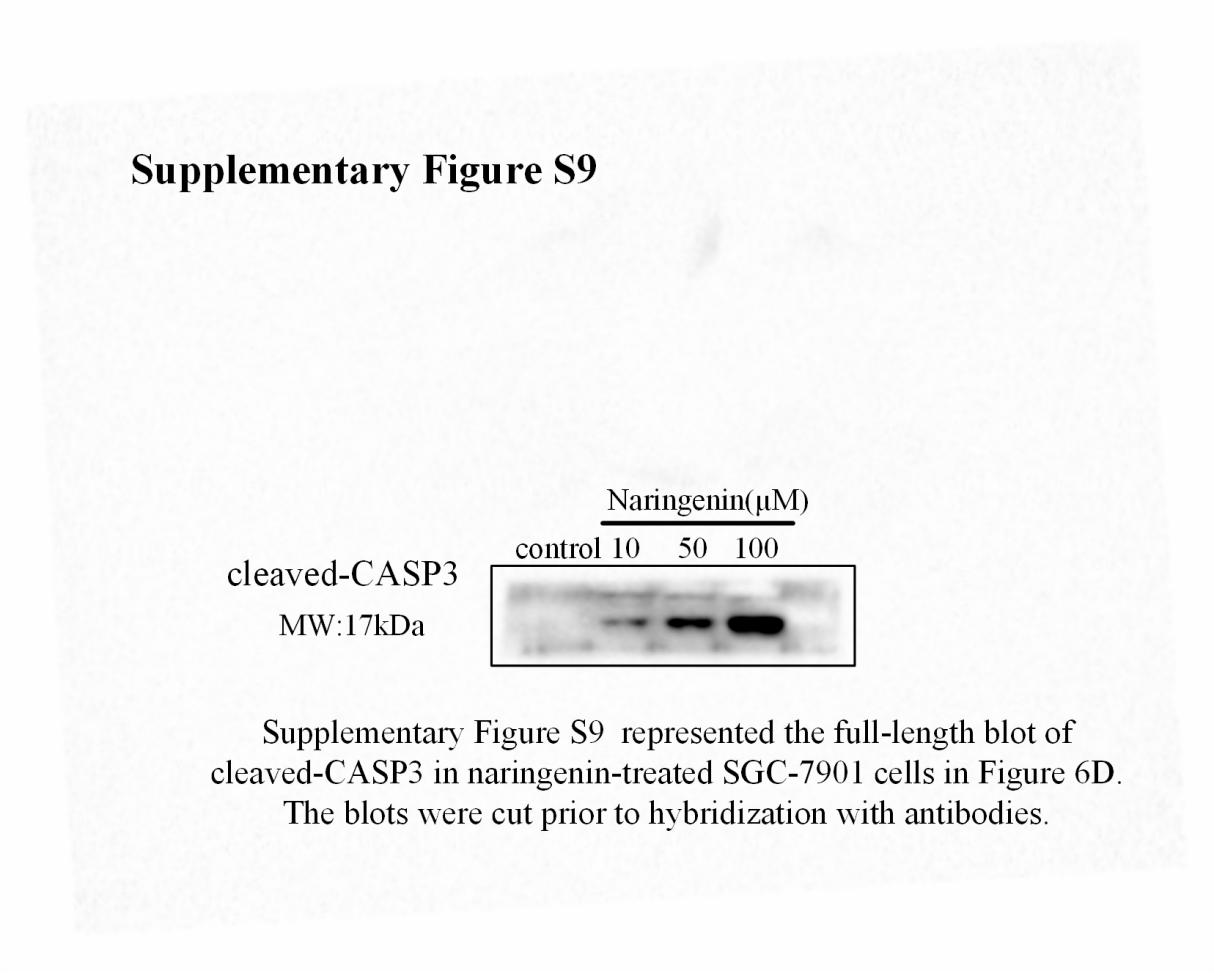


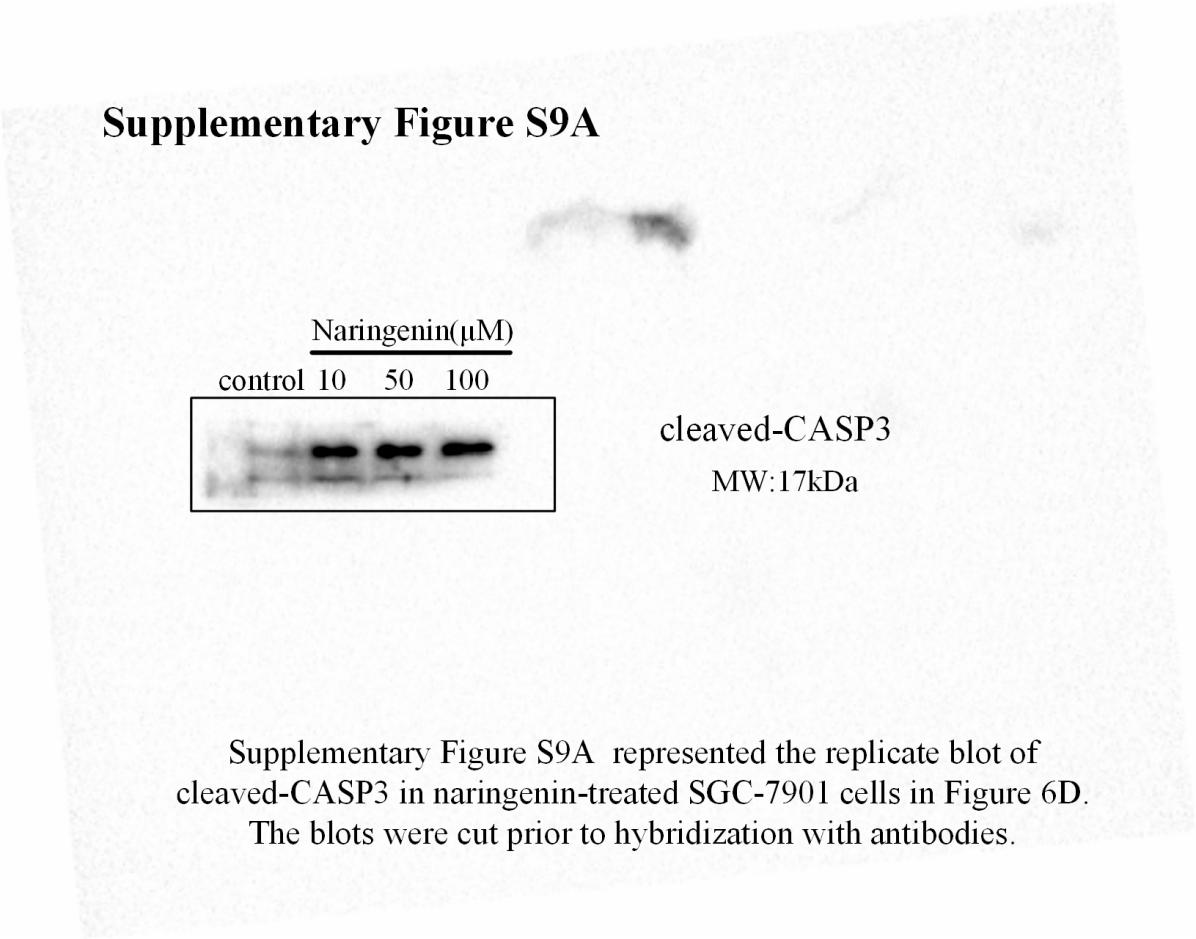


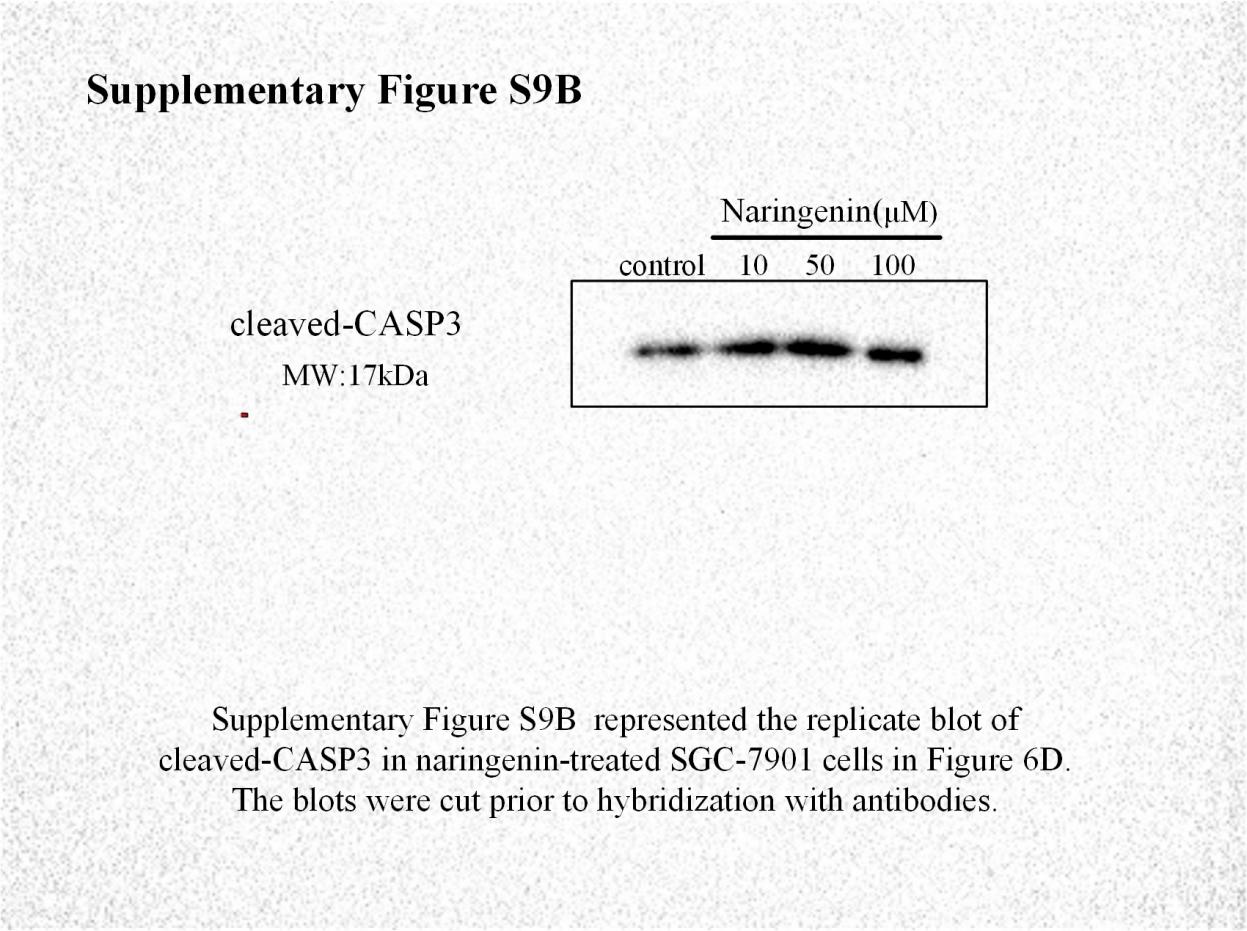


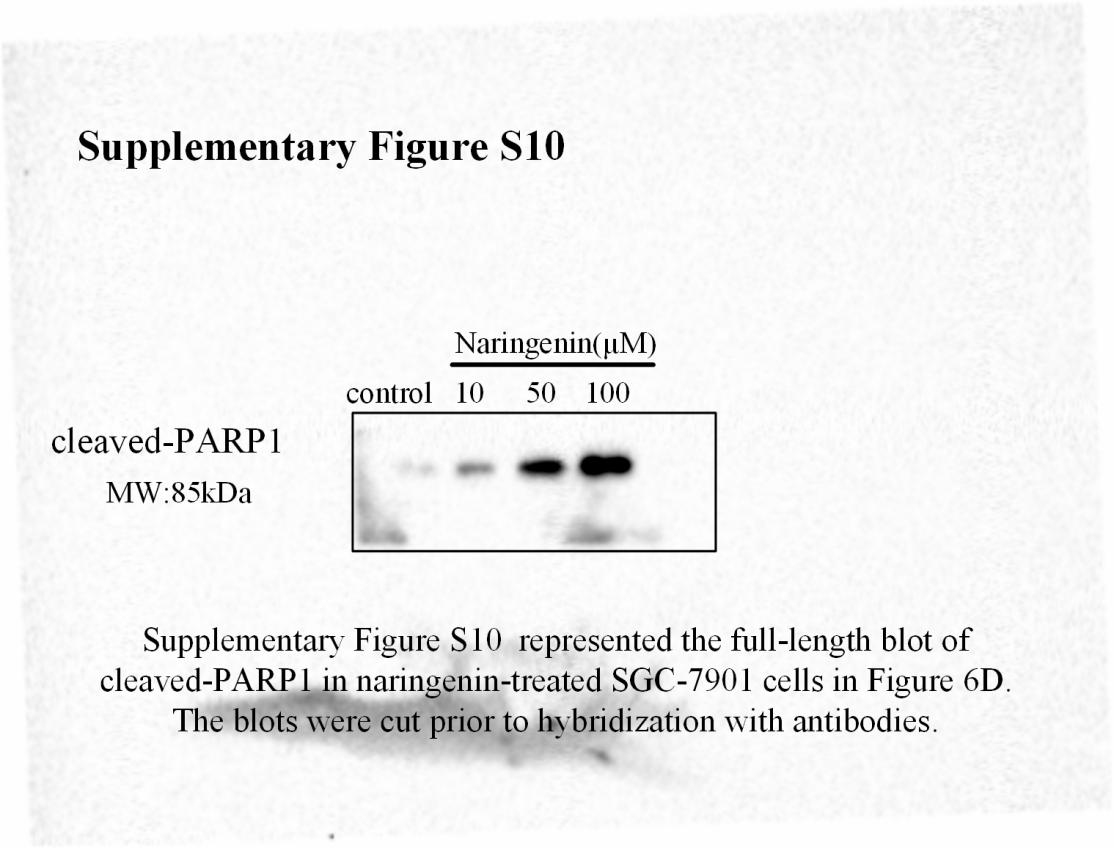


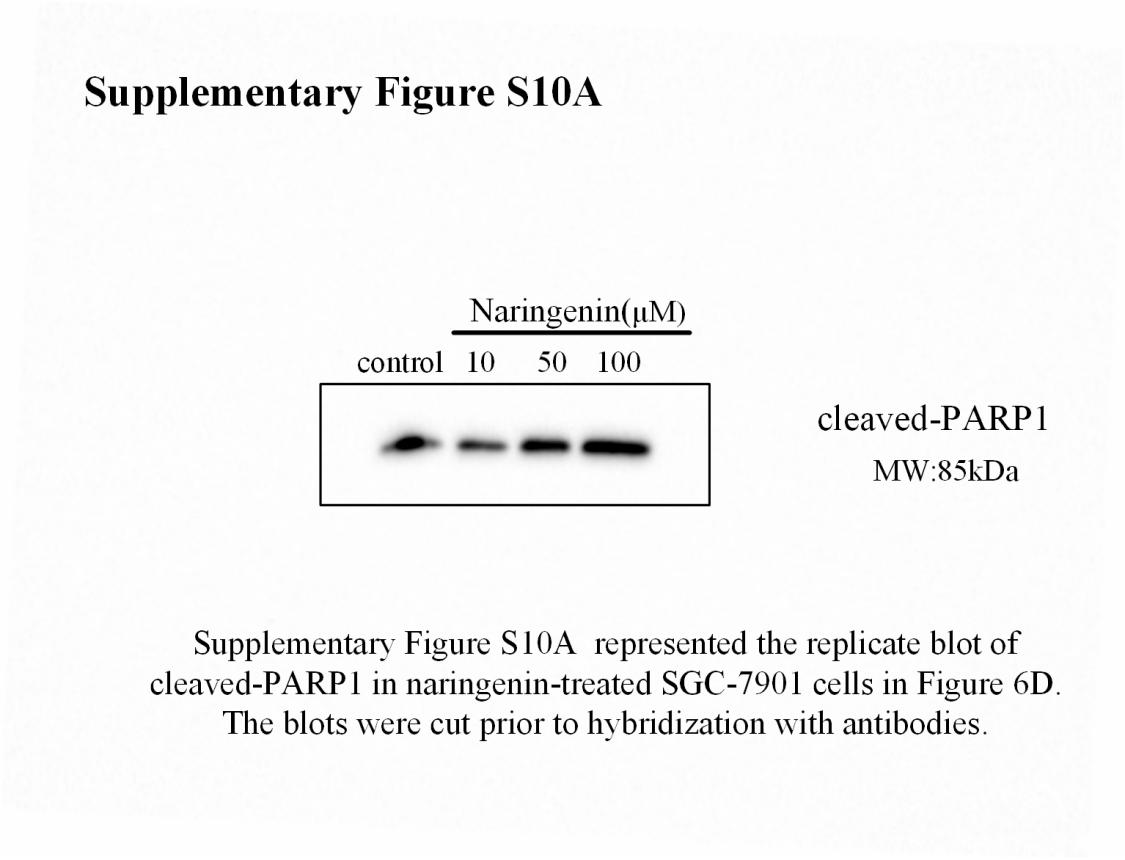

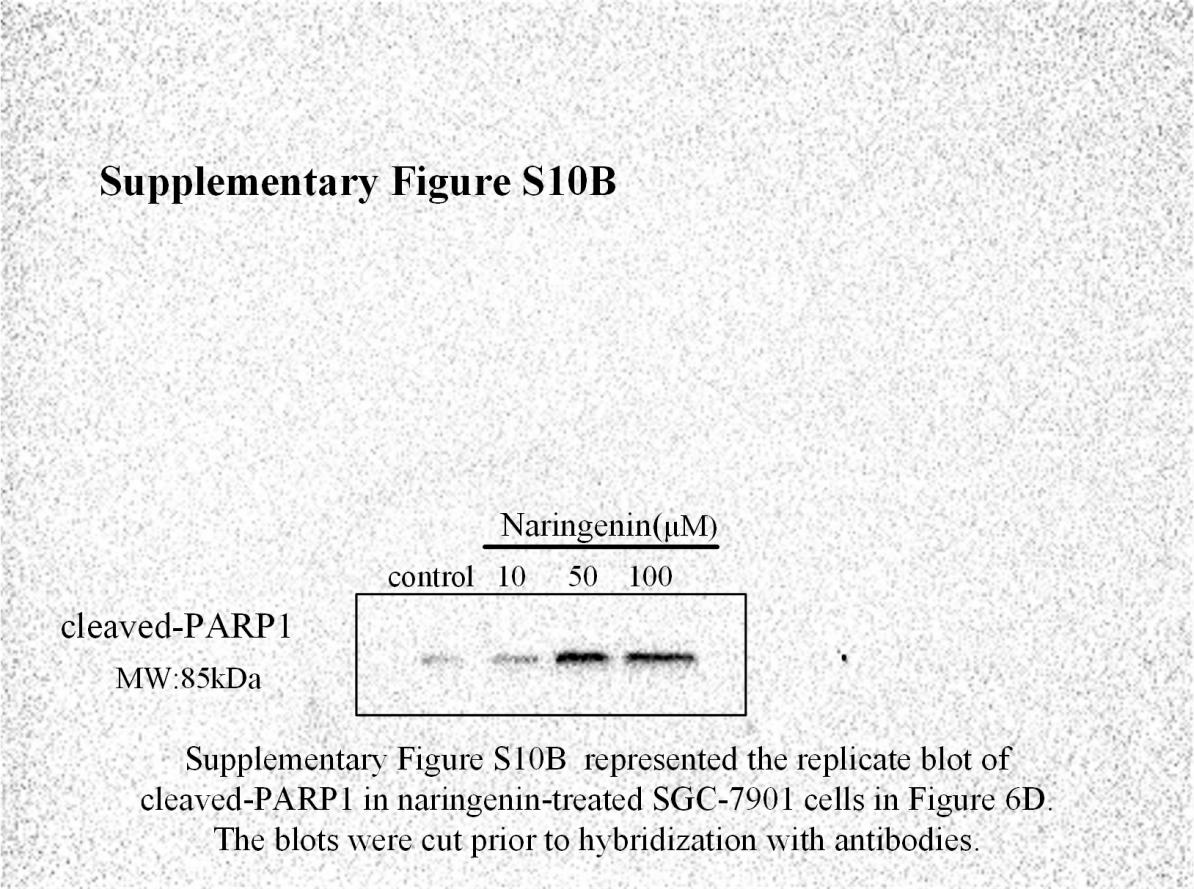


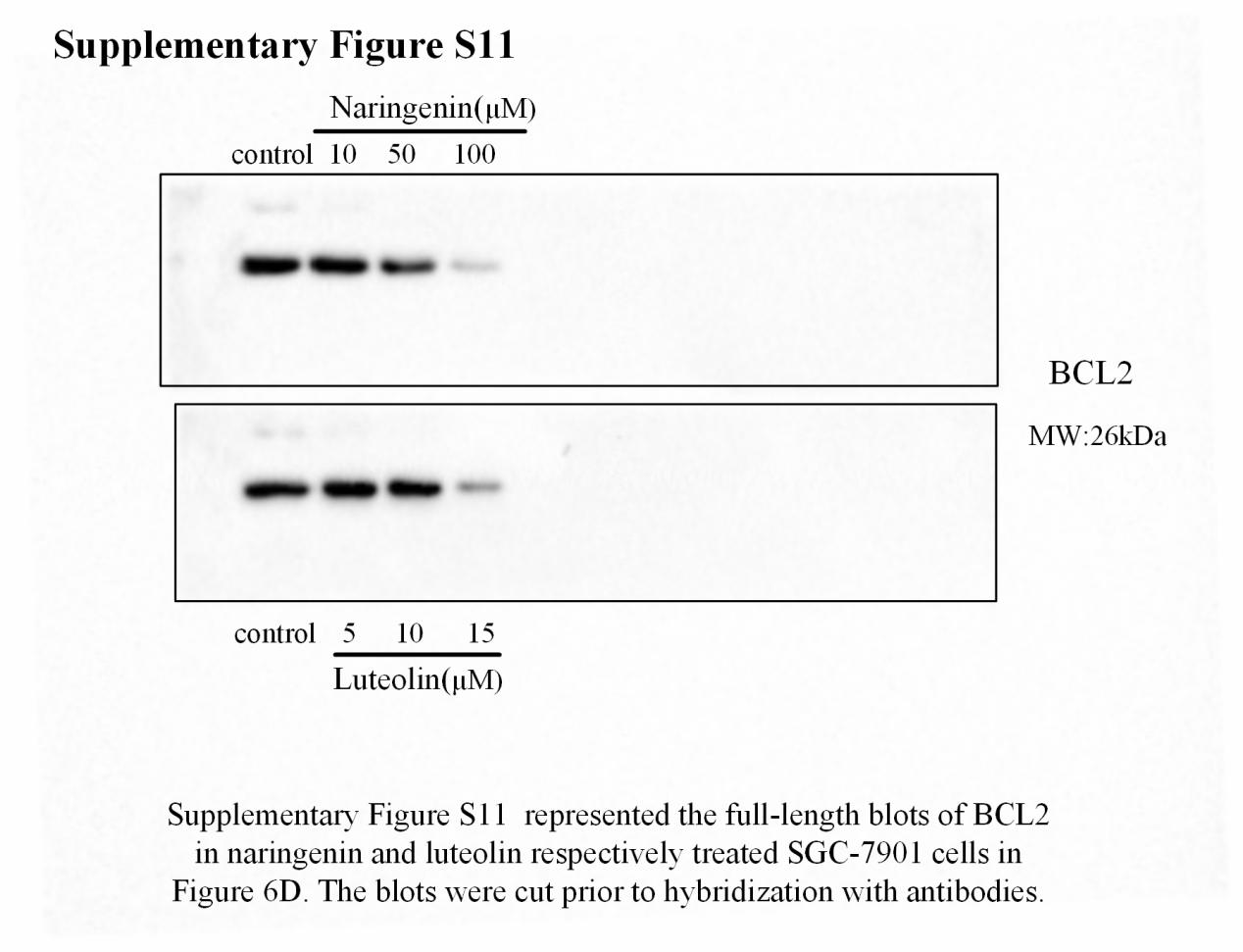


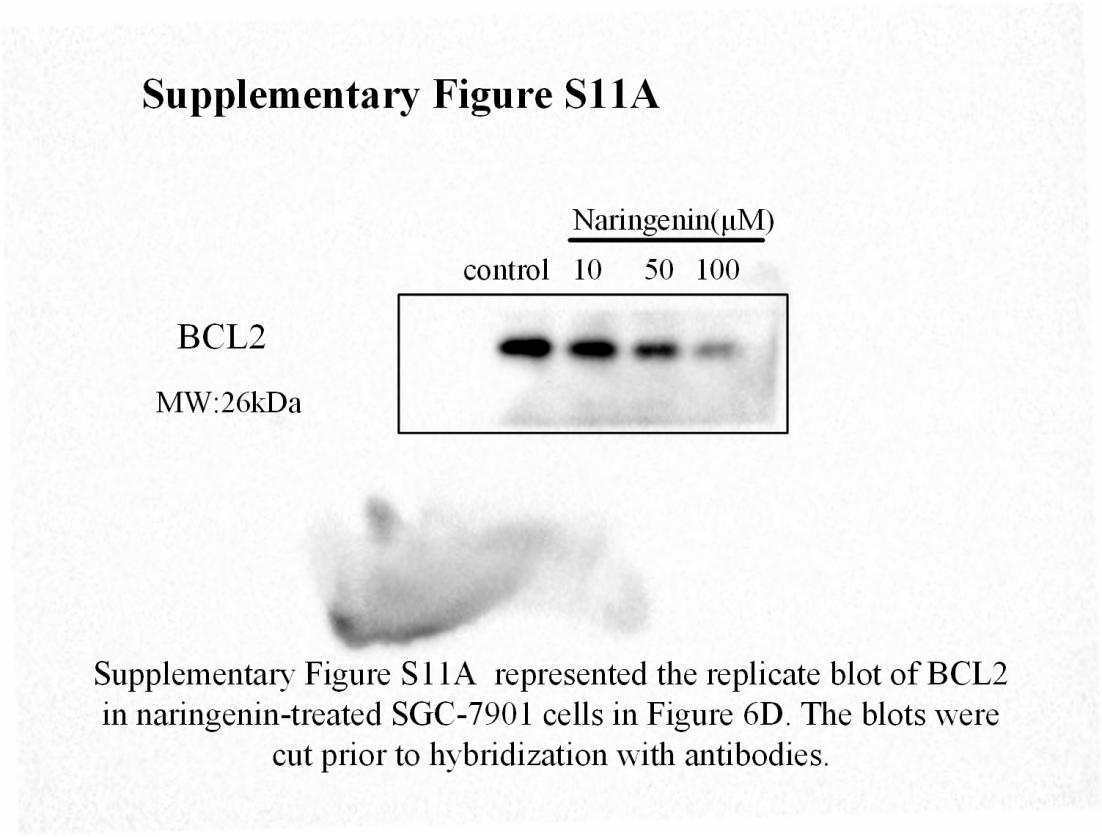

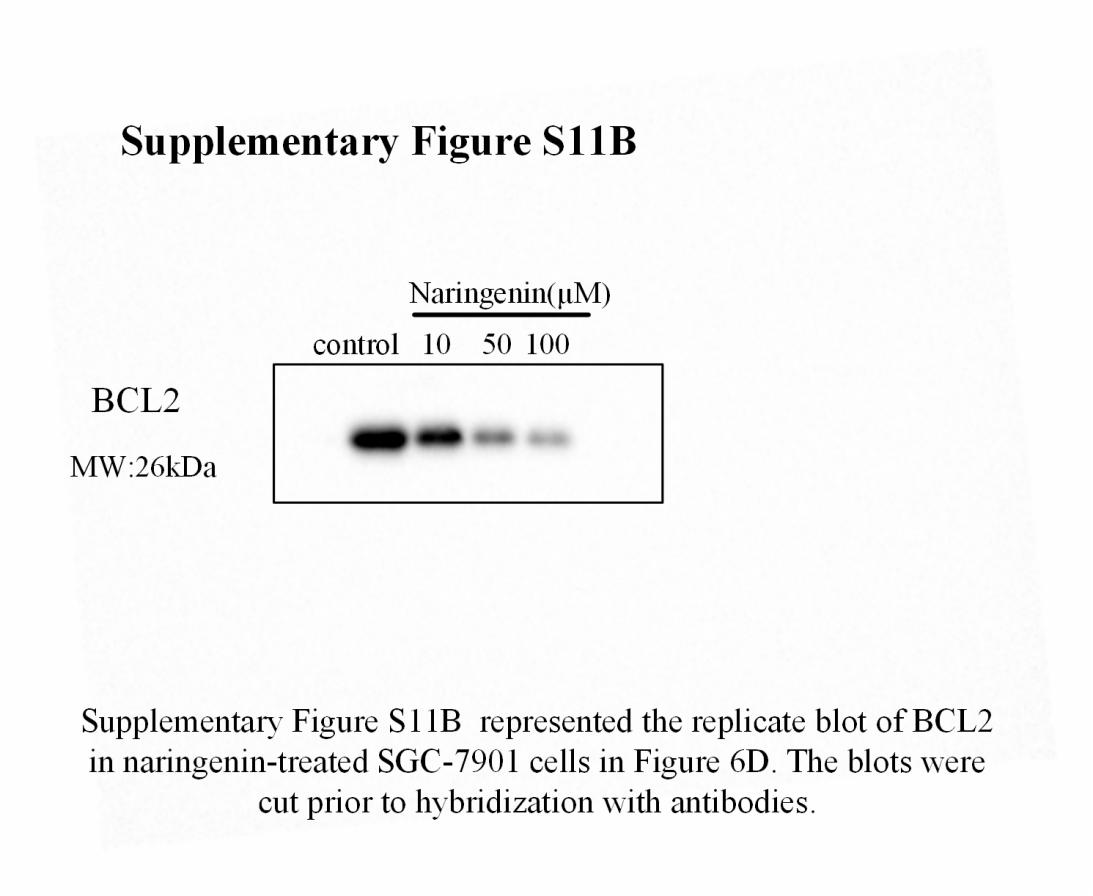


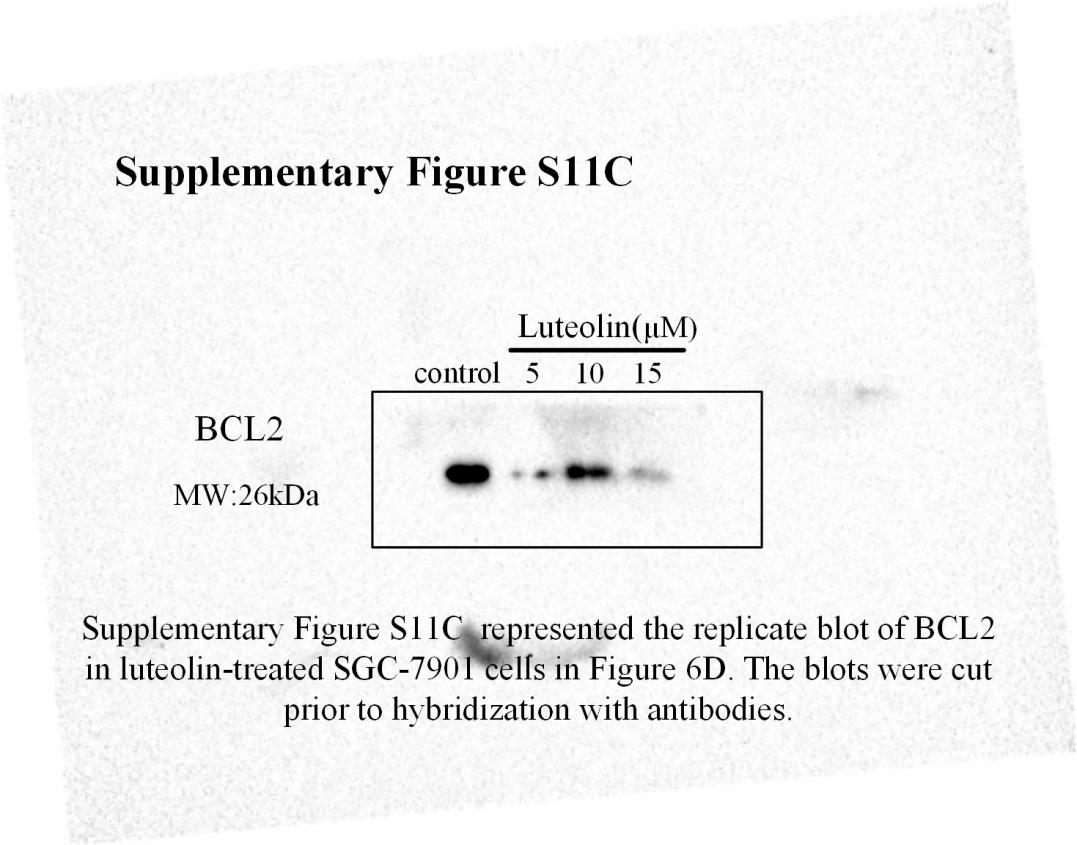

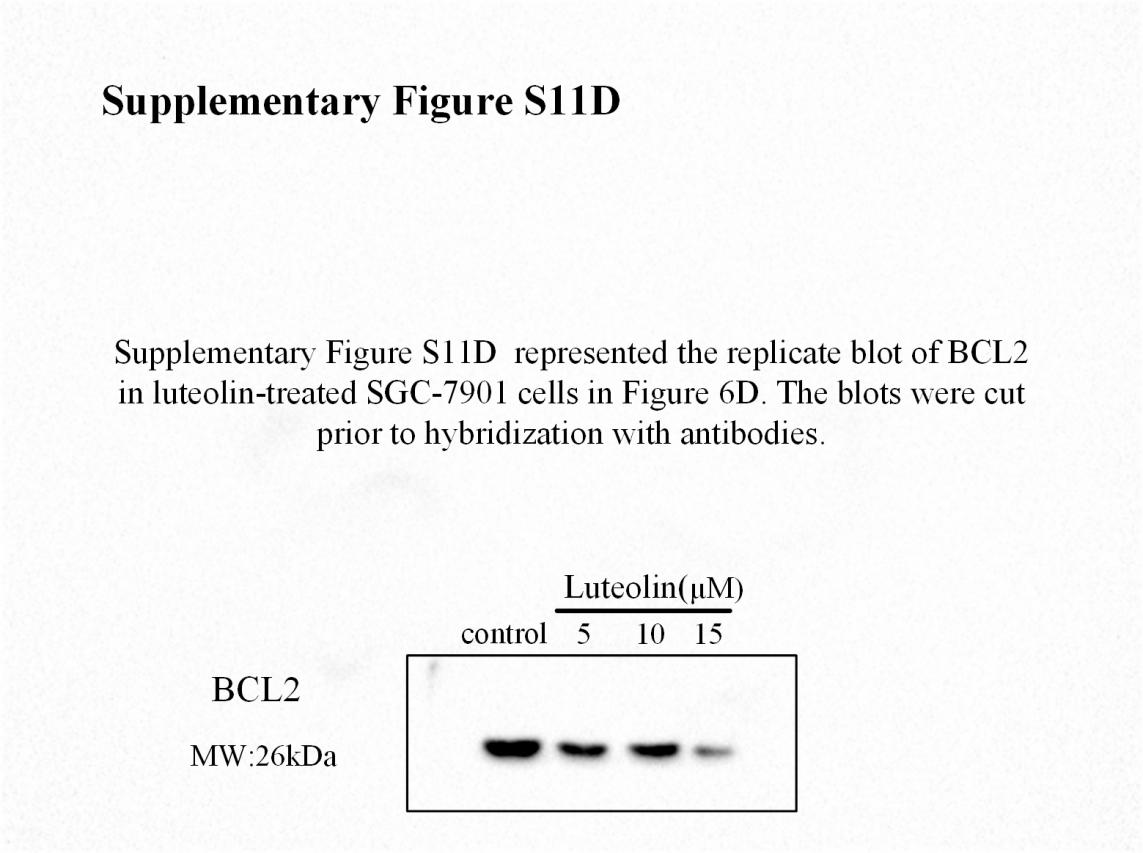


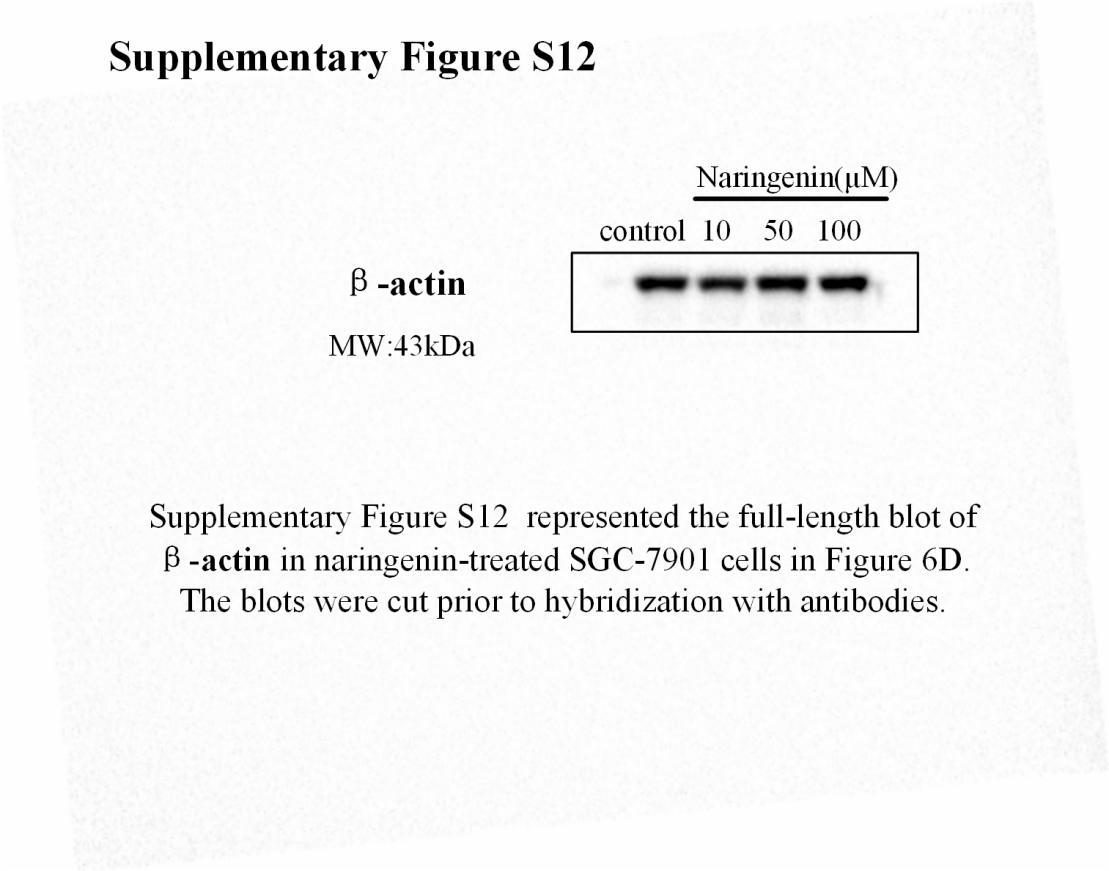


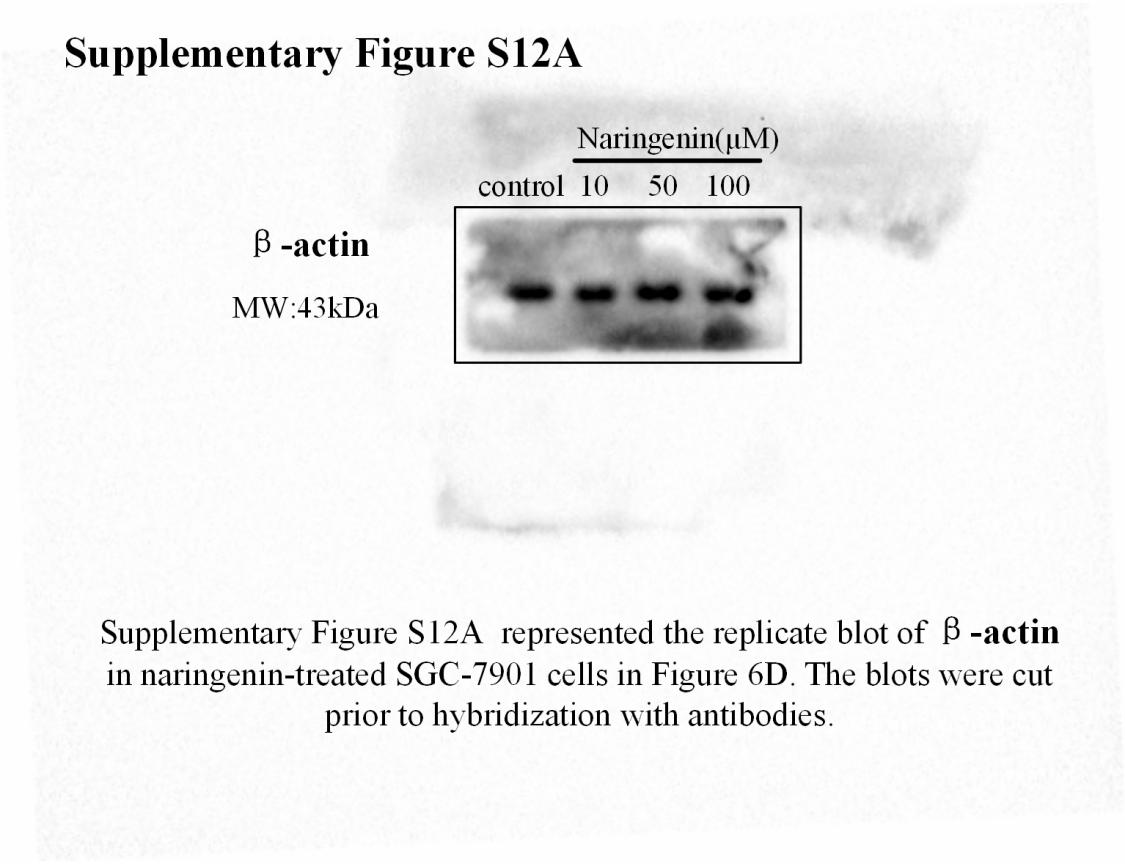

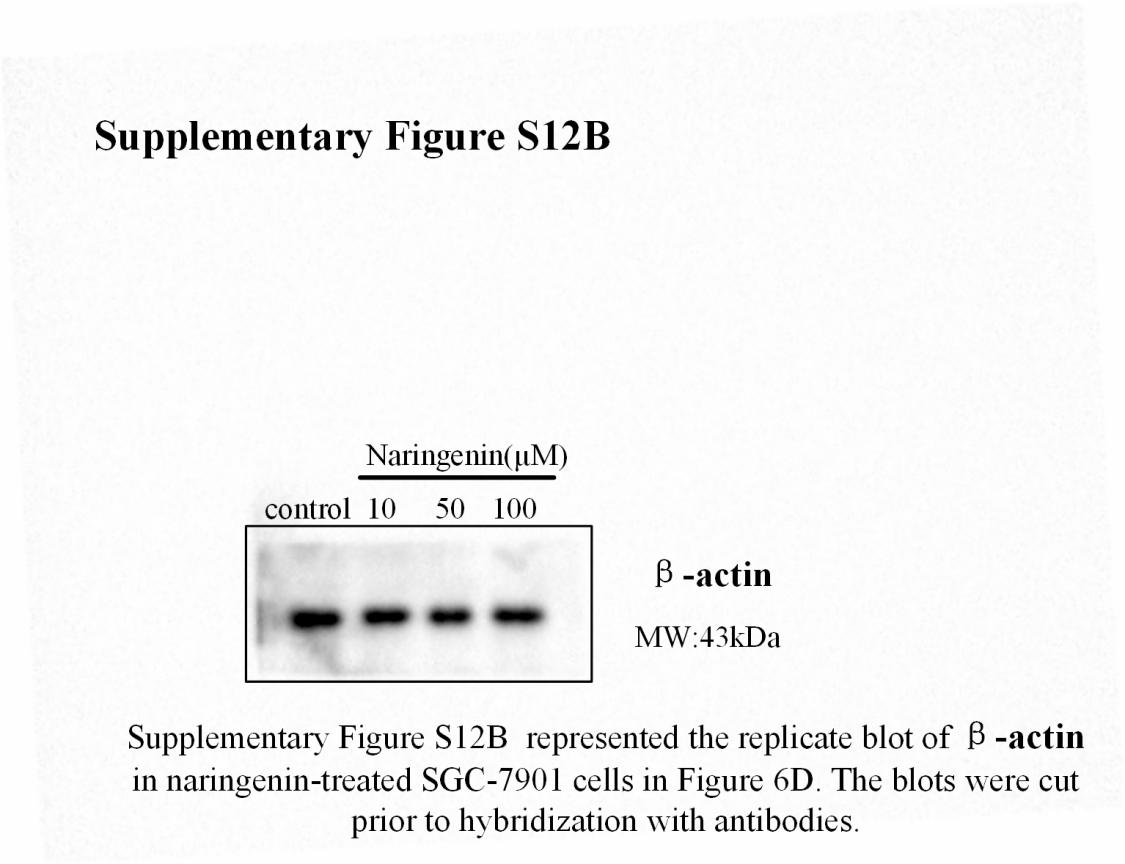


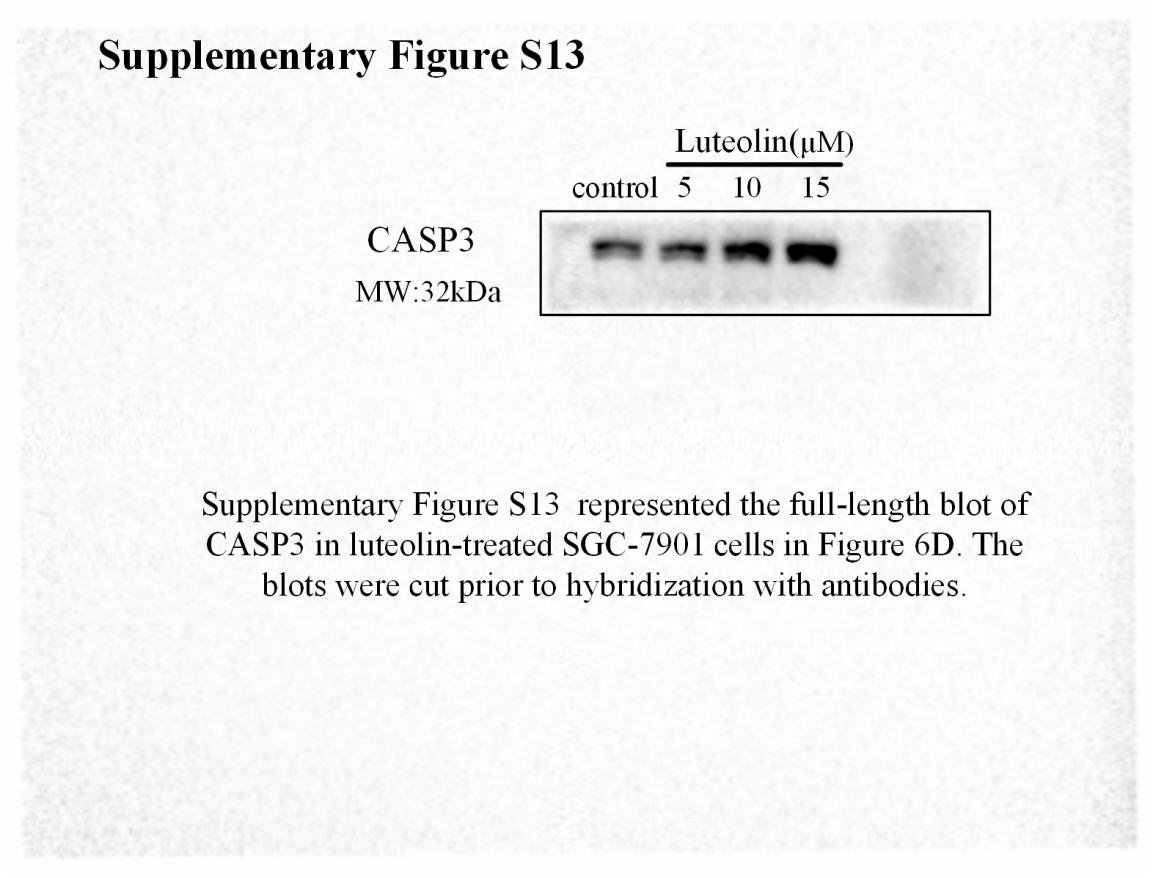


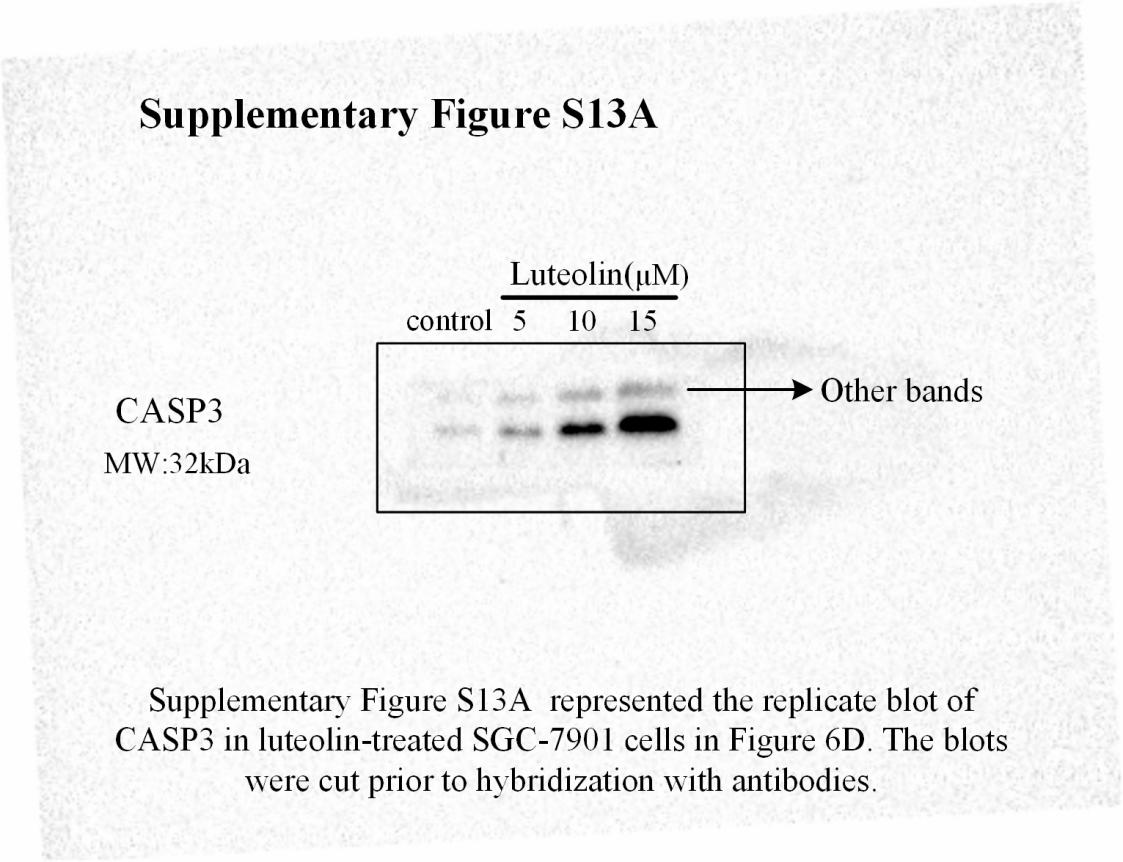

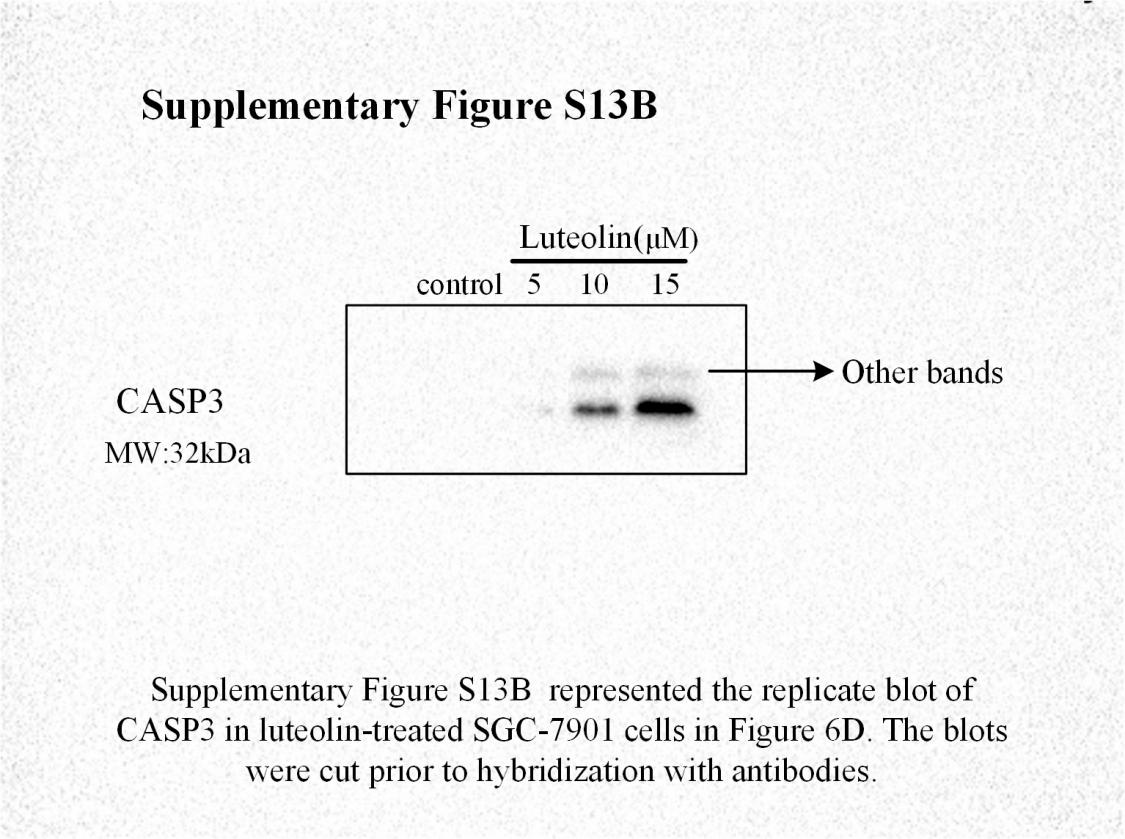


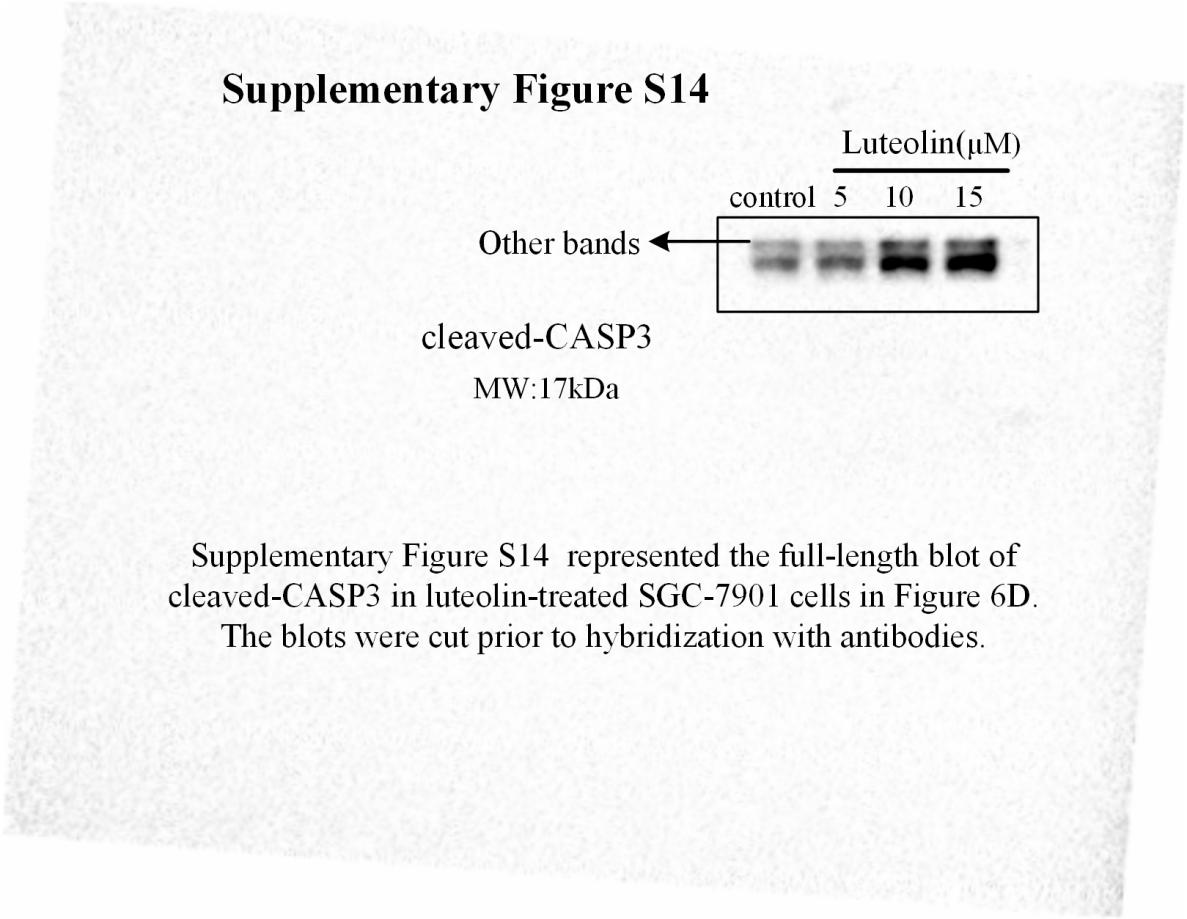


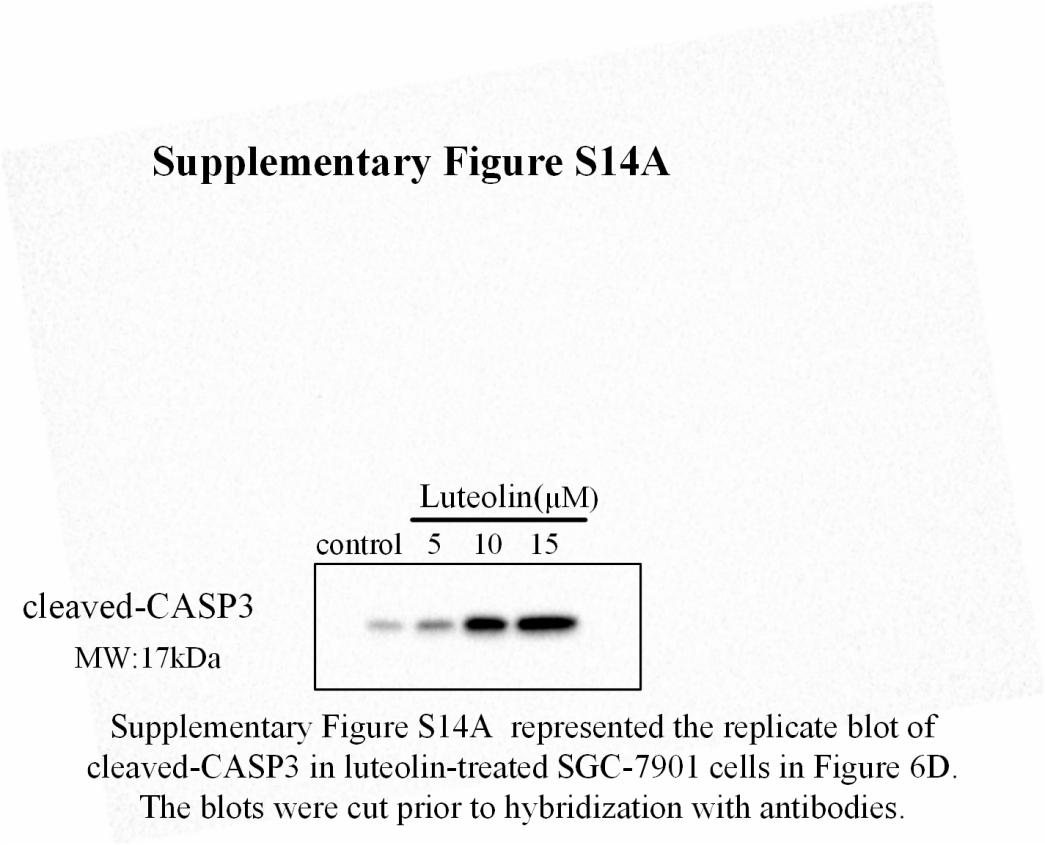

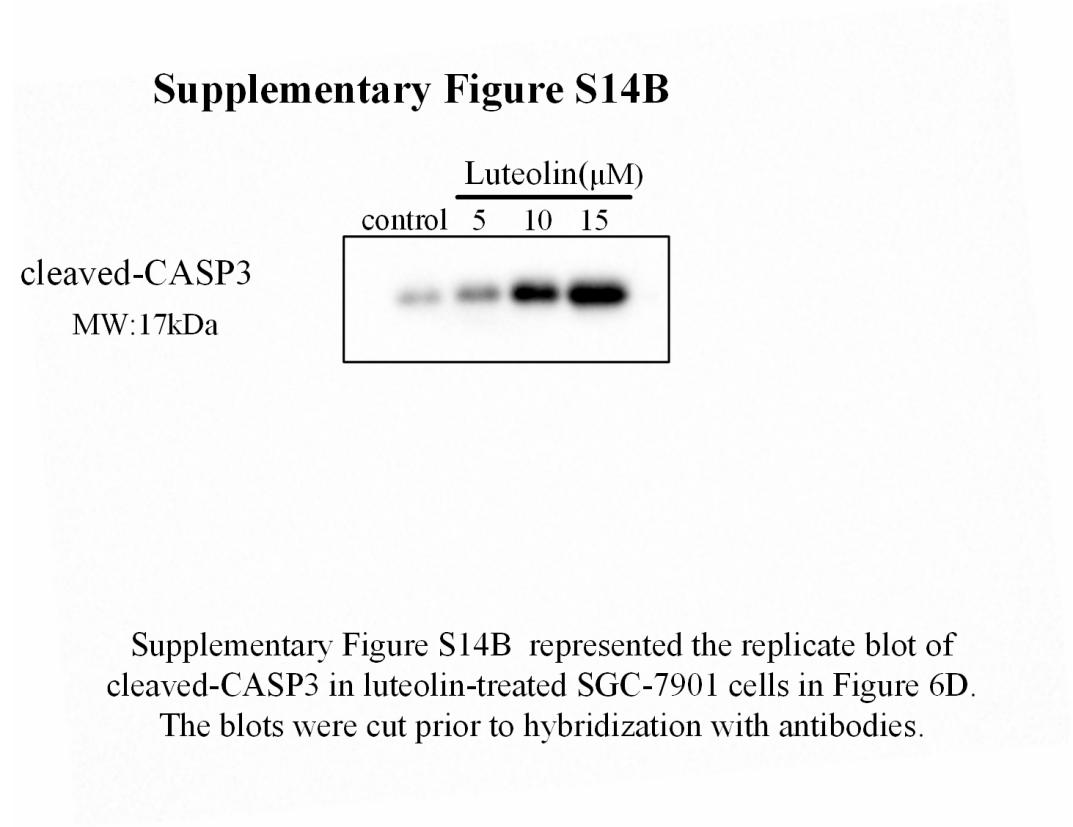


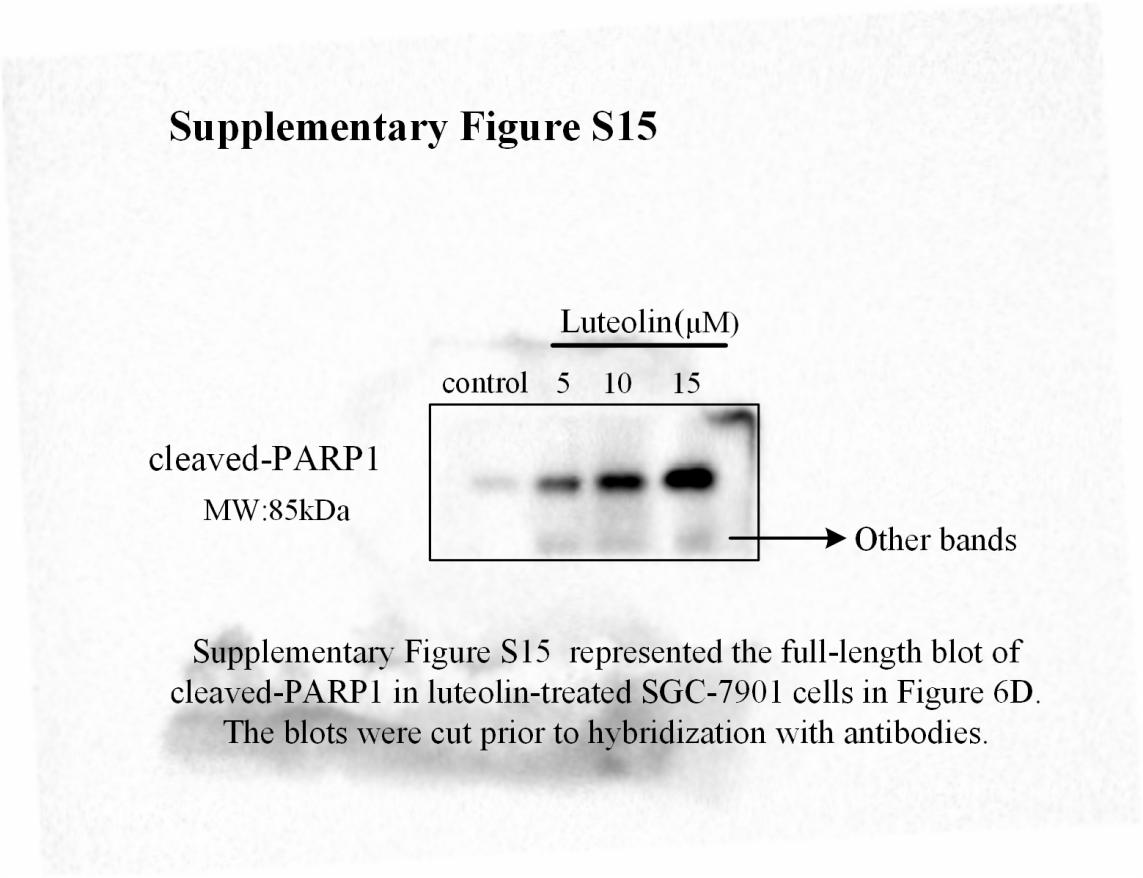


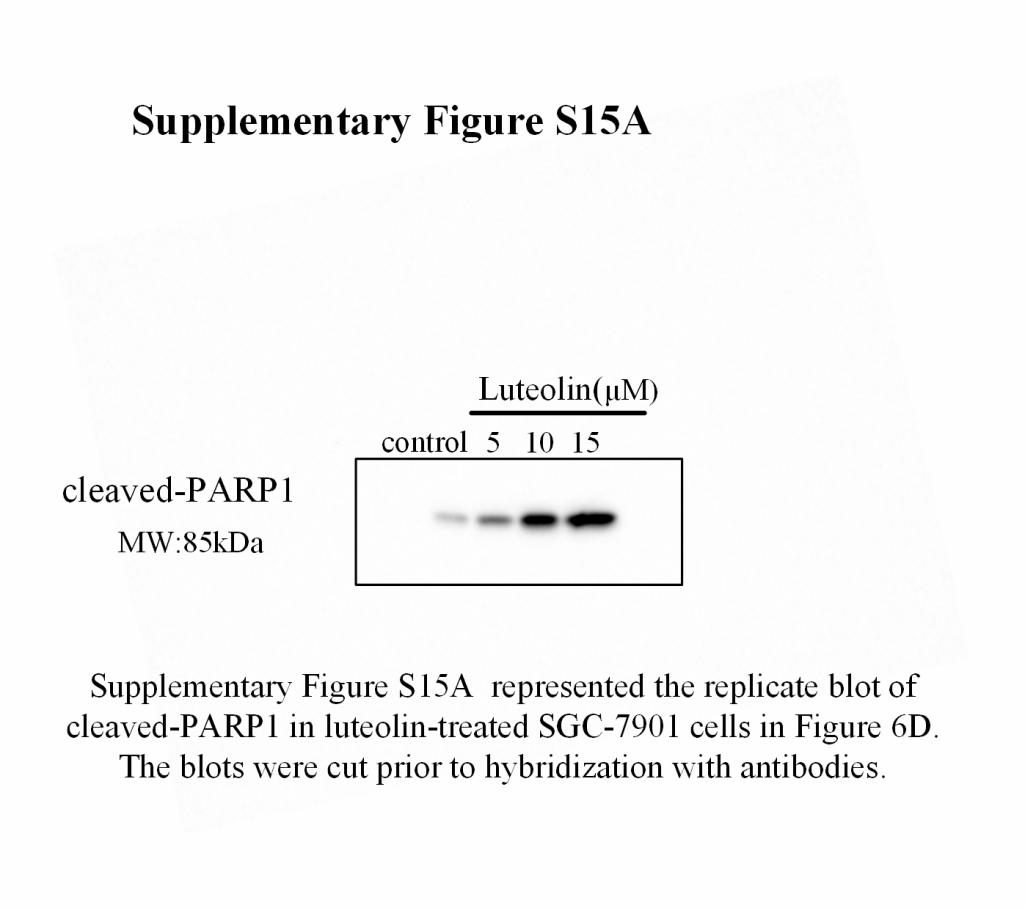

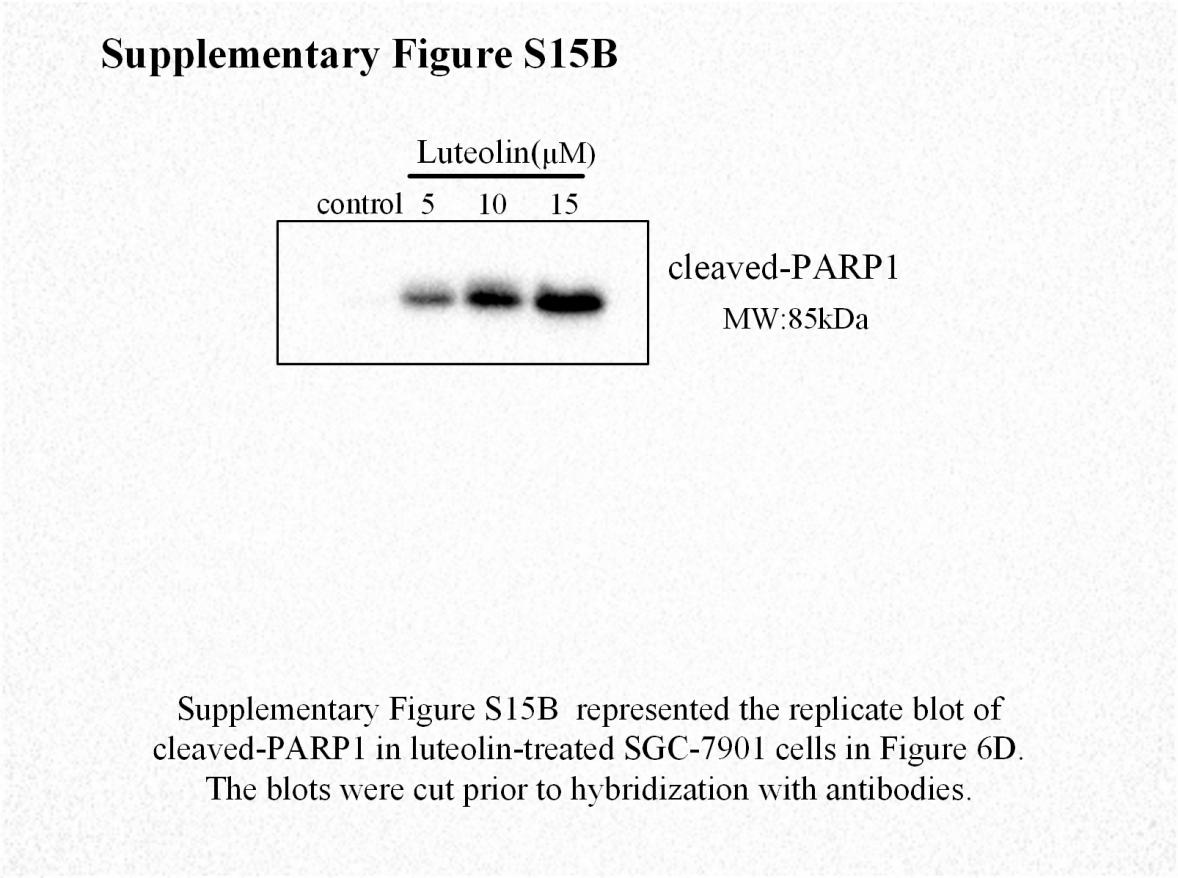


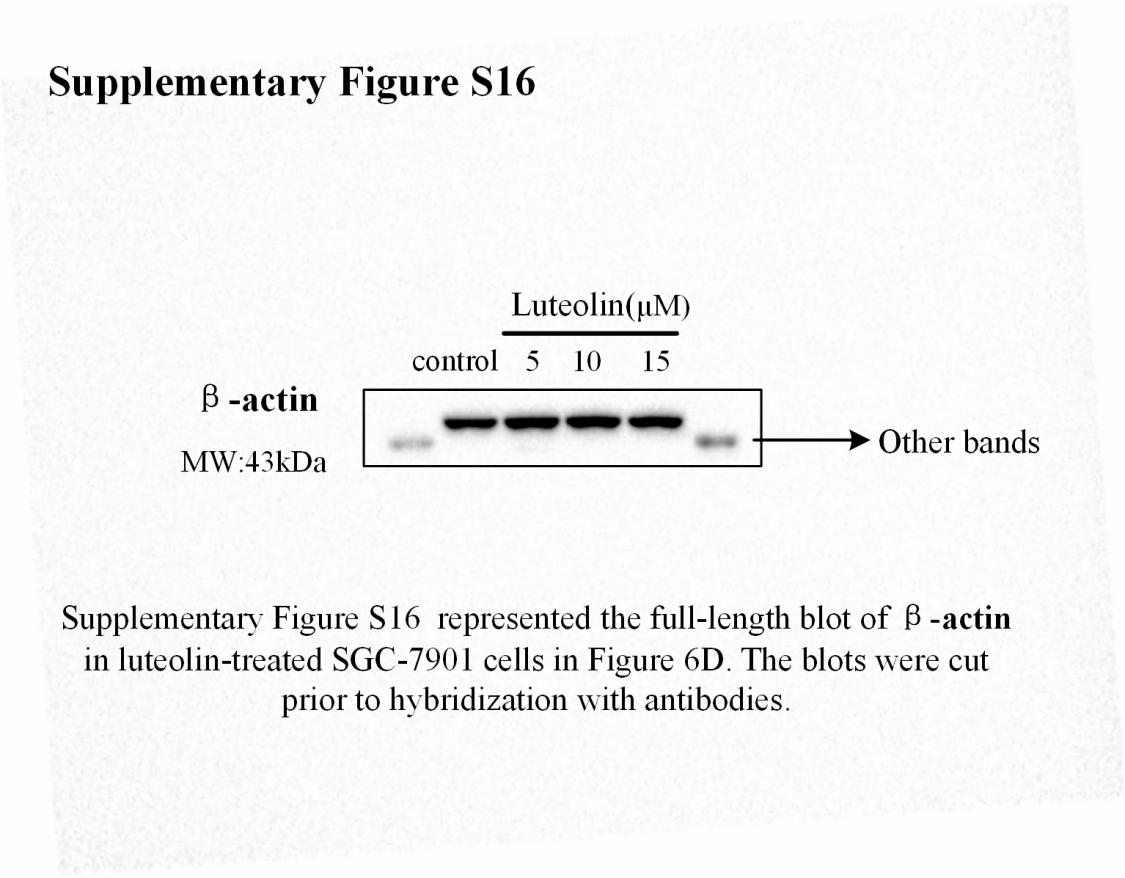


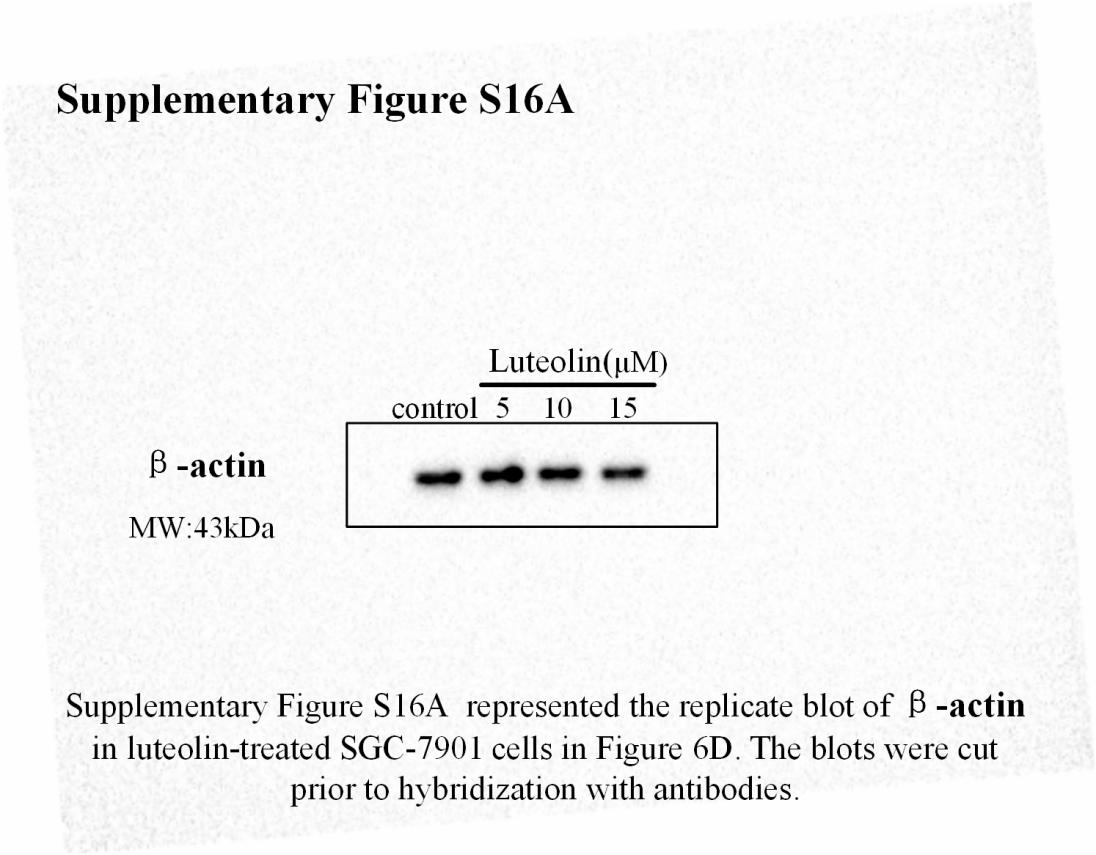

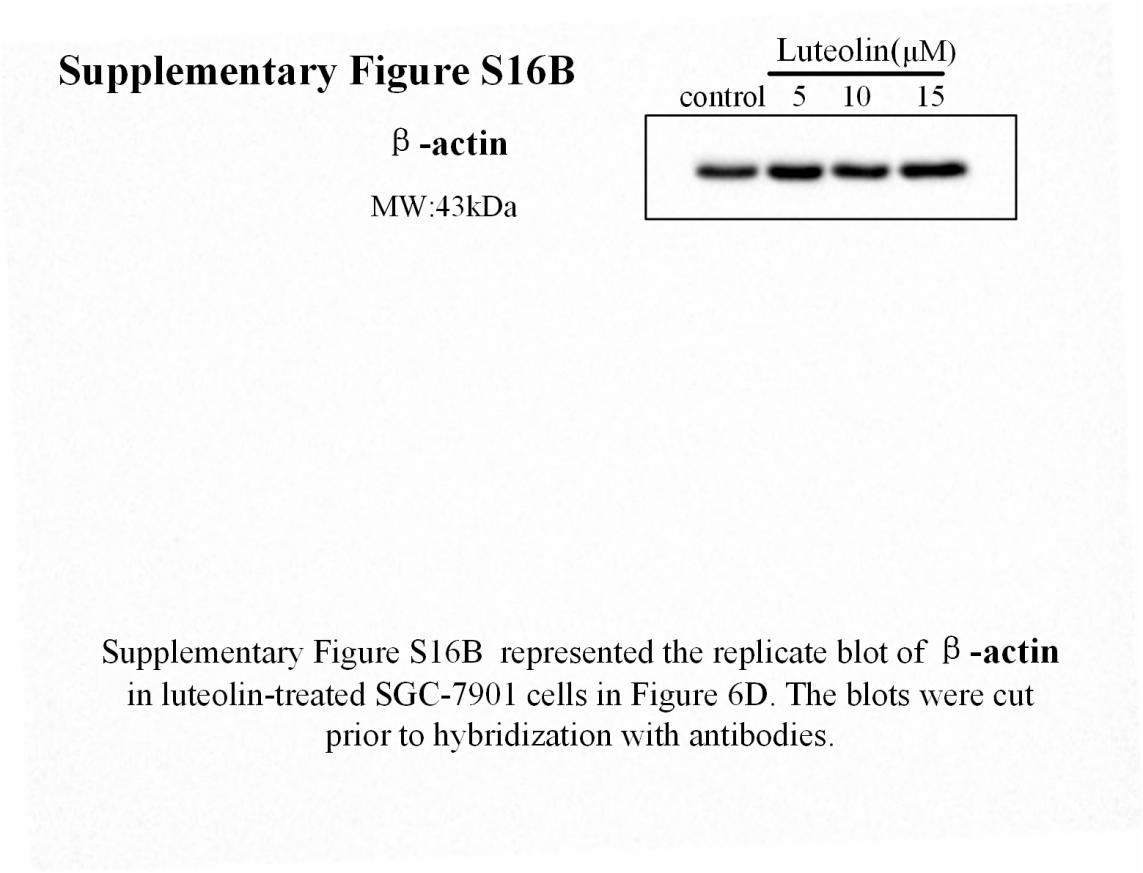


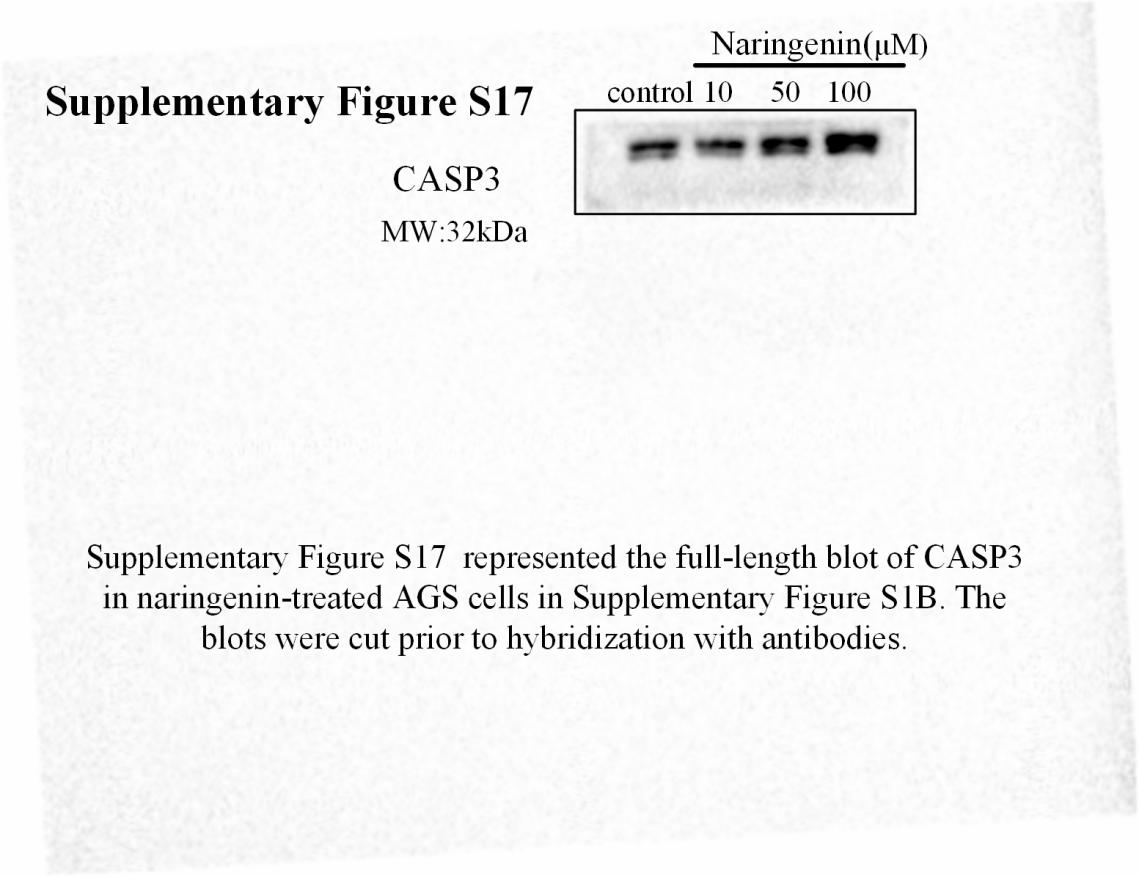


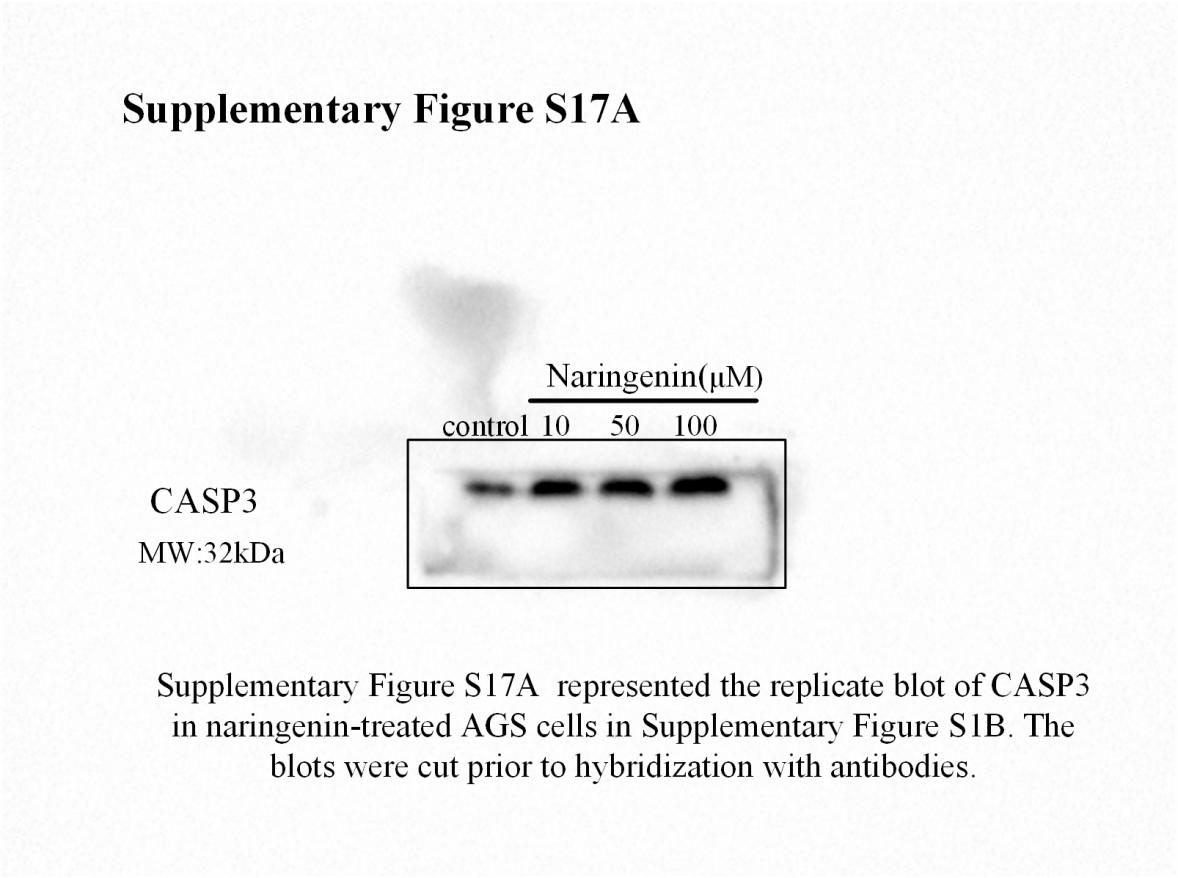


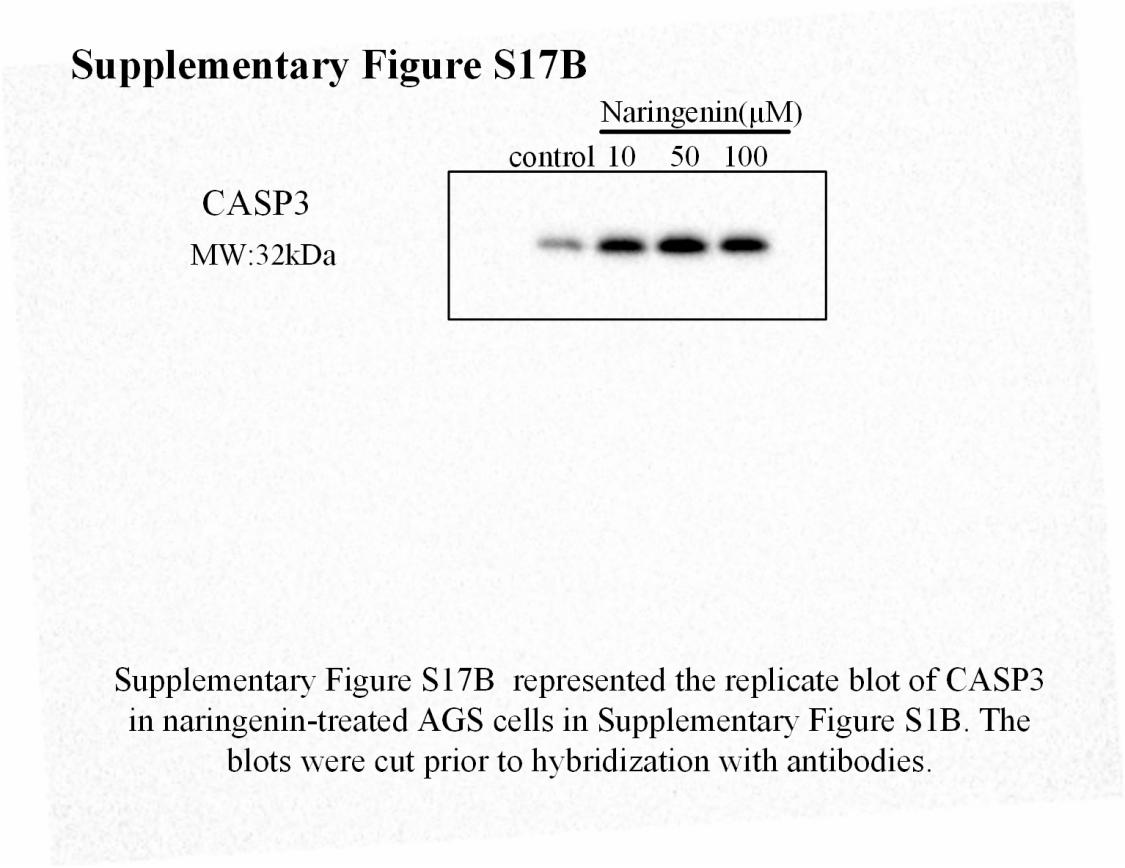


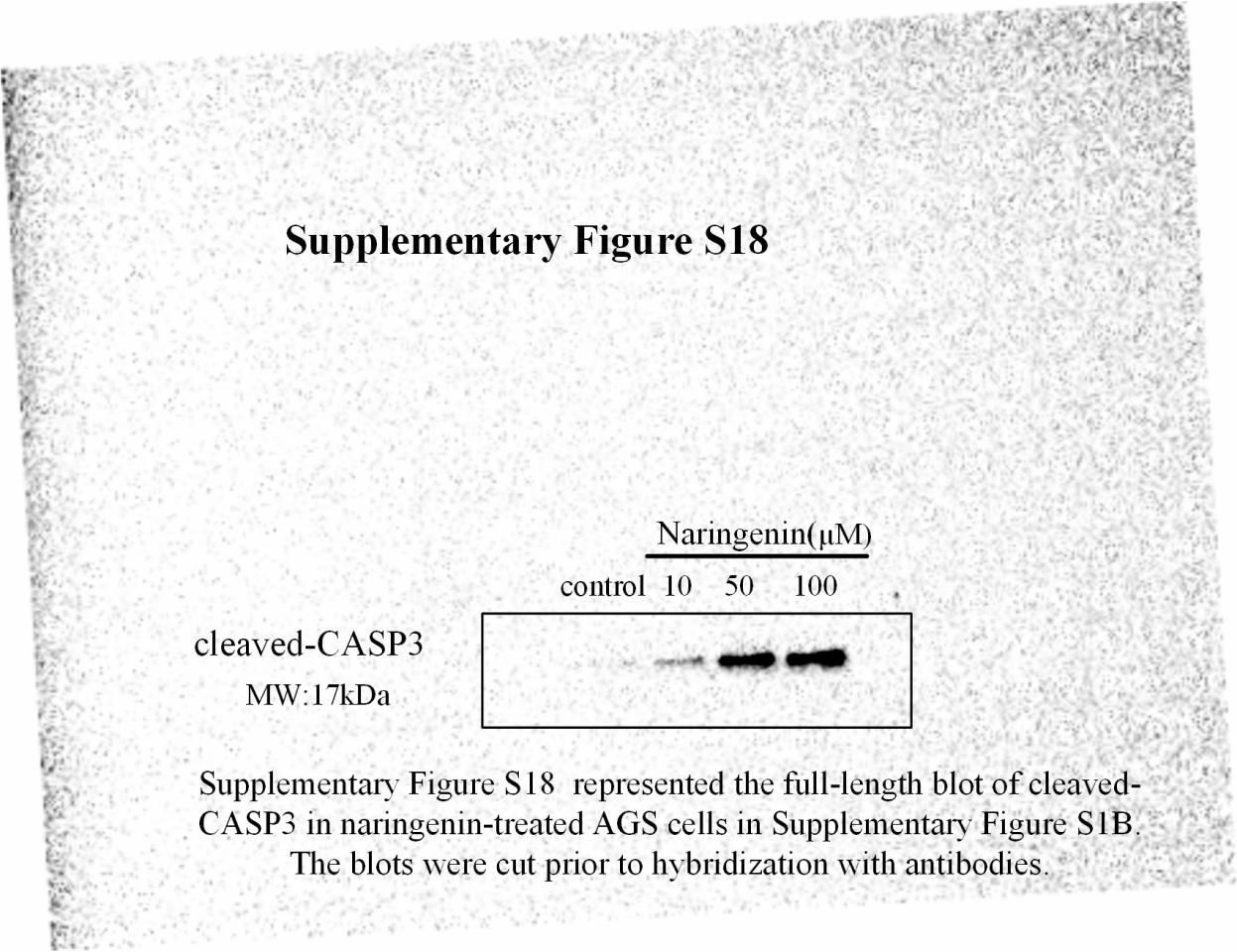


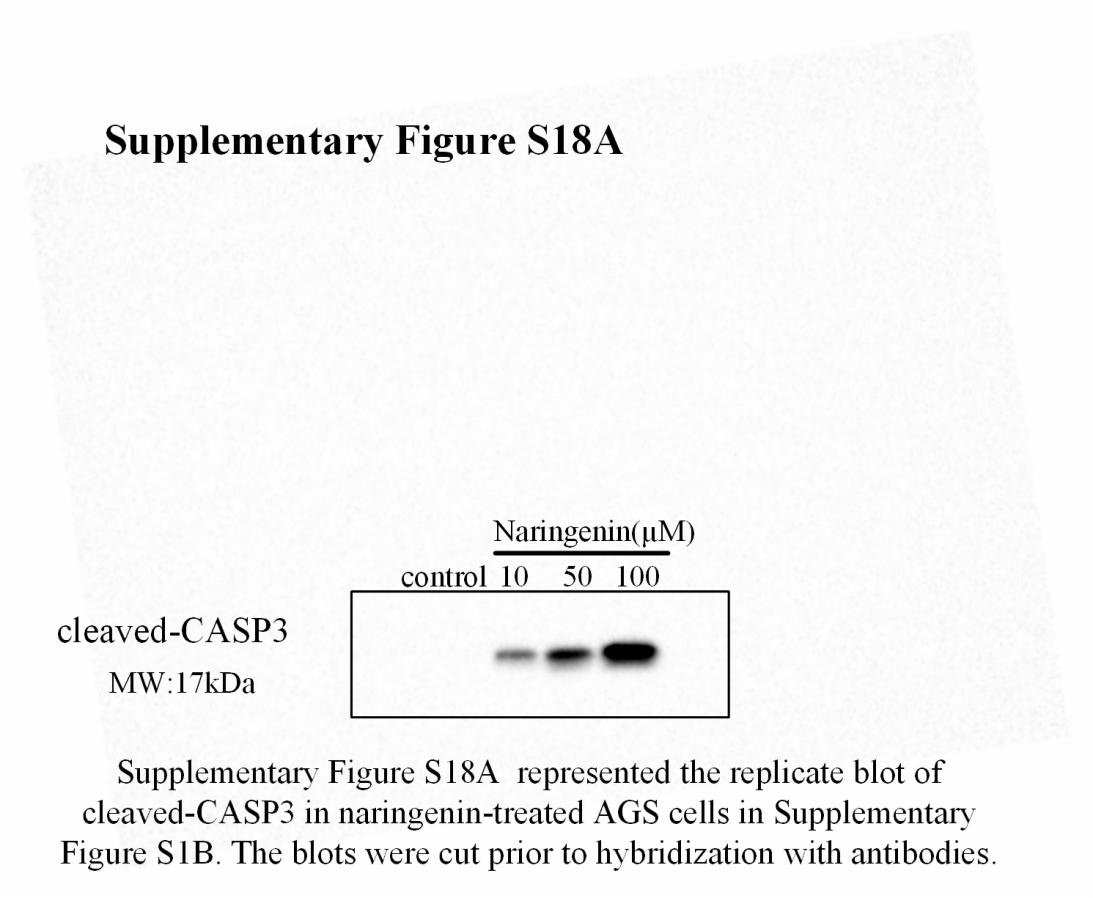


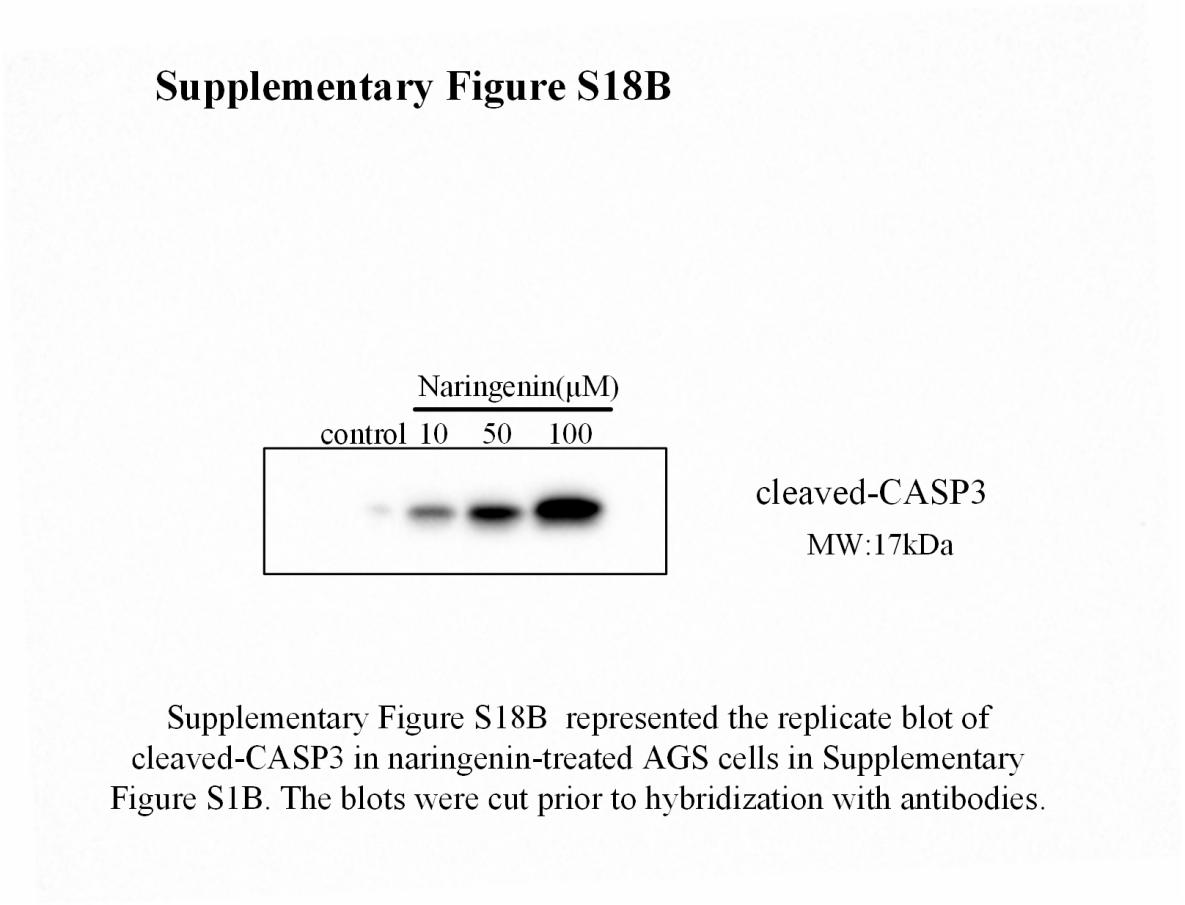


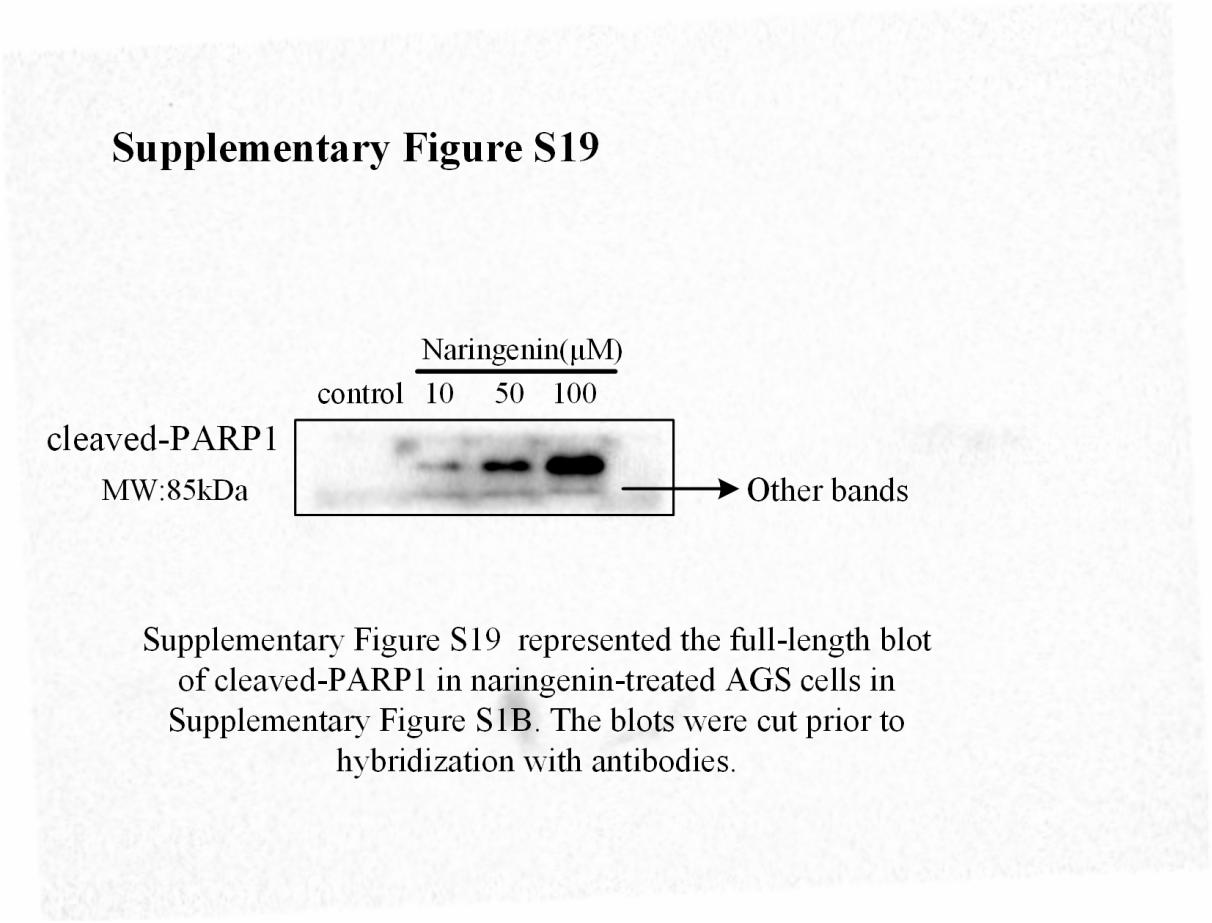


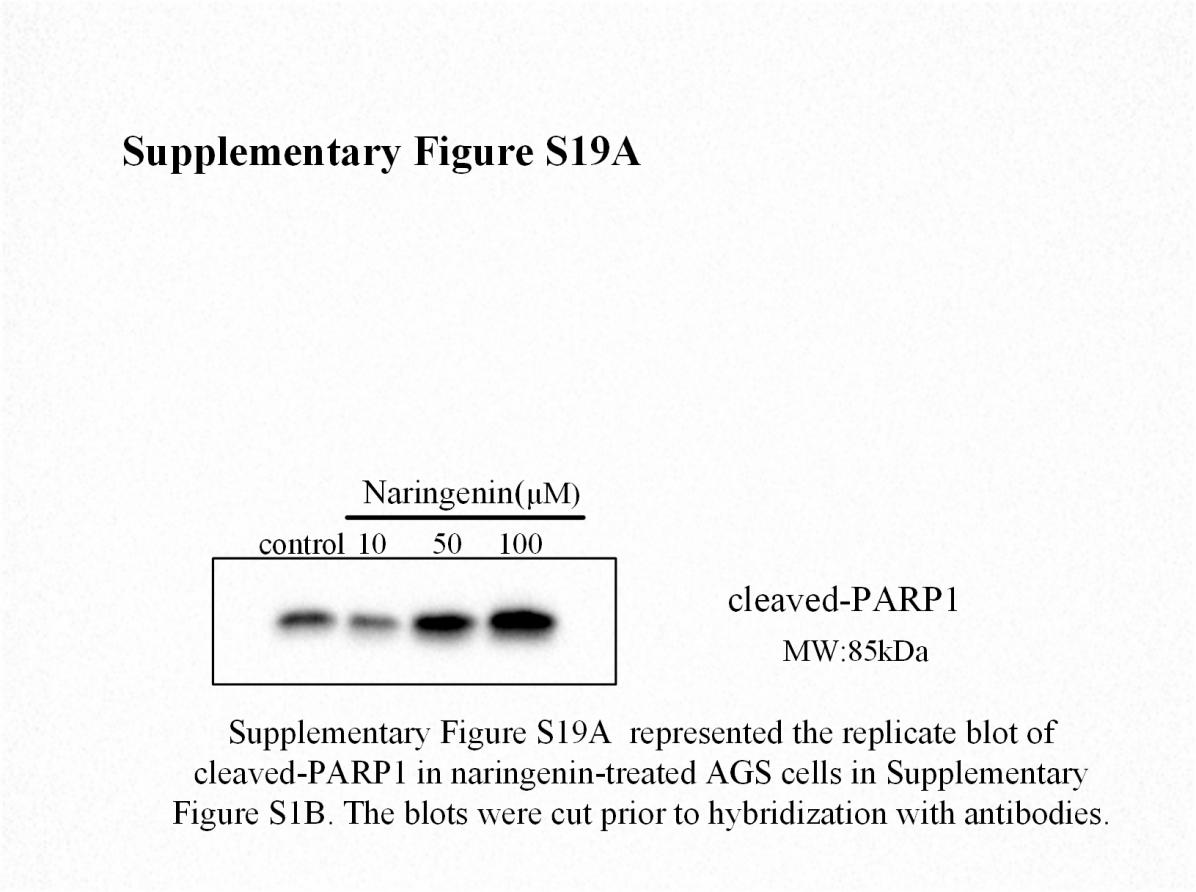


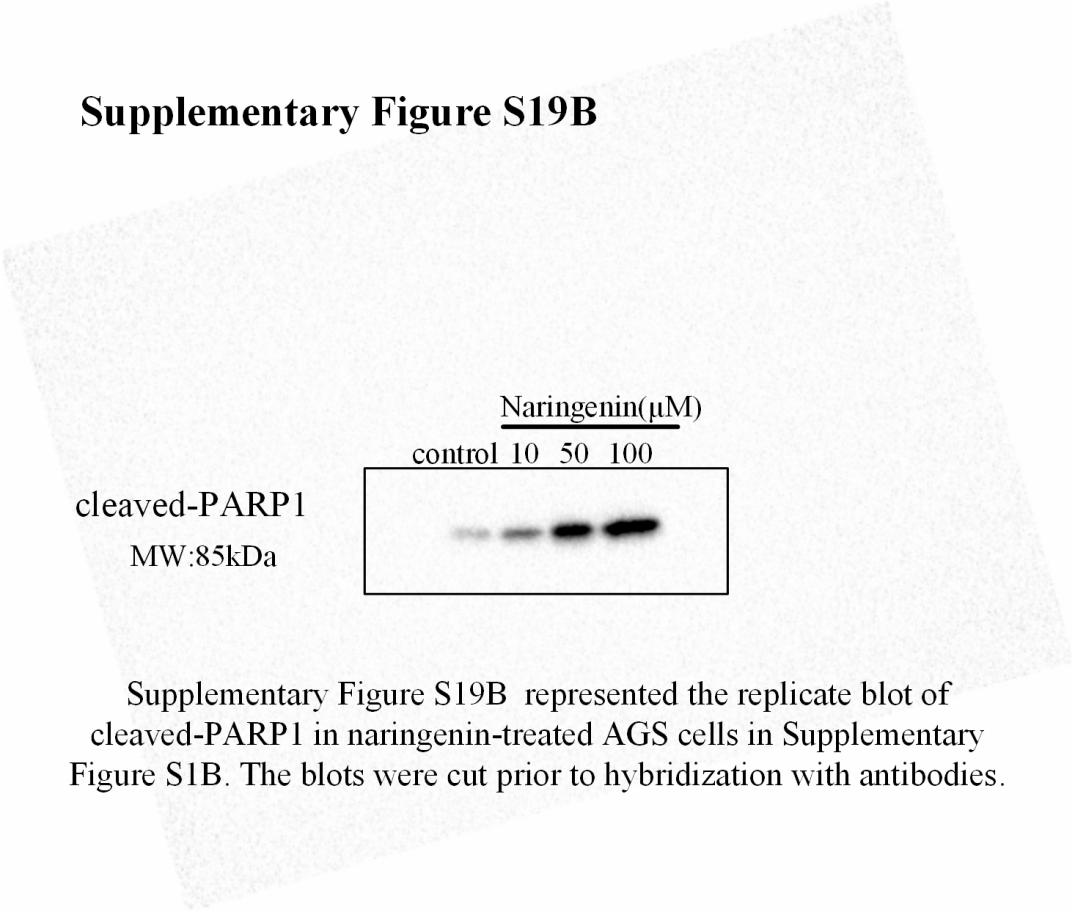


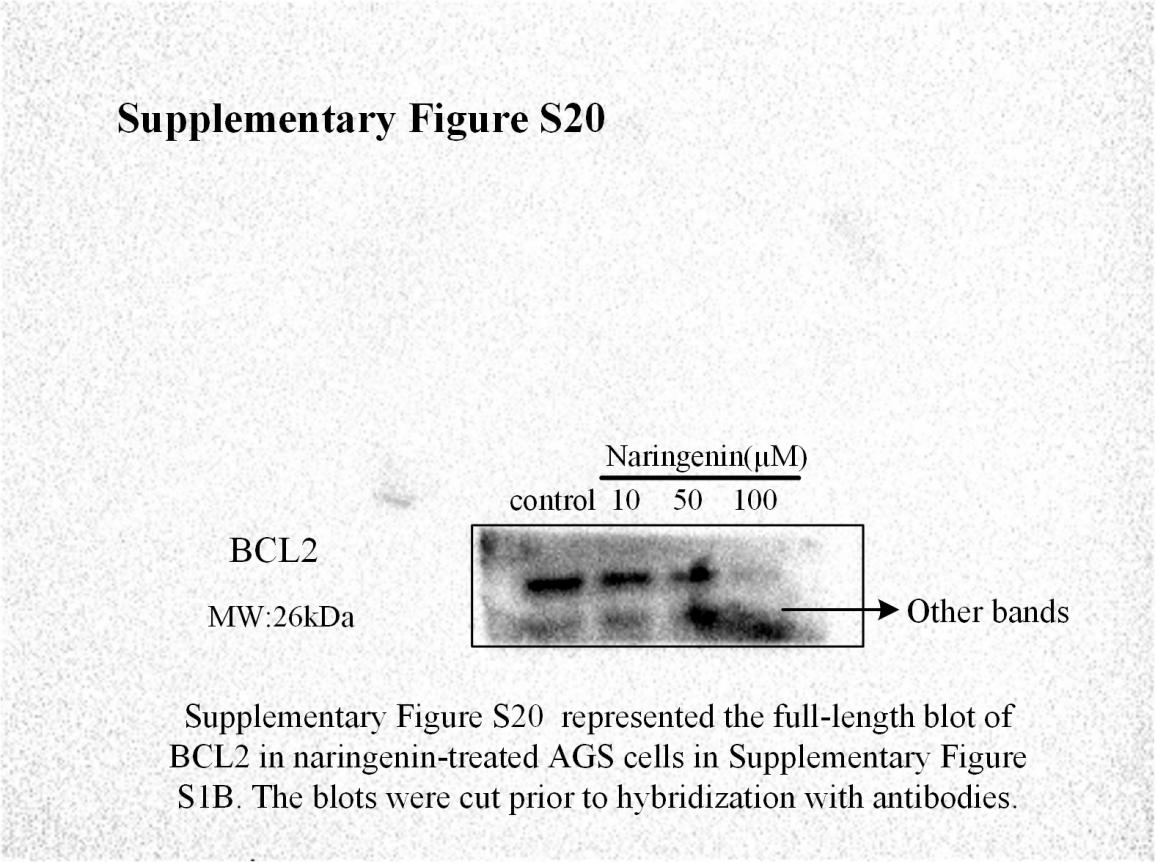


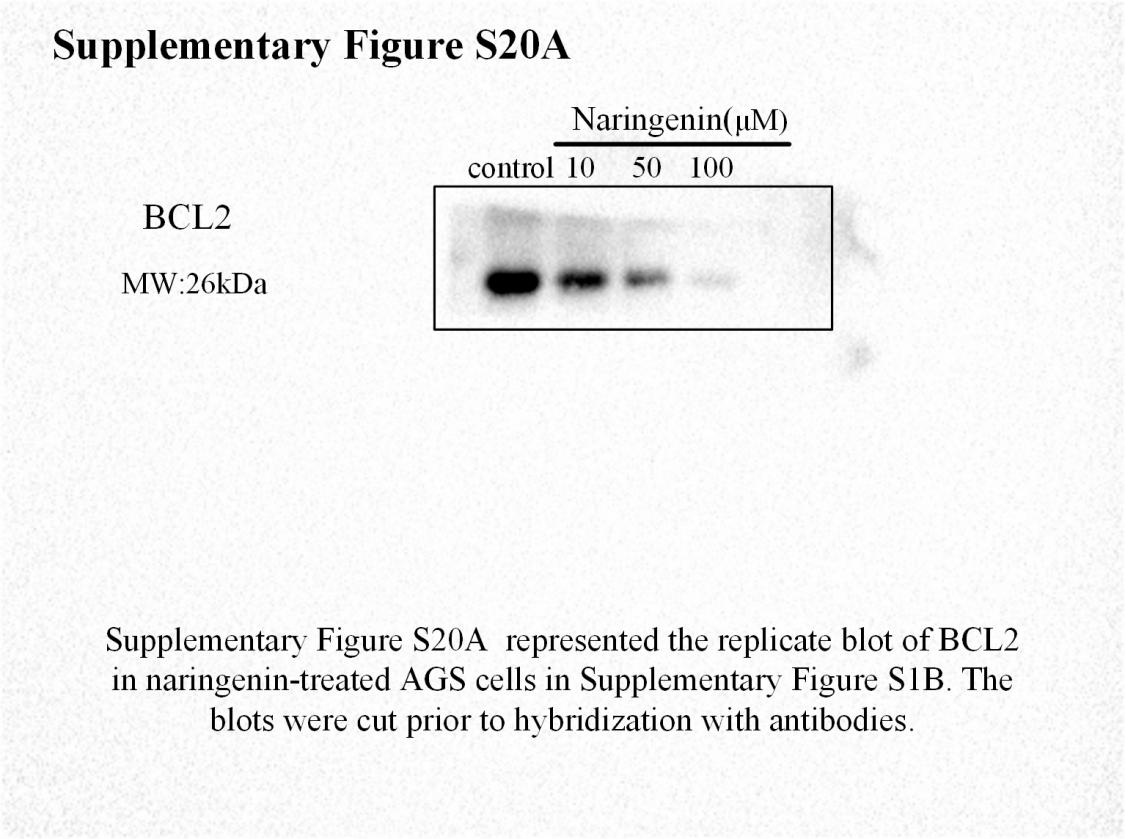

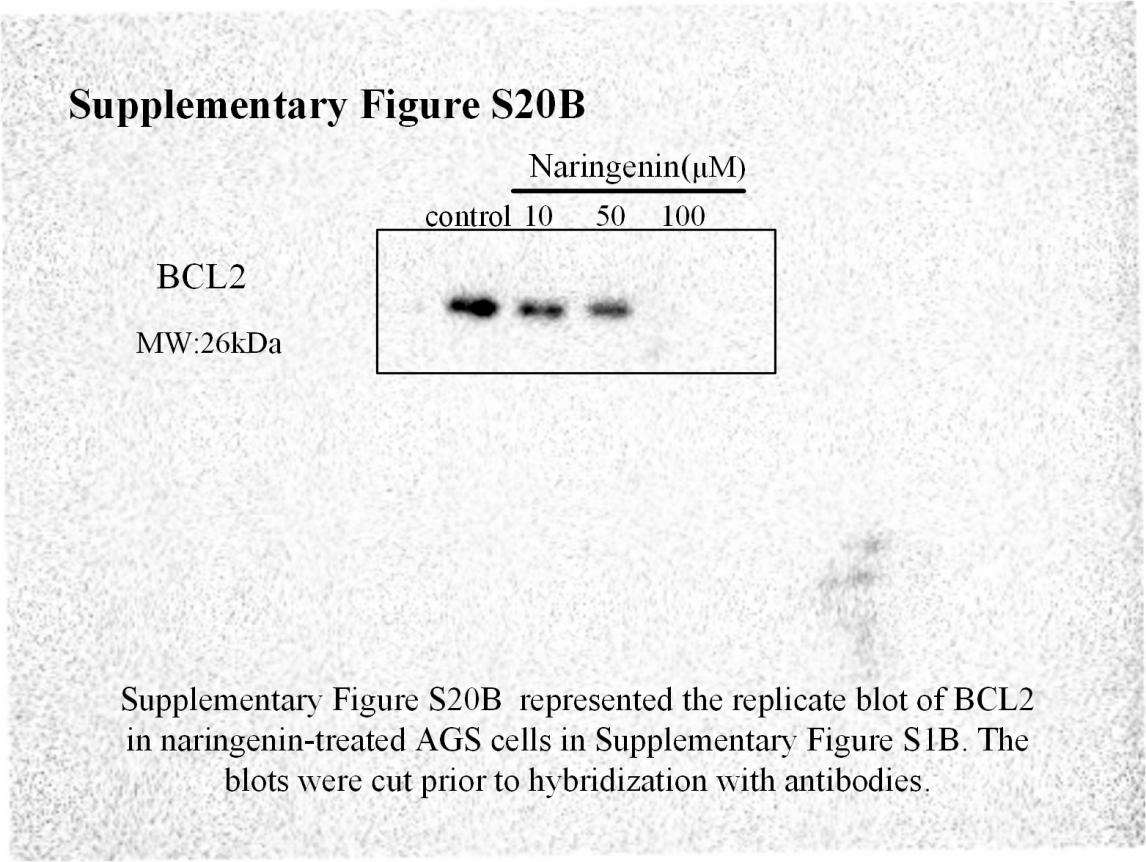


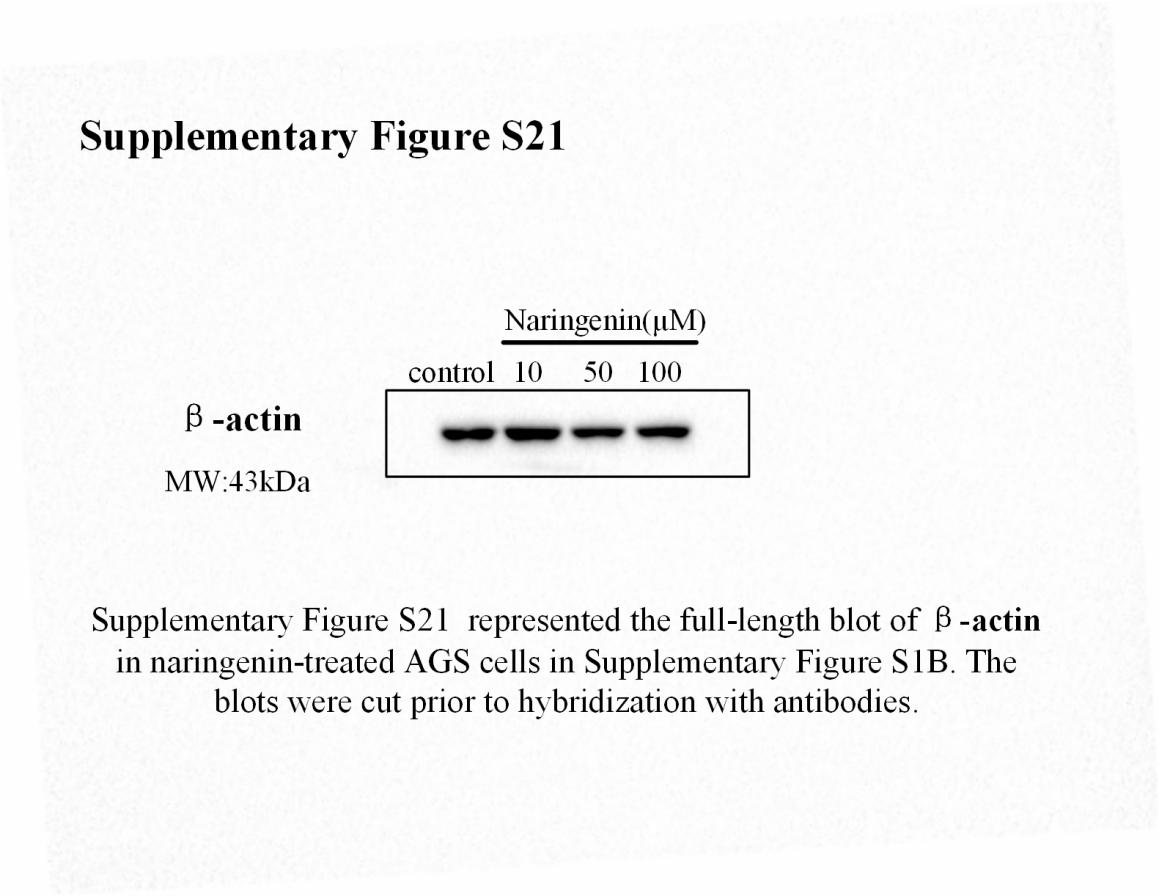


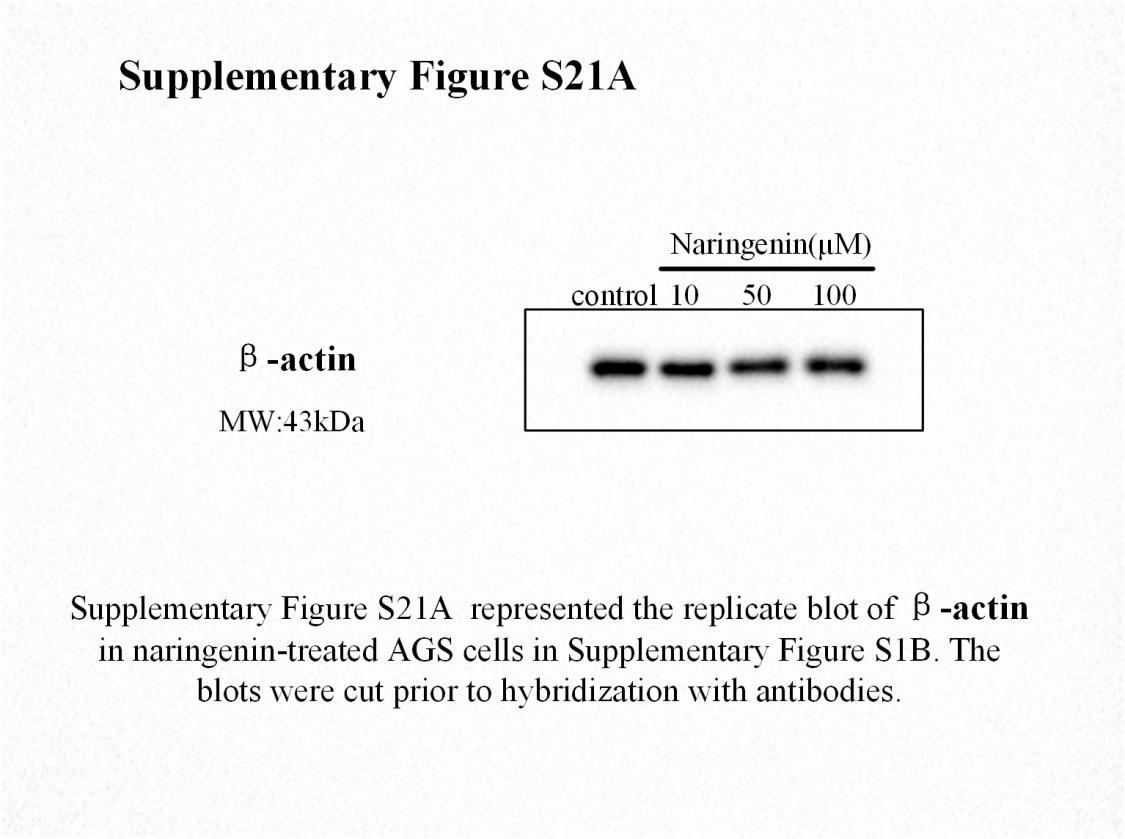

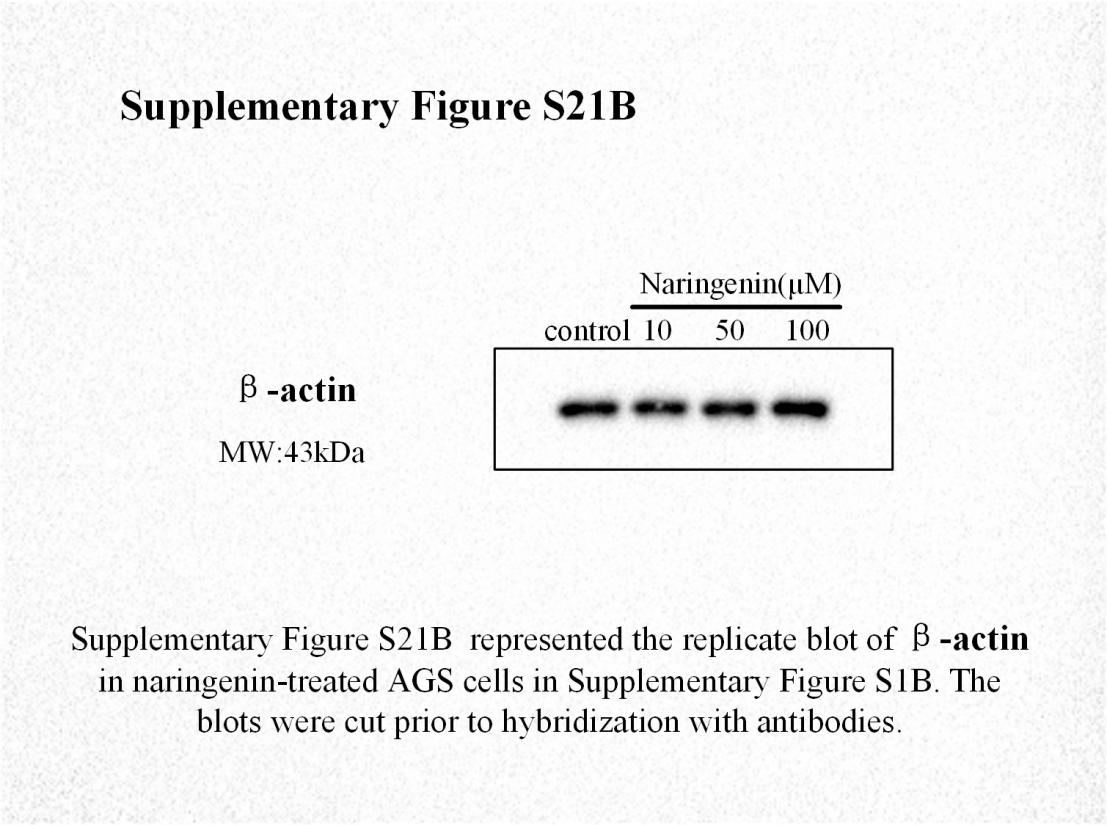


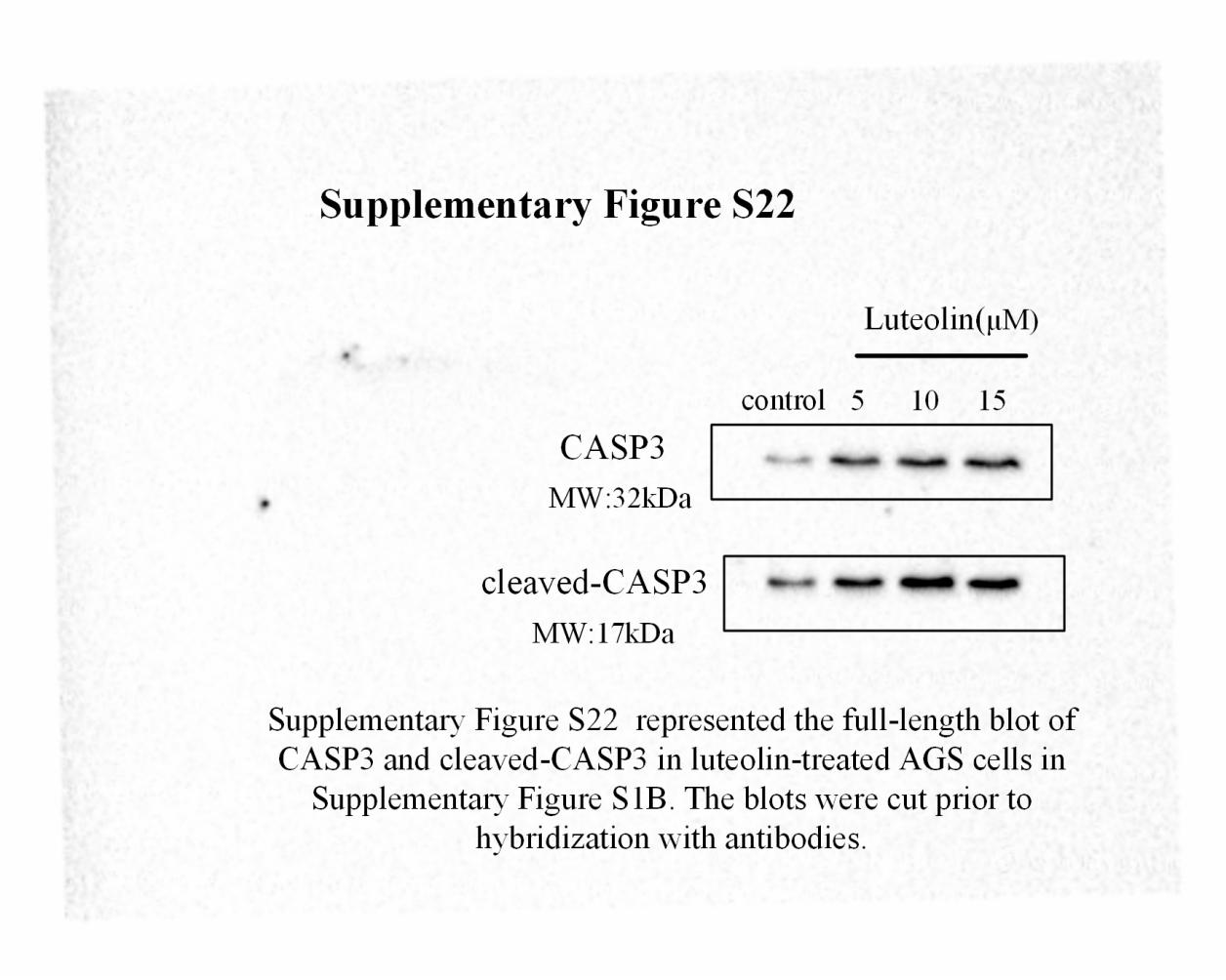


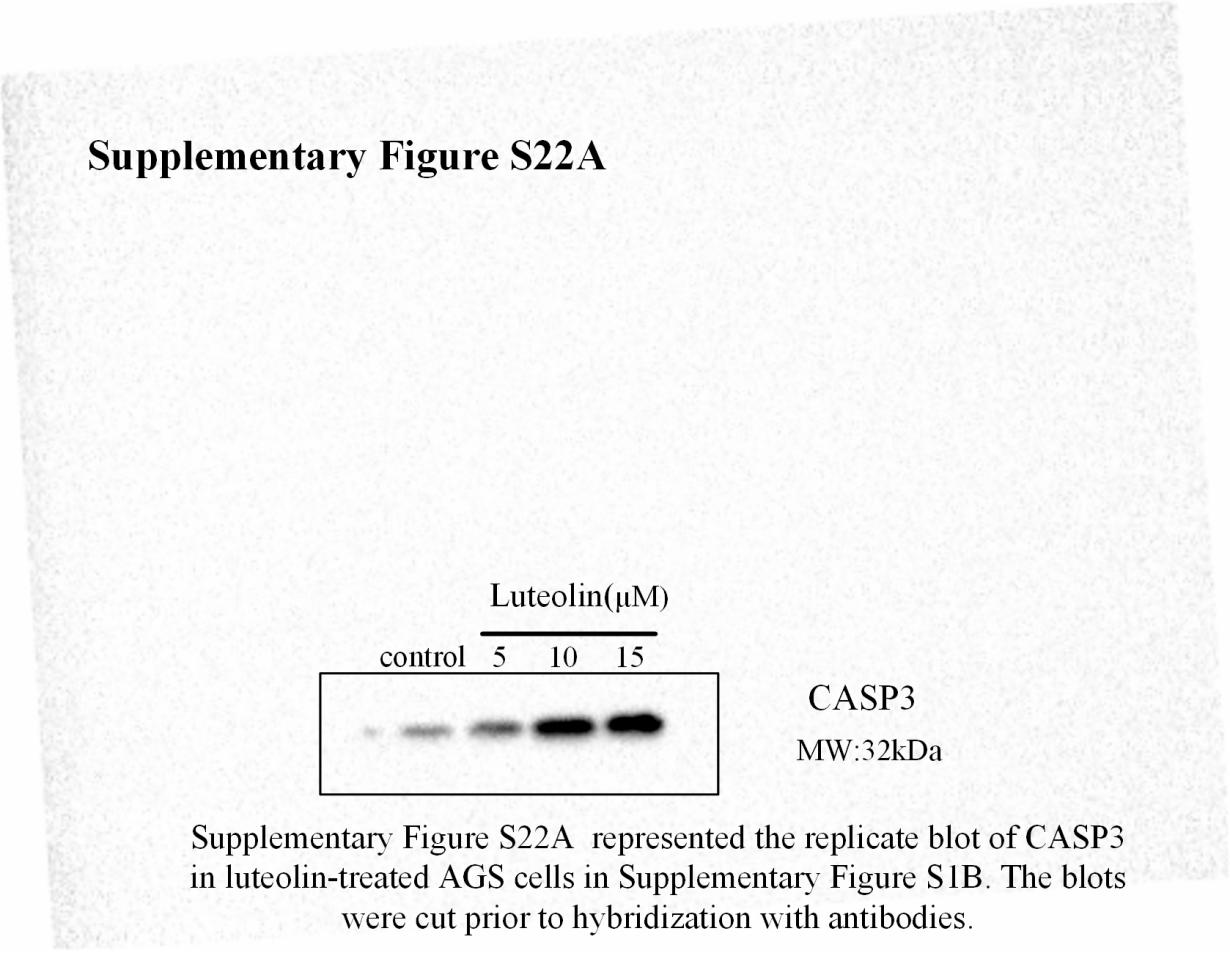

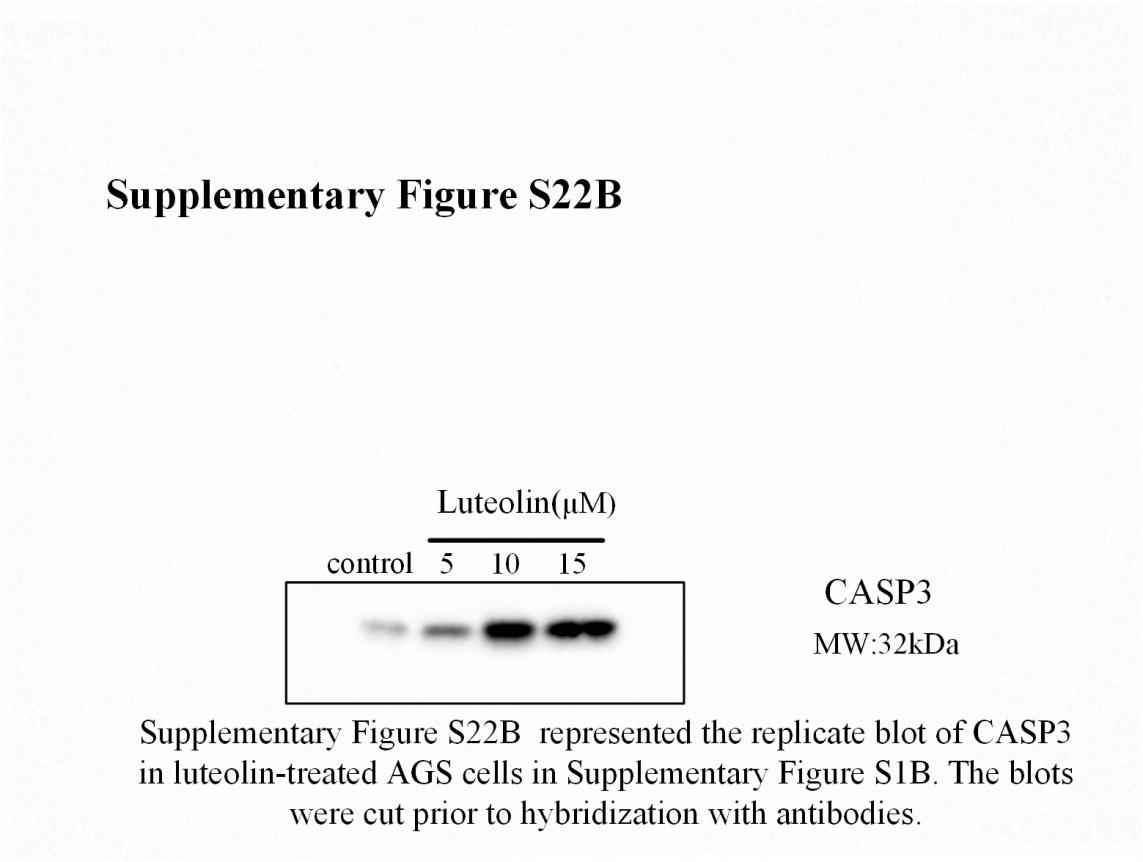

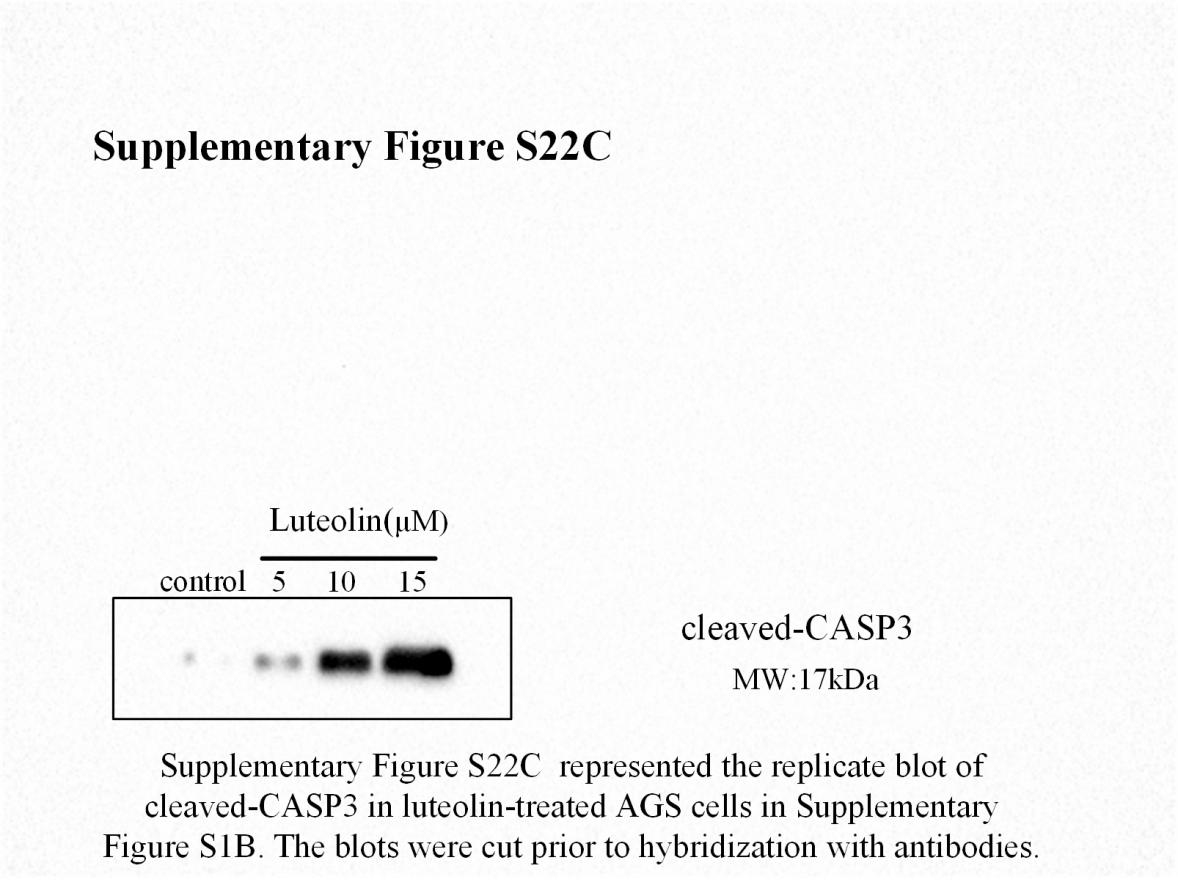

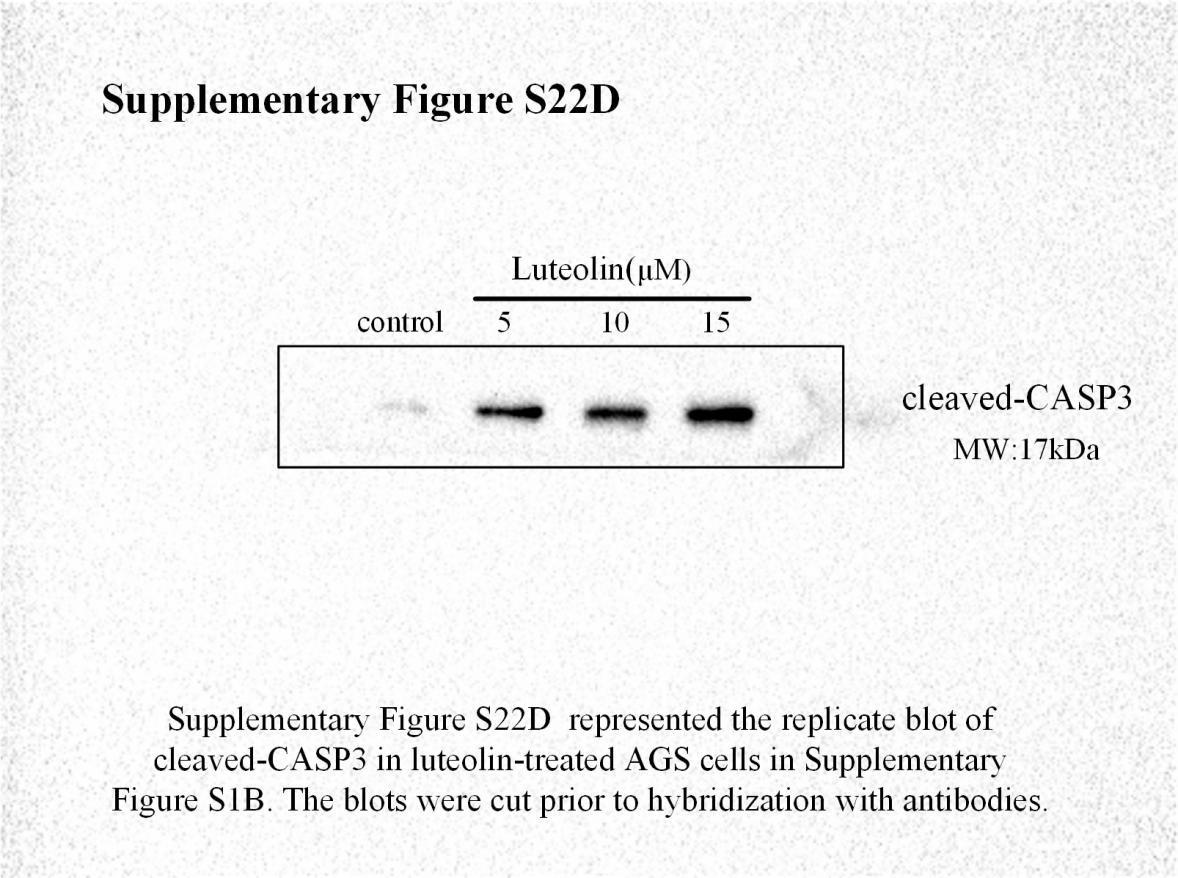


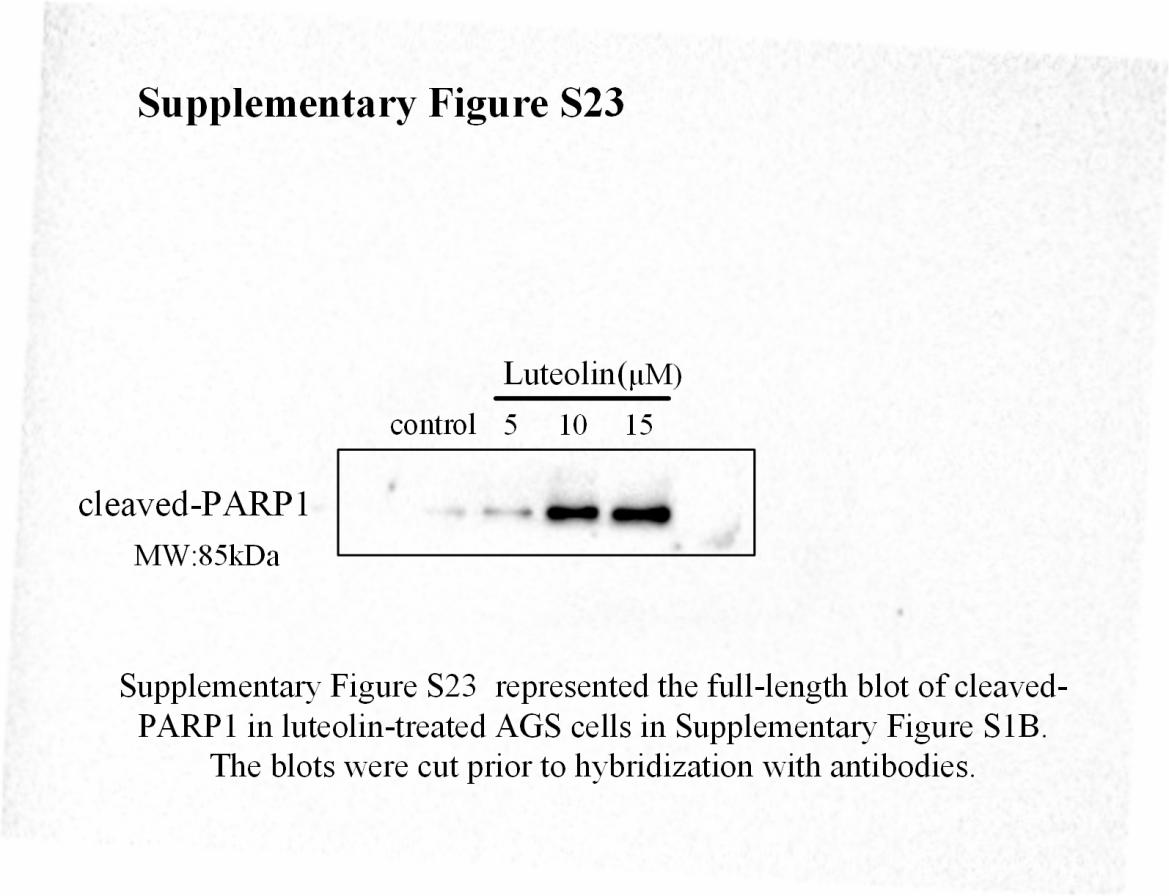


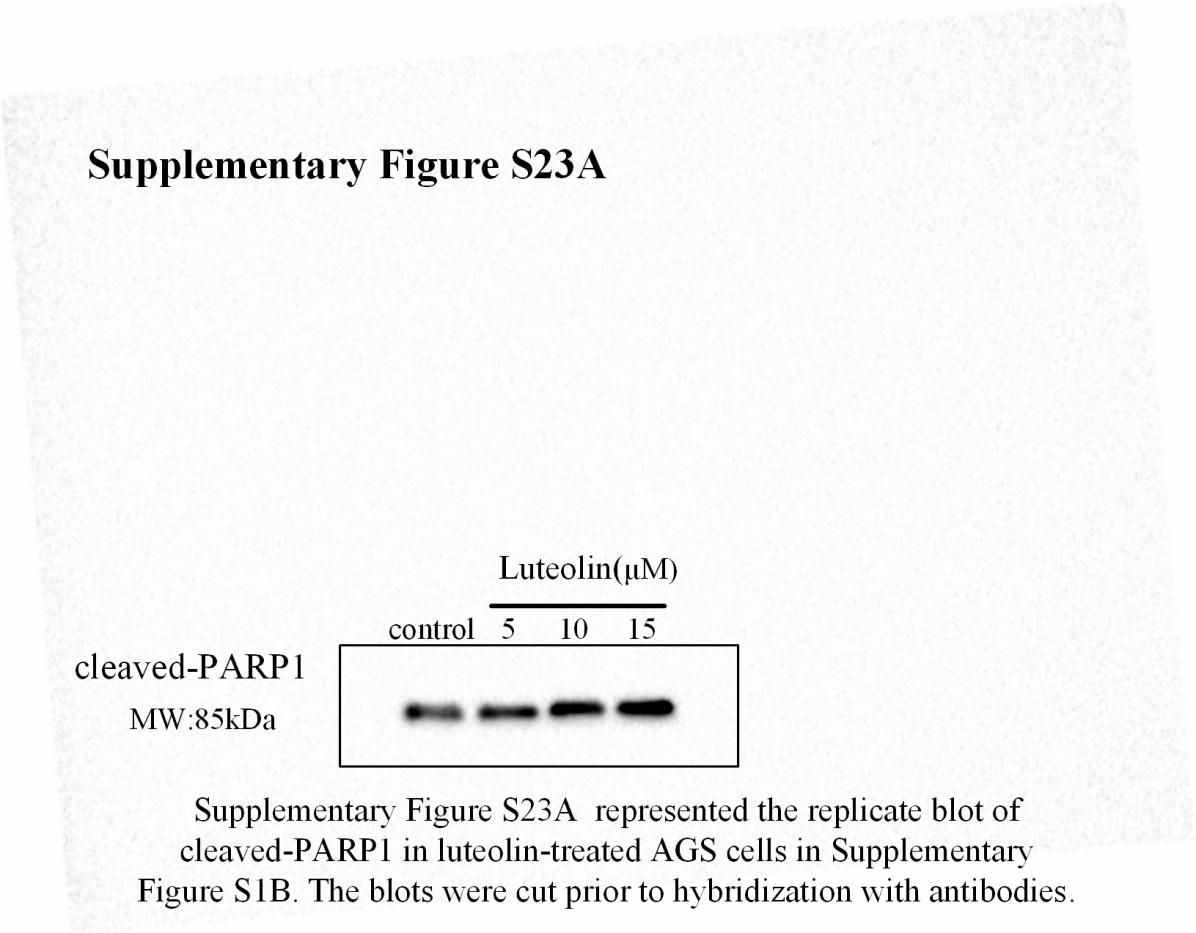

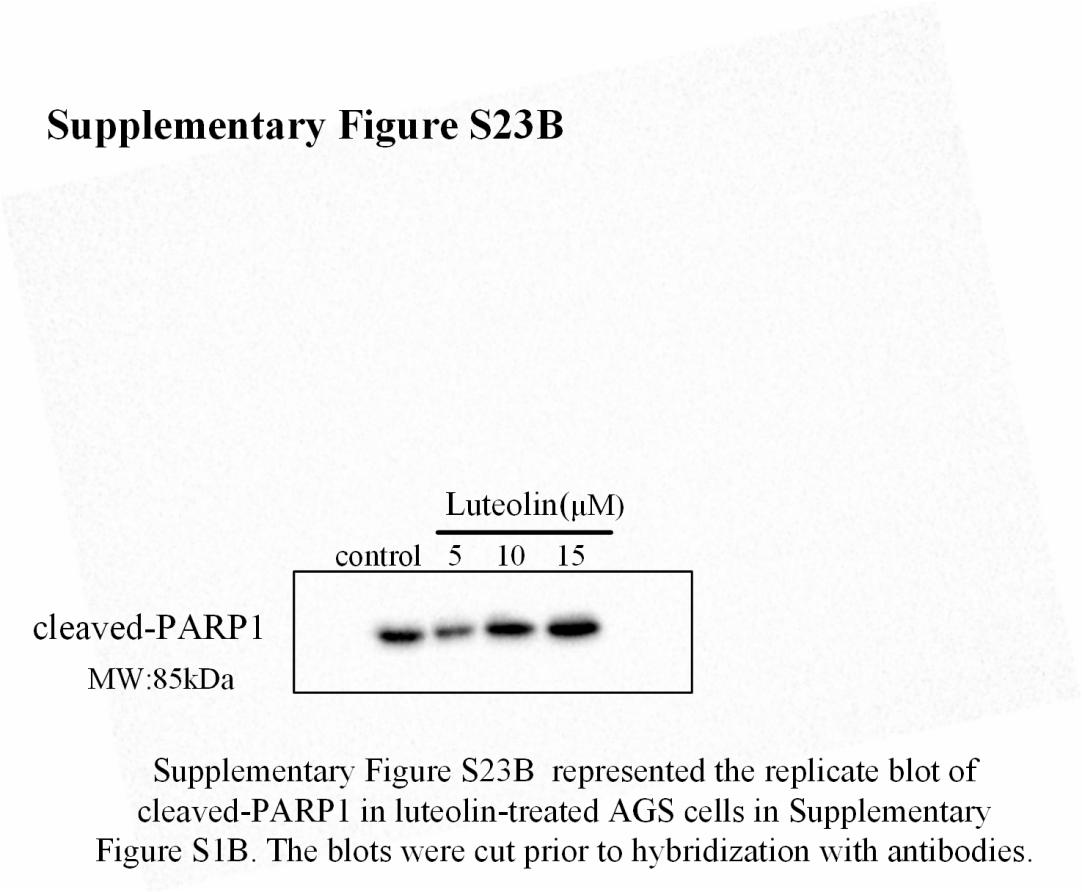


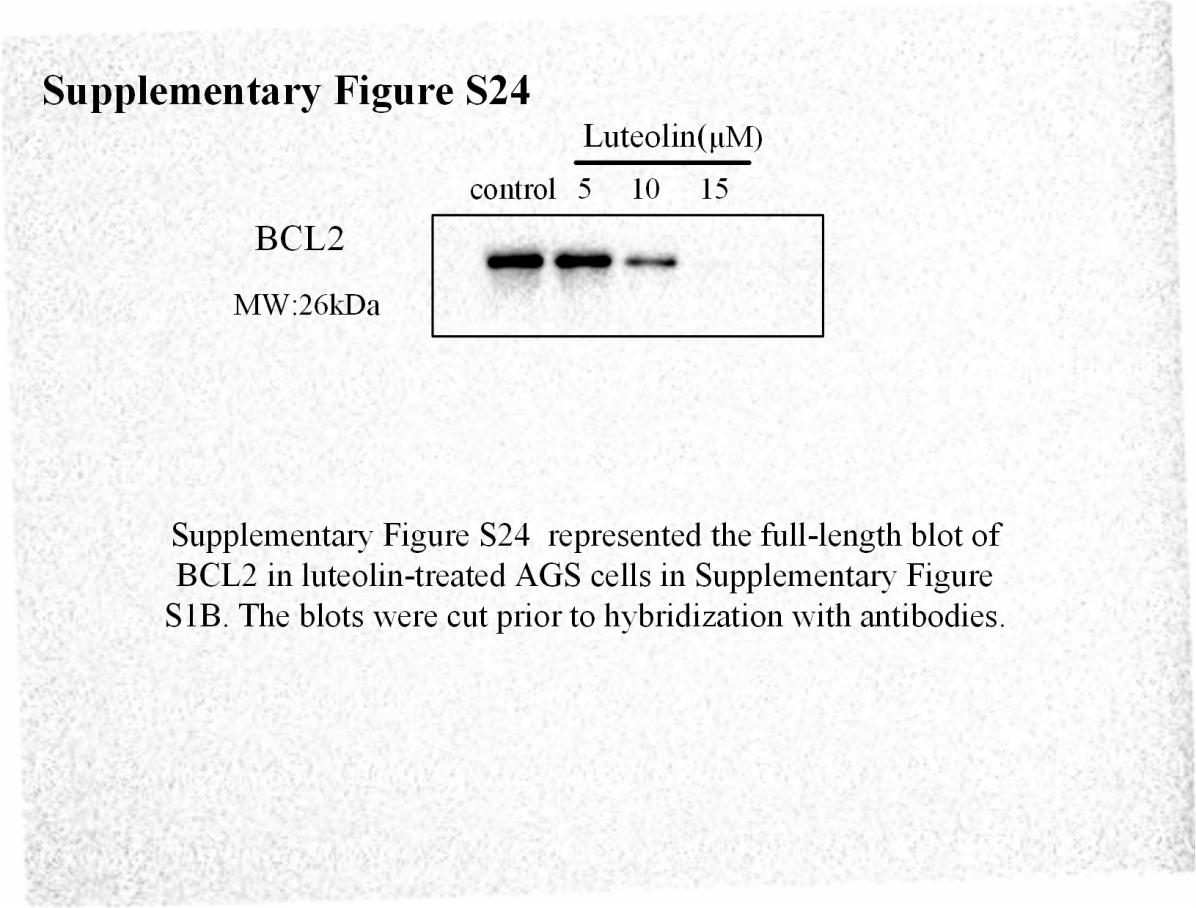


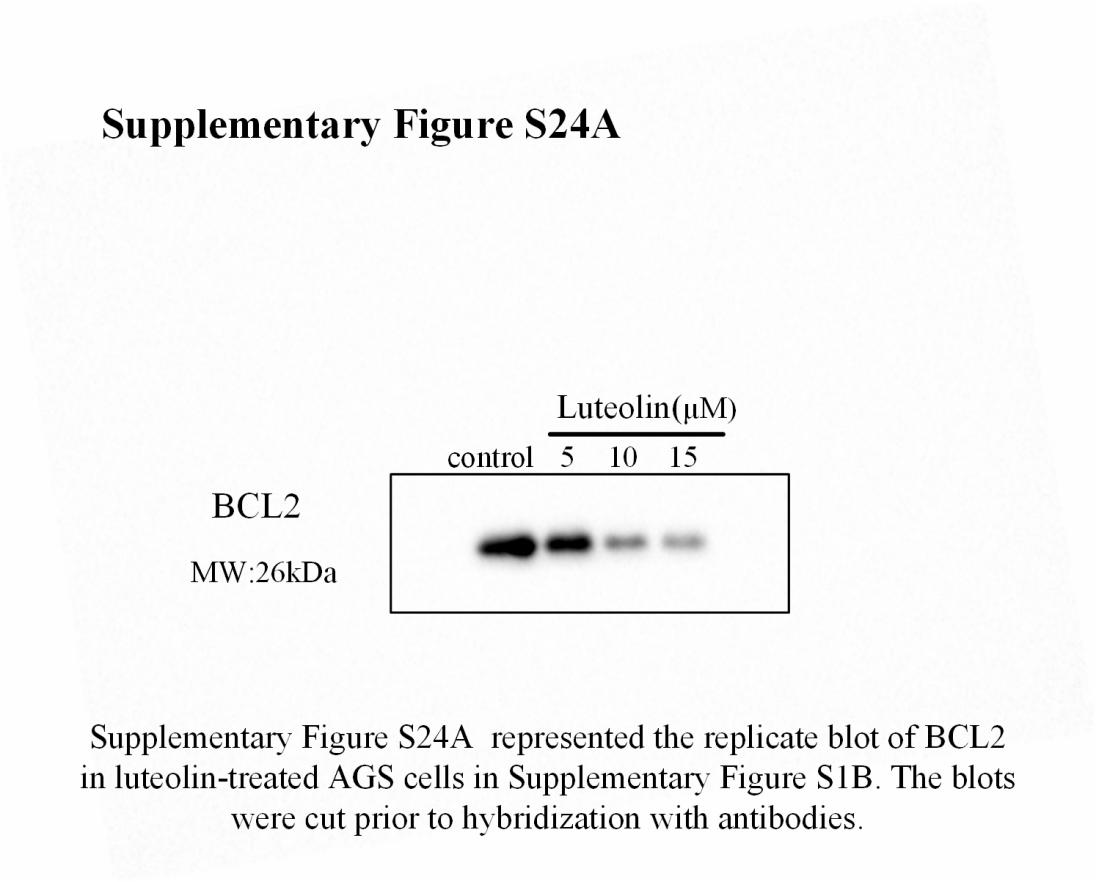

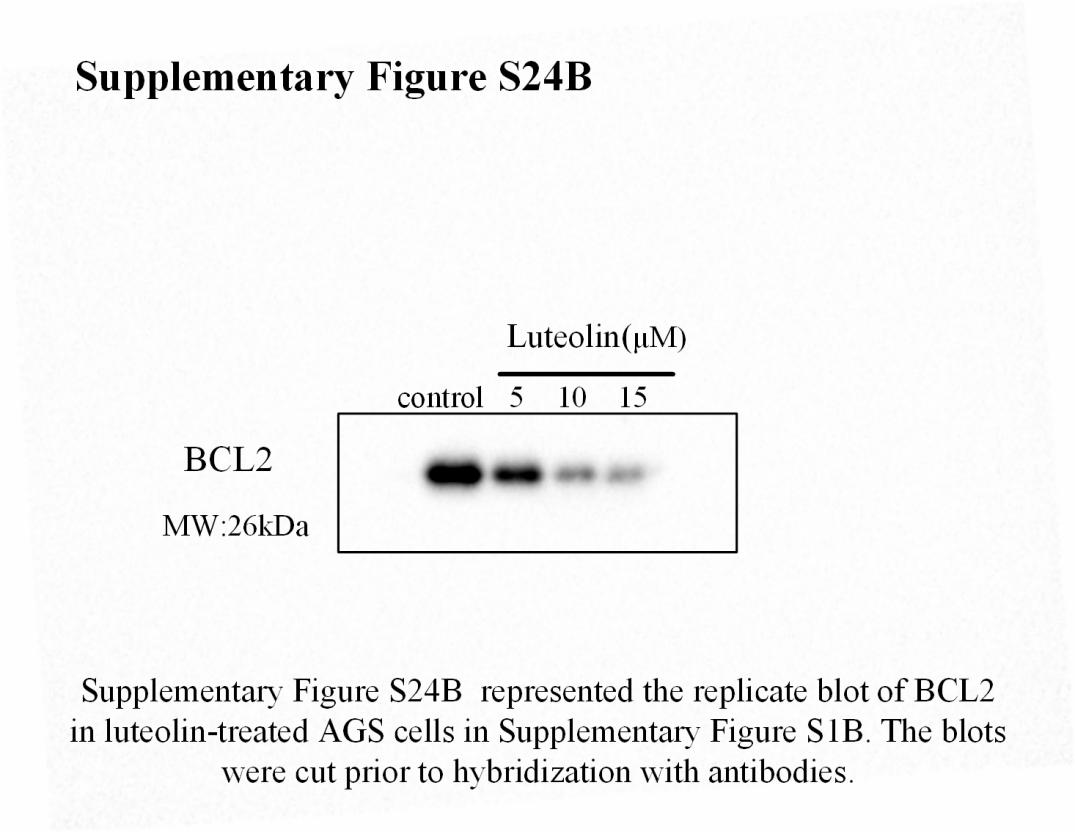


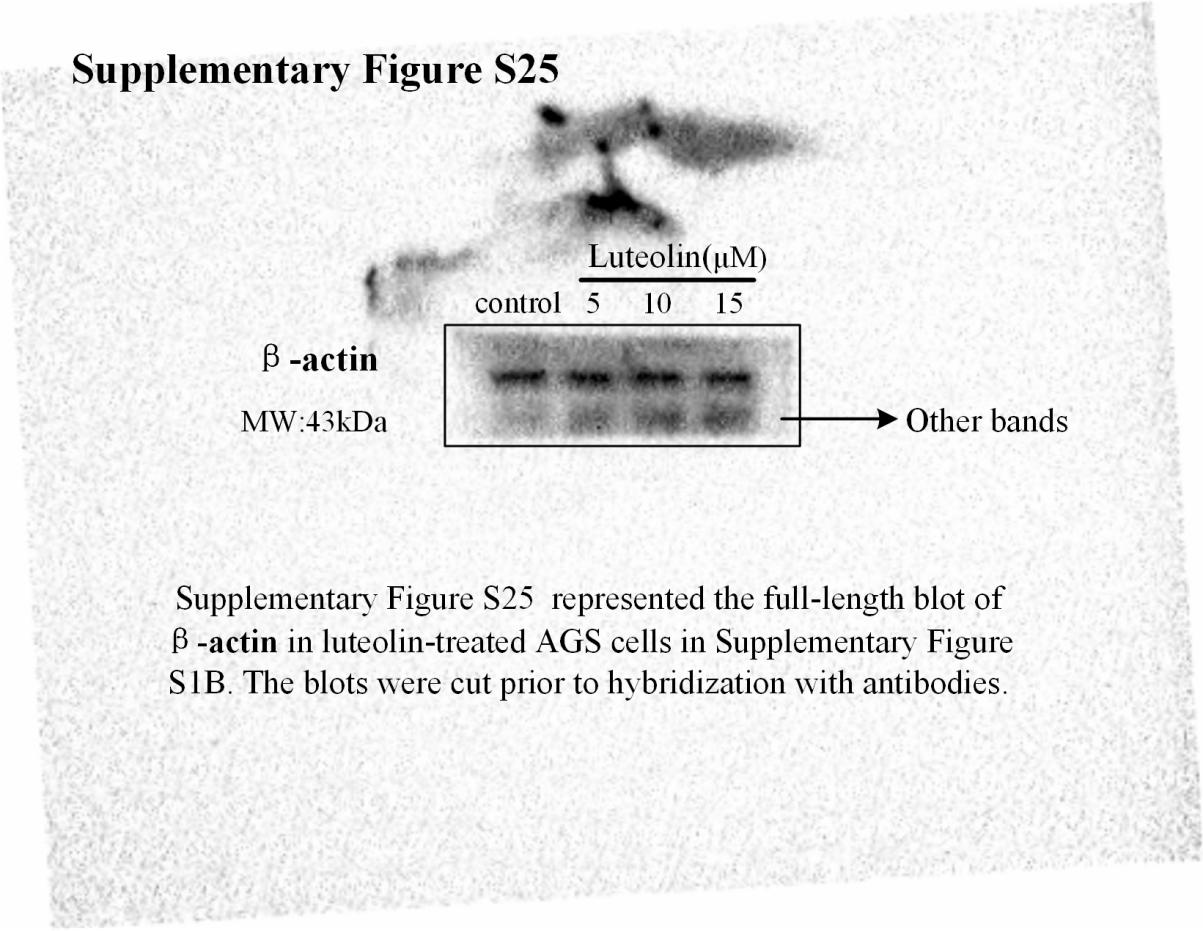


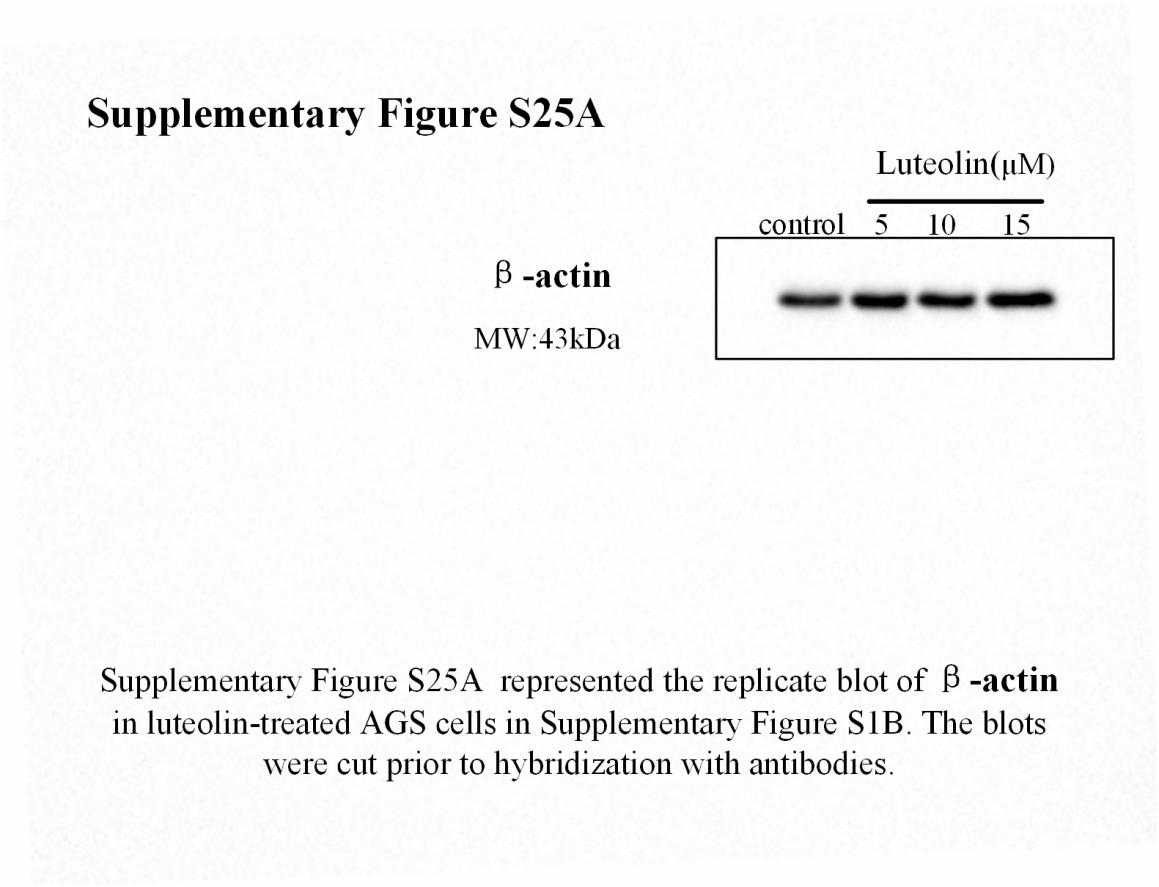


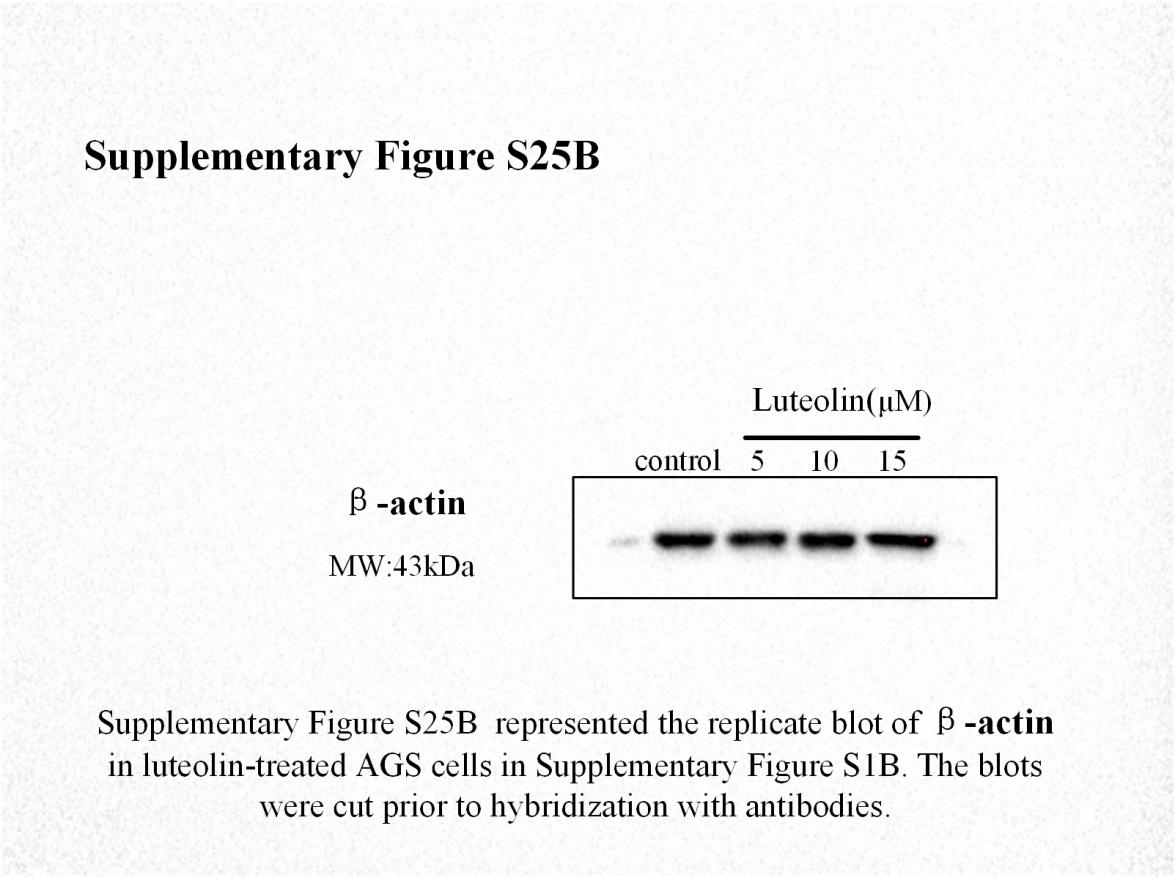

Supplement: Supplementary file 1 — Supplementary Figures. [file 41598_2021_1535_MOESM1_ESM.docx]
