# Supplementary material for: Systems pharmacology to reveal multi-scale mechanisms of traditional Chinese medicine for gastric cancer
Source: Sci Rep. 2021 Nov 12;11:22149. doi: 10.1038/s41598-021-01535-5 (PMC8589993; doi:10.1038/s41598-021-01535-5)
Supplement: Supplementary file 2 — Supplementary Tables. [file 41598_2021_1535_MOESM2_ESM.docx]

**Supplementary Table S1. Chemical information of 83 active compounds and their network parameters**

| MOL | Molecule_Name | OB% | DL | Degree | Herb | Structure |
| --- | --- | --- | --- | --- | --- | --- |
| MOL01 | 24-Ethylcholest-4-en-3-one | 36.08 | 0.76 | 2 | *Pinellia ternata (Thunb.) Breit.* |  |
| MOL02 | cavidine | 35.64 | 0.81 | 21 | *Pinellia ternata (Thunb.) Breit.* |  |
| MOL03 | baicalein | 33.52 | 0.21 | 29 | *Pinellia ternata (Thunb.) Breit.* |  |
| MOL04 | baicalin | 40.12 | 0.75 | 4 | *Pinellia ternata (Thunb.) Breit.* |  |
| MOL05 | beta-sitosterol | 36.91 | 0.75 | 29 | *Pinellia ternata (Thunb.) Breit.,Rheum palmatum L.* |  |
| MOL06 | stigmasterol | 43.83 | 0.76 | 22 | *Pinellia ternata (Thunb.) Breit.,Radix Aucklandiae* |  |
| MOL07 | gondoic acid | 30.70 | 0.20 | 5 | *Pinellia ternata (Thunb.) Breit.* |  |
| MOL08 | coniferin | 31.11 | 0.32 | 16 | *Pinellia ternata (Thunb.) Breit.* |  |
| MOL09 | 10,13-eicosadienoic | 39.99 | 0.20 | 7 | *Pinellia ternata (Thunb.) Breit.* |  |
| MOL10 | cycloartenol | 38.69 | 0.78 | 2 | *Pinellia ternata (Thunb.) Breit.* |  |
| MOL11 | beta-D-Ribofuranoside, xanthine-9 | 44.72 | 0.21 | 6 | *Pinellia ternata (Thunb.) Breit.* |  |
| MOL12 | 12,13-epoxy-9-hydroxynonadeca-7,10-dienoic acid | 42.15 | 0.24 | 4 | *Pinellia ternata (Thunb.) Breit.* |  |
| MOL13 | (3S,6S)-3-(benzyl)-6-(4-hydroxybenzyl)piperazine-2,5-quinone | 46.89 | 0.27 | 12 | *Pinellia ternata (Thunb.) Breit.* |  |
| MOL14 | beta-elemene | 25.63 | 0.06 | 11 | *Pinellia ternata (Thunb.) Breit.* |  |
| MOL15 | flavonoid K | 50.80 | 0.41 | 15 | *Rheum palmatum L.* |  |
| MOL16 | mutatochrome | 48.64 | 0.61 | 7 | *Rheum palmatum L.* |  |
| MOL17 | procyanidin B-5,3'-O-gallate | 31.99 | 0.32 | 15 | *Rheum palmatum L.* |  |
| MOL18 | rhein | 47.07 | 0.28 | 15 | *Rheum palmatum L.* |  |
| MOL19 | sennoside E_qt | 50.69 | 0.61 | 9 | *Rheum palmatum L.* |  |
| MOL20 | torachrysone-8-O-beta-D-(6'-oxayl)-glucoside | 43.02 | 0.74 | 1 | *Rheum palmatum L.* |  |
| MOL21 | emodin-1-O-beta-D-glucopyranoside | 44.81 | 0.80 | 1 | *Rheum palmatum L.* |  |
| MOL22 | sennoside D_qt | 61.06 | 0.61 | 8 | *Rheum palmatum L.* |  |
| MOL23 | daucosterol_qt | 35.89 | 0.70 | 3 | *Rheum palmatum L.* |  |
| MOL24 | palmidin A | 32.45 | 0.65 | 6 | *Rheum palmatum L.* |  |
| MOL25 | aloe-emodin | 83.38 | 0.24 | 29 | *Rheum palmatum L.* |  |
| MOL26 | toralactone | 46.46 | 0.24 | 12 | *Rheum palmatum L.* |  |
| MOL27 | (-)-catechin | 49.68 | 0.24 | 15 | *Rheum palmatum L.* |  |
| MOL28 | emodinanthrone | 24.72 | 0.21 | 22 | *Rheum palmatum L.* |  |
| MOL29 | palmidin B | 1.21 | 0.69 | 7 | *Rheum palmatum L.* |  |
| MOL30 | palmidin C | 2.35 | 0.69 | 6 | *Rheum palmatum L.* |  |
| MOL31 | rheidin B | 1.21 | 0.65 | 9 | *Rheum palmatum L.* |  |
| MOL32 | rheidin C | 1.24 | 0.58 | 9 | *Rheum palmatum L.* |  |
| MOL33 | emodin | 24.40 | 0.24 | 34 | *Rheum palmatum L.* |  |
| MOL34 | sesamin | 56.55 | 0.83 | 23 | *Gentiana scabra Bunge* |  |
| MOL35 | isovitexin | 31.29 | 0.72 | 7 | *Gentiana scabra Bunge* |  |
| MOL36 | leucanthoside | 32.12 | 0.78 | 3 | *Gentiana scabra Bunge* |  |
| MOL37 | gentirigenic acid | 38.78 | 0.78 | 6 | *Gentiana scabra Bunge* |  |
| MOL38 | gentisin | 64.06 | 0.21 | 13 | *Gentiana scabra Bunge* |  |
| MOL39 | gentiopicroside tetraacetate | 32.44 | 0.75 | 1 | *Gentiana scabra Bunge* |  |
| MOL40 | gentisein | 67.57 | 0.19 | 9 | *Gentiana scabra Bunge* |  |
| MOL41 | sitosterol | 36.91 | 0.75 | 2 | *Gentiana scabra Bunge，Radix Aucklandiae* |  |
| MOL42 | kaempferol | 41.88 | 0.24 | 48 | *Gentiana scabra Bunge* |  |
| MOL43 | pranferin | 52.14 | 0.28 | 11 | *Gentiana scabra Bunge* |  |
| MOL44 | oleanolic acid | 29.02 | 0.76 | 6 | *Gentiana scabra Bunge* |  |
| MOL45 | gentianal | 7.52 | 0.08 | 6 | *Gentiana scabra Bunge* |  |
| MOL46 | chinoinin | 13.71 | 0.75 | 4 | *Gentiana scabra Bunge* |  |
| MOL47 | swertiamarin | 21.90 | 0.42 | 1 | *Gentiana scabra Bunge* |  |
| MOL48 | ursolic acid | 16.77 | 0.75 | 43 | *Gentiana scabra Bunge* |  |
| MOL49 | swertiamarin_qt | 2.58 | 0.09 | 7 | *Gentiana scabra Bunge* |  |
| MOL50 | gentiopicroside | 22.98 | 0.39 | 5 | *Gentiana scabra Bunge* |  |
| MOL51 | gentianine | 54.67 | 0.06 | 16 | *Gentiana scabra Bunge* |  |
| MOL52 | sweroside aglycone | 68.68 | 0.08 | 5 | *Gentiana scabra Bunge* |  |
| MOL53 | isoorientin | 23.30 | 0.76 | 4 | *Gentiana scabra Bunge* |  |
| MOL54 | benzo[a]carbazole | 35.22 | 0.22 | 8 | *Radix Aucklandiae* |  |
| MOL55 | lappadilactone | 38.56 | 0.73 | 1 | *Radix Aucklandiae* |  |
| MOL56 | mairin | 55.38 | 0.78 | 2 | *Radix Aucklandiae* |  |
| MOL57 | cynaropicrin | 67.50 | 0.38 | 5 | *Radix Aucklandiae* |  |
| MOL58 | costunolide | 29.07 | 0.11 | 8 | *Radix Aucklandiae* |  |
| MOL59 | costuslactone | 60.48 | 0.11 | 7 | *Radix Aucklandiae* |  |
| MOL60 | dihydrocostus lactone | 62.30 | 0.11 | 5 | *Radix Aucklandiae* |  |
| MOL61 | isoalantolactone | 53.43 | 0.15 | 3 | *Radix Aucklandiae* |  |
| MOL62 | isodihydrocostunolide | 43.44 | 0.12 | 3 | *Radix Aucklandiae* |  |
| MOL63 | beta-Ionone | 20.63 | 0.05 | 6 | *Radix Aucklandiae* |  |
| MOL64 | poncirin | 36.55 | 0.74 | 6 | *Citrus aurantium L.* |  |
| MOL65 | isosinens  etin | 51.15 | 0.44 | 15 | *Citrus aurantium L.* |  |
| MOL66 | 5,7,4'-trimethylapigenin | 39.83 | 0.30 | 16 | *Citrus aurantium L.* |  |
| MOL67 | 6-methoxy aurapten | 31.24 | 0.30 | 13 | *Citrus aurantium L.* |  |
| MOL68 | neohesperidin_qt | 71.17 | 0.27 | 13 | *Citrus aurantium L.* |  |
| MOL69 | sinensetin | 50.56 | 0.45 | 15 | *Citrus aurantium L.* |  |
| MOL70 | eriodyctiol (flavanone) | 41.35 | 0.24 | 13 | *Citrus aurantium L.* |  |
| MOL71 | naringenin | 59.29 | 0.21 | 36 | *Citrus aurantium L.* |  |
| MOL72 | 5,7-dihydroxy-2-(3-hydroxy-4-methoxyphenyl)chroman-4-one | 47.74 | 0.27 | 13 | *Citrus aurantium L.* |  |
| MOL73 | nobiletin | 61.67 | 0.52 | 23 | *Citrus aurantium L.* |  |
| MOL74 | didymin | 38.55 | 0.24 | 14 | *Citrus aurantium L.* |  |
| MOL75 | luteolin | 36.16 | 0.25 | 49 | *Citrus aurantium L.* |  |
| MOL76 | tetramethoxyluteolin | 43.68 | 0.37 | 19 | *Citrus aurantium L.* |  |
| MOL77 | 4-[(2S,3R)-5-[(E)-3-hydroxyprop-1-enyl]-7-methoxy-3-methylol-2,3-dihydrobenzofuran-2-yl]-2-methoxy-phenol | 50.76 | 0.39 | 14 | *Citrus aurantium L.* |  |
| MOL78 | prangenin | 43.60 | 0.29 | 7 | *Citrus aurantium L.* |  |
| MOL79 | poncimarin | 63.62 | 0.35 | 6 | *Citrus aurantium L.* |  |
| MOL80 | isoponcimarin | 63.28 | 0.31 | 8 | *Citrus aurantium L.* |  |
| MOL81 | citrusin B | 40.80 | 0.71 | 1 | *Citrus aurantium L.* |  |
| MOL82 | ammidin | 34.55 | 0.22 | 12 | *Citrus aurantium L.* |  |
| MOL83 | hesperidin | 13.33 | 0.67 | 5 | *Citrus aurantium L.* |  |

**Supplementary Table S2. The information of gastric cancer-related targets**

| **No.** | **Gene-name** | **Protein-Name** | **Degree** |
| --- | --- | --- | --- |
| 1 | ESR1 | Estrogen receptor | 47 |
| 2 | AR | Androgen receptor | 45 |
| 3 | NOS2 | Nitric oxide synthase, inducible | 46 |
| 4 | PTGS1 | Prostaglandin G/H synthase 1 | 31 |
| 5 | CHRM3 | Muscarinic acetylcholine receptor M3 | 8 |
| 6 | F2 | Prothrombin | 39 |
| 7 | ADRB1 | Beta-1 adrenergic receptor | 3 |
| 8 | PPARG | Peroxisome proliferator-activated receptor gamma | 38 |
| 9 | PTGS2 | Prostaglandin G/H synthase 2 | 44 |
| 10 | NOS3 | Nitric oxide synthase, endothelial | 20 |
| 11 | ACHE | Acetylcholinesterase | 22 |
| 12 | ADRA1B | Alpha-1B adrenergic receptor | 10 |
| 13 | PTPN1 | Tyrosine-protein phosphatase non-receptor type 1 | 32 |
| 14 | ADRB2 | Beta-2 adrenergic receptor | 15 |
| 15 | OPRM1 | Mu-type opioid receptor | 4 |
| 16 | ESR2 | Estrogen receptor beta | 29 |
| 17 | MAPK14 | Mitogen-activated protein kinase 14 | 28 |
| 18 | GSK3B | Glycogen synthase kinase-3 beta | 33 |
| 19 | RXRB | Retinoic acid receptor RXR-beta | 1 |
| 20 | PRSS1 | Trypsin-1 | 33 |
| 21 | CCNA2 | Cyclin-A2 | 29 |
| 22 | CA2 | Carbonic anhydrase II | 39 |
| 23 | RELA | Transcription factor p65 | 6 |
| 24 | AKT1 | RAC-alpha serine/threonine-protein kinase | 4 |
| 25 | VEGFA | Vascular endothelial growth factor A | 3 |
| 26 | BCL2 | Apoptosis regulator Bcl-2 | 7 |
| 27 | FOS | Proto-oncogene c-Fos | 2 |
| 28 | BAX | Apoptosis regulator BAX | 7 |
| 29 | MMP9 | Matrix metalloproteinase-9 | 5 |
| 30 | CASP3 | Caspase-3 | 10 |
| 31 | TP53 | Cellular tumor antigen p53 | 6 |
| 32 | HIF1A | Hypoxia-inducible factor 1-alpha | 1 |
| 33 | CDK1 | Cyclin-dependent kinase 1 | 3 |
| 34 | MPO | Myeloperoxidase\|Myeloperoxidase ([Ferris, Blumenschein et al.](#_ENREF_1)) | 2 |
| 35 | AHR | Aryl hydrocarbon receptor | 2 |
| 36 | IGF2 | Insulin-like growth factor II | 1 |
| 37 | CYCS | Cytochrome c | 1 |
| 38 | FABP5 | Fatty acid-binding protein, epidermal | 1 |
| 39 | ADRA1A | Alpha-1A adrenergic receptor | 4 |
| 40 | GABRA1 | Gamma-aminobutyric-acid receptor subunit alpha-1 | 16 |
| 41 | JUN | Transcription factor AP-1 | 6 |
| 42 | CASP8 | Caspase-8 | 2 |
| 43 | PRKCA | Protein kinase C alpha type | 2 |
| 44 | TGFB1 | Transforming growth factor beta-1 | 2 |
| 45 | PON1 | Serum paraoxonase/arylesterase 1 | 1 |
| 46 | AKR1B1 | Aldose reductase | 4 |
| 47 | PLAU | Urokinase-type plasminogen activator | 2 |
| 48 | LTA4H | Leukotriene A-4 hydrolase | 1 |
| 49 | MAOB | Amine oxidase [flavin-containing] B | 5 |
| 50 | MAOA | Amine oxidase [flavin-containing] A | 1 |
| 51 | PNP | Purine nucleoside phosphorylase | 1 |
| 52 | KDR | Vascular endothelial growth factor receptor 2 | 2 |
| 53 | PPARD | Peroxisome proliferator activated receptor delta | 1 |
| 54 | SRD5A1 | 3-oxo-5-alpha-steroid 4-dehydrogenase 1 | 2 |
| 55 | KCNQ1 | Potassium voltage-gated channel subfamily KQT member 1 | 1 |
| 56 | HSD11B2 | Corticosteroid 11-beta-dehydrogenase isozyme 2 | 11 |
| 57 | HPSE | Heparanase | 3 |
| 58 | DHCR7 | 7-dehydrocholesterol reductase | 2 |
| 59 | PTPA | Serine/threonine-protein phosphatase 2A activator | 1 |
| 60 | ENPP2 | Autotaxin | 3 |
| 61 | TEK | Angiopoietin-1 receptor | 1 |
| 62 | F3 | Coagulation factor III | 1 |
| 63 | KISS1R | KiSS-1 receptor | 10 |
| 64 | CCK | Cholecystokinin | 1 |
| 65 | ITGAL | Integrin alpha-L | 1 |
| 66 | SMO | Smoothened homolog | 1 |
| 67 | SLC10A2 | Ileal sodium/bile acid cotransporter | 2 |
| 68 | EDNRB | Endothelin receptor type B | 1 |
| 69 | NR1H2 | Oxysterols receptor LXR-beta | 8 |
| 70 | ABCC1 | Multidrug resistance-associated protein 1 | 2 |
| 71 | PRKCD | Protein kinase C delta type | 3 |
| 72 | AKR1C3 | Aldo-keto reductase family 1 member C3 | 4 |
| 73 | SLC16A1 | Monocarboxylate Transporter 1 | 7 |
| 74 | ALOX5 | Arachidonate 5-lipoxygenase | 7 |
| 75 | IGFBP3 | Insulin-like growth factor-binding protein 3 | 4 |
| 76 | CBR1 | Carbonyl reductase [NADPH] 1 | 7 |
| 77 | TSPO | Translocator protein | 5 |
| 78 | TNK2 | Activated CDC42 kinase 1 | 1 |
| 79 | CDKN1A | Cyclin-dependent kinase inhibitor 1 | 4 |
| 80 | TNF | Tumor necrosis factor | 6 |
| 81 | FASN | Fatty acid synthase | 5 |
| 82 | PRKCE | Protein kinase C epsilon type | 2 |
| 83 | PCNA | Proliferating cell nuclear antigen | 2 |
| 84 | MYC | Myc proto-oncogene protein | 2 |
| 85 | IL1B | Interleukin-1 beta | 3 |
| 86 | NOS1 | Nitric Oxide Synthase, brain | 1 |
| 87 | CYP2A6 | Cytochrome P450 2A6 | 3 |
| 88 | PRF1 | Perforin-1 | 1 |
| 89 | CCR6 | C-C chemokine receptor type 6 | 1 |
| 90 | SMAD3 | mothers against decapentaplegic homolog 3 | 3 |
| 91 | PLAA | Phospholipase A-2-activating protein | 2 |
| 92 | MST1R | Macrophage-stimulating protein receptor | 1 |
| 93 | FLT1 | Vascular endothelial growth factor receptor 1 | 1 |
| 94 | EGF | Pro-epidermal growth factor | 1 |
| 95 | MMP1 | Interstitial collagenase | 4 |
| 96 | CYP1A1 | Cytochrome P450 1A1 | 2 |
| 97 | CSF2 | Granulocyte-macrophage colony-stimulating factor | 2 |
| 98 | ACTA2 | Actin, aortic smooth muscle | 1 |
| 99 | SLC2A1 | Solute carrier family 2, facilitated glucose transporter member 1 | 1 |
| 100 | CCND1 | G1/S-specific cyclin-D1 | 3 |
| 101 | IL10 | Interleukin-10 | 2 |
| 102 | ACACA | Acetyl-CoA carboxylase 1 | 1 |
| 103 | ECE1 | Endothelin-converting enzyme 1 | 1 |
| 104 | ACADM | Medium-chain specific acyl-CoA dehydrogenase, mitochondrial | 1 |
| 105 | CYP2B6 | Cytochrome P450 2B6 | 1 |
| 106 | SREBF1 | Sterol regulatory element-binding protein 1 | 2 |
| 107 | NOX1 | NADPH oxidase 1 | 1 |
| 108 | ACOX1 | Peroxisomal acyl-coenzyme A oxidase 1 | 1 |
| 109 | ACLY | ATP-citrate synthase | 1 |
| 110 | HADHB | Trifunctional enzyme subunit beta, mitochondrial | 1 |
| 111 | AKR1B10 | Aldo-keto reductase family 1 member B10 | 1 |
| 112 | CYP17A1 | Steroid 17-alpha-hydroxylase/17,20 lyase | 2 |
| 113 | MAPK8 | Mitogen-activated protein kinase 8 | 3 |
| 114 | STAT1 | Signal transducer and activator of transcription 1-alpha/beta | 1 |
| 115 | HMOX1 | Heme oxygenase 1 | 3 |
| 116 | CYP3A4 | Cytochrome P450 3A4 | 1 |
| 117 | CYP1A2 | Cytochrome P450 1A2 | 1 |
| 118 | ICAM1 | Intercellular adhesion molecule 1 | 5 |
| 119 | VCAM1 | Vascular cell adhesion protein 1 | 2 |
| 120 | NR1I2 | Pregnane X receptor | 2 |
| 121 | CYP1B1 | Cytochrome P450 1B1 | 1 |
| 122 | GSTP1 | Glutathione S-transferase P | 3 |
| 123 | NR1I3 | Nuclear receptor subfamily 1 group I member 3 | 1 |
| 124 | DIO1 | Type I iodothyronine deiodinase | 1 |
| 125 | PPP3CA | Serine/threonine-protein phosphatase 2B catalytic subunit alpha isoform | 1 |
| 126 | GSTM1 | Glutathione S-transferase Mu 1 | 1 |
| 127 | GSTM2 | Glutathione S-transferase Mu 2 | 1 |
| 128 | SLPI | Antileukoproteinase | 1 |
| 129 | NQO1 | NAD(P)H dehydrogenase [quinone] 1 | 2 |
| 130 | NQO2 | NRH dehydrogenase [quinone] 2 | 4 |
| 131 | STAT3 | Signal transducer and activator of transcription 3 | 1 |
| 132 | BCL2L1 | Bcl-2-like protein 1 | 2 |
| 133 | MMP2 | 72 kDa type IV collagenase | 2 |
| 134 | CDK4 | Cell division protein kinase 4 | 2 |
| 135 | IL6 | Interleukin-6 | 2 |
| 136 | NFKBIA | NF-kappa-B inhibitor alpha | 2 |
| 137 | FGF2 | Heparin-binding growth factor 2 | 1 |
| 138 | MMP10 | Stromelysin-2 | 1 |
| 139 | MCL1 | Induced myeloid leukemia cell differentiation protein Mcl-1 | 2 |
| 140 | BIRC5 | Baculoviral IAP repeat-containing protein 5 | 2 |
| 141 | GAP43 | Neuromodulin | 1 |
| 142 | INPPL1 | Phosphatidylinositol-3,4,5-trisphosphate 5-phosphatase 2 | 1 |
| 143 | CCND2 | G1/S-specific cyclin-D2 | 1 |
| 144 | FASLG | Tumor necrosis factor ligand superfamily member 6 | 1 |
| 145 | CASP1 | Caspase-1 | 1 |
| 146 | NFE2L2 | Nuclear factor erythroid 2-related factor 2 | 1 |
| 147 | CNR2 | Cannabinoid receptor 2 | 1 |
| 148 | CHRNA4 | Neuronal acetylcholine receptor subunit alpha-4 | 2 |
| 149 | ENPEP | Aminopeptidase A | 1 |
| 150 | RARA | Retinoic acid receptor alpha | 1 |
| 151 | BDKRB1 | B1 bradykinin receptor | 1 |
| 152 | F2R | Proteinase activated receptor 1 | 1 |
| 153 | ODC1 | Ornithine decarboxylase | 1 |
| 154 | ABCC8 | Sulfonylurea receptor 1 | 1 |
| 155 | MAPK3 | Mitogen-activated protein kinase 3 | 1 |
| 156 | MAPK1 | Mitogen-activated protein kinase 1 | 2 |
| 157 | LDLR | Low-density lipoprotein receptor | 1 |
| 158 | SOD1 | Superoxide dismutase [Cu-Zn] | 1 |
| 159 | CAT | Catalase | 1 |
| 160 | MTTP | Microsomal triglyceride transfer protein large subunit | 1 |
| 161 | APOB | Apolipoprotein B-100 | 1 |
| 162 | HMGCR | 3-hydroxy-3-methylglutaryl-coenzyme A reductase | 1 |
| 163 | CYP19A1 | Cytochrome P450 19A1 | 1 |
| 164 | PPARA | Peroxisome proliferator-activated receptor alpha | 1 |
| 165 | GSR | Glutathione reductase, mitochondrial | 1 |
| 166 | ADIPOQ | Adiponectin | 1 |
| 167 | AKR1C1 | Aldo-keto reductase family 1 member C1 | 1 |
| 168 | GOT1 | Aspartate aminotransferase, cytoplasmic | 1 |
| 169 | ABAT | 4-aminobutyrate aminotransferase, mitochondrial | 1 |
| 170 | SOAT1 | Sterol O-acyltransferase 1 | 1 |
| 171 | TIMP1 | Metalloproteinase inhibitor 1 | 1 |
| 172 | PLA2G4A | Cytosolic phospholipase A2 | 1 |
| 173 | CD163 | Scavenger receptor cysteine-rich type 1 protein M130 | 1 |
| 174 | EGFR | Epidermal growth factor receptor | 1 |
| 175 | RB1 | Retinoblastoma-associated protein | 1 |
| 176 | APP | Amyloid beta A4 protein | 1 |
| 177 | ERBB2 | Receptor tyrosine-protein kinase erbB-2 | 1 |
| 178 | CASP7 | Caspase-7 | 1 |
| 179 | IL2 | Interleukin-2 | 1 |
| 180 | IFNG | Interferon gamma | 1 |
| 181 | IL4 | Interleukin-4 | 1 |
| 182 | XIAP | Baculoviral IAP repeat-containing protein 4 | 1 |
| 183 | PTGES | Prostaglandin E synthase | 1 |
| 184 | MET | Hepatocyte growth factor receptor | 1 |

**Supplementary Table S3. The correspondence between potential compounds and targets**

| **MOL** | **Compound** | **Target** |
| --- | --- | --- |
| MOL01 | 24-Ethylcholest-4-en-3-one | ESR1，AR |
| MOL02 | Cavidine | NOS2，PTGS1，CHRM3，F2，ESR1，AR，ADRB1，PPARG，PTGS2，NOS3，ACHE，ADRA1B，PTPN1，ADRB2，OPRM1，ESR2，MAPK14，GSK3B，RXRB，PRSS1，CCNA2 |
| MOL03 | baicalein | NOS2，PTGS1，ESR1，AR，PPARG，PTGS2，CA2，PTPN1，ESR2，MAPK14，GSK3B，CCNA2，PRSS1，RELA，AKT1，VEGFA，BCL2，FOS，BAX，MMP9，CASP3，TP53，HIF1A，CDK1，MPO，AHR，IGF2，CYCS，FABP5 |
| MOL04 | Baicalin | CA2，PTPN1，GSK3B，CCNA2 |
| MOL05 | beta-sitosterol | ESR1，AR，NOS2，PTGS1，PPARG，PTGS2，CA2，PTPN1，ESR2，MAPK14，GSK3B，CCNA2，F2，PRSS1，CHRM3，ADRA1A，ADRA1B，ADRB2，OPRM1，GABRA1，ACHE，BCL2，BAX，JUN，CASP3，CASP8，PRKCA，TGFB1，PON1 |
| MOL06 | Stigmasterol | ESR1，AR，F2，NOS3，ACHE，PRSS1，NOS2，PTGS1，PPARG，PTGS2，CA2，ADRB2，AKR1B1，PLAU，LTA4H，MAOB，MAOA，CHRM3，ADRB1，ADRA1A，ADRA1B，GABRA1 |
| MOL07 | gondoic acid | PTGS1，F2，PPARG，NOS3，ACHE |
| MOL08 | coniferin | NOS2，CHRM3，F2，ESR1，AR，PPARG，PTGS2，CA2，ACHE，ADRA1B，ADRB2，OPRM1，ESR2，GSK3B，PRSS1，CCNA2 |
| MOL09 | 10,13-eicosadienoic | NOS2，PTGS1，F2，PPARG，NOS3，CA2，ACHE |
| MOL10 | Cycloartenol | ESR1，AR |
| MOL11 | beta-D-Ribofuranoside, xanthine-9 | NOS2，F2，ESR1，PNP，PTGS2，CCNA2 |
| MOL12 | 12,13-epoxy-9-hydroxynonadeca-7,10-dienoic acid | F2，ESR1，PPARG，NOS3 |
| MOL13 | (3S,6S)-3-(benzyl)-6-(4-hydroxybenzyl)piperazine-2,5-quinone | NOS2，F2，ESR1，AR，PPARG，PTGS2，CA2，ADRB2，MAPK14，GSK3B，PRSS1，CCNA2 |
| MOL14 | beta-elemene | F2，PTGS2，NOS3，ACHE，GABRA1，PRSS1，PTGS1，CHRM3，CA2，ADRA1A，ESR1 |
| MOL15 | Flavonoid K | NOS2，ESR1，AR，PPARG，PTGS2，CA2，PTPN1，ESR2，GSK3B，PRSS1，CCNA2，F2，KDR，PPARD，MAPK14 |
| MOL16 | Mutatochrome | SRD5A1，KCNQ1，HSD11B2，HPSE，DHCR7，PTPA，ENPP2 |
| MOL17 | Procyanidin B-5,3'-O-gallate | TEK，HSD11B2，F3，KISS1R，CCK，ITGAL，SMO，HPSE，SLC10A2，EDNRB，NR1H2，BCL2，ABCC1，PRKCD，ENPP2 |
| MOL18 | rhein | PTGS1，ESR1，AR，PTGS2，CA2，PTPN1，ESR2，MAPK14，GSK3B，NOS2，PPARG，F2，AKR1B1，CCNA2，JUN |
| MOL19 | Sennoside E_qt | AKR1C3，HSD11B2，KISS1R，SLC16A1，ALOX5，IGFBP3，CBR1，NR1H2，TSPO |
| MOL20 | Torachrysone-8-O-beta-D-(6'-oxayl)-glucoside | CCNA2 |
| MOL21 | Emodin-1-O-beta-D-glucopyranoside | AR |
| MOL22 | Sennoside D_qt | AKR1C3，HSD11B2，KISS1R，SLC16A1，ALOX5，IGFBP3，CBR1，NR1H2 |
| MOL23 | Daucosterol_qt | F2，ESR1，AR |
| MOL24 | palmidin A | HSD11B2，KISS1R，SLC16A1，TNK2，CBR1，NR1H2 |
| MOL25 | aloe-emodin | NOS2，PTGS1，ESR1，AR，PPARG，PTGS2，NOS3，CA2，ACHE，PTPN1，ESR2，MAPK14，GSK3B，CCNA2，AKR1B1，PRSS1，CDKN1A，BAX，TNF，CASP3，TP53，FASN，PRKCA，PRKCE，CDK1，PCNA，MYC，IL1B，PRKCD |
| MOL26 | Toralactone | NOS2，PTGS1，ESR1，AR，PPARG，PTGS2，CA2，PTPN1，ESR2，MAPK14，GSK3B，CCNA2 |
| MOL27 | (-)-catechin | NOS2，PTGS1，ESR1，AR，PPARG，PTGS2，CA2，PTPN1，ESR2，MAPK14，GSK3B，CCNA2，PRSS1，FASN，PPARG |
| MOL28 | Emodinanthrone | NOS1，CYP2A6，ALOX5，ALOX5，PRF1，CCR6，SMAD3，PLAA，NOS2，PTGS1，F2，ESR1，AR，PPARG，PTGS2，CA2，PTPN1，ADRB2，ESR2，MAPK14，GSK3B，PRSS1，CCNA2 |
| MOL29 | palmidin B | HSD11B2，KISS1R，SLC16A1，ALOX5，CBR1，NR1H2，TSPO |
| MOL30 | Palmidin C | HSD11B2，KISS1R，SLC16A1，CBR1，NR1H2，TSPO |
| MOL31 | Rheidin B | AKR1C3，HSD11B2，KISS1R，SLC16A1，ALOX5，IGFBP3，CBR1，NR1H2，TSPO |
| MOL32 | Rheidin C | MST1R，HSD11B2，KISS1R，SLC16A1，ALOX5，IGFBP3，CBR1，NR1H2，TSPO |
| MOL33 | emodin | NOS2，PTGS1，ESR1，AR，PPARG，PTGS2，CA2，PTPN1，ESR2，MAPK14，GSK3B，PRSS1，CCNA2，F2，KDR，CDKN1A，FLT1，MMP9，EGF，TNF，CASP3，TP53，PRKCE，MMP1，PPARG，MYC，CYP1A1，IL1B，PRKCD，CSF2，TGFB1，ACTA2，MAOB，SLC2A1 |
| MOL34 | sesamin | NOS2，ESR1，AR，PTGS2，ACHE，PTPN1，ESR2，MAPK14，GSK3B，PRSS1，CCND1，IL10，FASN，ACACA，NOS3，ECE1，ACADM，CYP2B6，SREBF1，NOX1，ACOX1，ACLY，HADHB |
| MOL35 | isovitexin | PTGS2，PTPN1，AR，CA2，RELA，TNF，NOS2 |
| MOL36 | Leucanthoside | CA2，PTPN1，GSK3B |
| MOL37 | gentirigenic acid | SRD5A1，AKR1B10，HSD11B2，SLC10A2，DHCR7，CYP17A1 |
| MOL38 | Gentisin | NOS2，PTGS1，ESR1，AR，PPARG，PTGS2，CA2，PTPN1，AKR1B1，ESR2，MAPK14，GSK3B，CCNA2 |
| MOL39 | Gentiopicroside tetraacetate | KISS1R |
| MOL40 | Gentisein | NOS2，PTGS1，ESR1，PPARG，PTGS2，CA2，PTPN1，ESR2，MAPK14 |
| MOL41 | sitosterol | ESR1，AR |
| MOL42 | kaempferol | NOS2，PTGS1，ESR1，AR，PPARG，PTGS2，CA2，PTPN1，ESR2，MAPK14，GSK3B，CCNA2，PRSS1，F2，NOS3，ACHE，ADRA1B，GABRA1，RELA，AKT1，BCL2，BAX，TNF，JUN，CASP3，MAPK8，MMP1，STAT1，CDK1，PPARG，HMOX1，CYP3A4，CYP1A2，CYP1A1，ICAM1，VCAM1，NR1I2，CYP1B1，ALOX5，GSTP1，AHR，NR1I3，DIO1，PPP3CA，GSTM1，GSTM2，AKR1C3，SLPI |
| MOL43 | pranferin | NOS2，PTGS1，CHRM3，F2，ESR1，AR，PTGS2，ADRB2，OPRM1，GABRA1，GSK3B |
| MOL44 | oleanolic acid | AR，ESR1，CASP3，HMOX1，ICAM1，NQO1 |
| MOL45 | gentianal | F2，NQO2，NOS3，CA2，GABRA1，PRSS1 |
| MOL46 | Chinoinin | CA2，PTPN1，PRSS1，GSK3B |
| MOL47 | Swertiamarin | HSD11B2 |
| MOL48 | ursolic acid | ESR1，AR，PLAU，RELA，STAT3，VEGFA，CCND1，BCL2，BCL2L1，FOS，CDKN1A，BAX，MMP2，MMP9，CDK4，TNF，JUN，IL6，NOS2，CASP3，TP53，MAPK8，PTGS2，NFKBIA，CASP8，FASN，MMP1，FGF2，MMP10，ICAM1，IL1B，PTGS1，MCL1，ACHE，CSF2，BIRC5，GAP43，NOS3，PTPN1，INPPL1，CCND2，FASLG，CASP1 |
| MOL49 | swertiamarin_qt | MPO，CYP2A6，CYP2A6，NOS2，SMAD3，PLAA，GABRA1，PRSS1 |
| MOL50 | Gentiopicroside | F2，NQO2，CA2，GABRA1，PRSS1 |
| MOL51 | Gentianine | NOS2，PTGS1，CHRM3，F2，PTGS2，NOS3，CA2，ACHE，ADRA1B，ADRB2，GABRA1，MAOB，PRSS1，ADRB1，NQO2，ADRA1A |
| MOL52 | Sweroside aglycone | NQO2，CA2，GABRA1，PRSS1，F2 |
| MOL53 | isoorientin | CA2，PTPN1，NFE2L2，NQO1 |
| MOL54 | Benzo[a]carbazole | PTGS1，F2，ESR1，PTGS2，NOS3，ACHE，MAPK14，MAOB |
| MOL55 | lappadilactone | AR |
| MOL56 | Mairin | ESR1，AR |
| MOL57 | cynaropicrin | NOS2，F2，ESR1，AR，PTGS2 |
| MOL58 | Costunolide | CNR2，SMAD3，CHRNA4，NOS2，F2，NOS3，ACHE，GABRA1 |
| MOL59 | costuslactone | NOS2，F2，PTGS2，NOS3，ACHE，GABRA1，PRSS1 |
| MOL60 | dihydrocostus lactone | NOS2，F2，NOS3，ACHE，GABRA1 |
| MOL61 | Isoalantolactone | F2，ACHE，GABRA1 |
| MOL62 | isodihydrocostunolide | F2，ACHE，GABRA1 |
| MOL63 | beta-Ionone | CHRNA4，CYP2A6，ENPEP，NOS2，CYP17A1，RARA |
| MOL64 | poncirin | BDKRB1，F2R，KISS1R，HPSE，ODC1，ENPP2 |
| MOL65 | Isosinensetin | NOS2，PTGS1，F2，ESR1，AR，ABCC8，PPARG，PTGS2，NOS3，CA2，ACHE，PTPN1，ADRB2，ESR2，PRSS1 |
| MOL66 | 5,7,4'-Trimethylapigenin | NOS2，PTGS1，ESR1，AR，PPARG，PTGS2，NOS3，CA2，ADRA1B，PTPN1，ADRB2，ESR2，MAPK14，GSK3B，PRSS1，CCNA2 |
| MOL67 | 6-Methoxy aurapten | NOS2，CHRM3，F2，ESR1，AR，PPARG，PTGS2，NOS3，CA2，ADRA1B，ADRB2，ESR2，PRSS1 |
| MOL68 | neohesperidin_qt | NOS2，PTGS1，ESR1，AR，PPARG，PTGS2，CA2，PTPN1，ESR2，MAPK14，GSK3B，PRSS1，CCNA2 |
| MOL69 | Sinensetin | NOS2，F2，ESR1，AR，PPARG，PTGS2，CA2，ACHE，PTPN1，ADRB2，ESR2，PRSS1，CCNA2，PTGS1，ADRA1B |
| MOL70 | Eriodyctiol (flavanone) | NOS2，PTGS1，ESR1，AR，PPARG，PTGS2，CA2，PTPN1，ESR2，MAPK14，GSK3B，CCNA2，PRSS1 |
| MOL71 | naringenin | NOS2，PTGS1，ESR1，AR，PPARG，PTGS2，CA2，PTPN1，ESR2，MAPK14，GSK3B，CCNA2，RELA，AKT1，BCL2，MAPK3，MAPK1，CASP3，FASN，LDLR，SOD1，CAT，PPARG，MTTP，APOB，HMGCR，CYP19A1，GSTP1，PPARA，SREBF1，GSR，ABCC1，ADIPOQ，AKR1C1，GOT1，ABAT，SOAT1 |
| MOL72 | 5,7-dihydroxy-2-(3-hydroxy-4-methoxyphenyl)chroman-4-one | NOS2，PTGS1，ESR1，AR，PPARG，PTGS2，CA2，PTPN1，ESR2，MAPK14，GSK3B，PRSS1，CCNA2 |
| MOL73 | nobiletin | NOS2，PTGS1，F2，ESR1，AR，PPARG，PTGS2，CA2，PTPN1，ESR2，PRSS1，GSK3B，CCNA2，BCL2，BAX，MMP9，JUN，TP53，MAPK8，TIMP1，PPARG，PLA2G4A，CD163 |
| MOL74 | didymin | NOS2，PTGS1，ESR1，AR，PPARG，PTGS2，CA2，PTPN1，ADRB2，ESR2，MAPK14，GSK3B，PRSS1，CCNA2 |
| MOL75 | luteolin | NOS2，PTGS1，ESR1，AR，PPARG，PTGS2，CA2，PTPN1，ESR2，MAPK14，PRSS1，CCNA2，GSK3B，RELA，EGFR，AKT1，VEGFA，CCND1，BCL2L1，CDKN1A，MMP2，MMP9，MAPK1，IL10，RB1，CDK4，TNF，JUN，IL6，CASP3，TP53，NFKBIA，APP，MMP1，PCNA，ERBB2，PPARG，HMOX1，CASP7，ICAM1，MCL1，BIRC5，IL2，IFNG，IL4，GSTP1，XIAP，PTGES，MET，BCL2 |
| MOL76 | Tetramethoxyluteolin | NOS2，PTGS1，F2，ESR1，AR，PPARG，PTGS2，NOS3，CA2，ADRA1B，PTPN1，ADRB2，ESR2，MAPK14，GSK3B，PRSS1，CCNA2，ACHE，NR1I2 |
| MOL77 | 4-[(2S,3R)-5-[(E)-3-hydroxyprop-1-enyl]-7-methoxy-3-methylol-2,3-dihydrobenzofuran-2-yl]-2-methoxy-phenol | NOS2，F2，ESR1，AR，PPARG，PTGS2，CA2，PTPN1，ESR2，MAPK14，GSK3B，PRSS1，CCNA2，ACHE |
| MOL78 | Prangenin | NOS2，F2，ESR1，AR，PTGS2，MAPK14，GSK3B |
| MOL79 | poncimarin | NOS2，F2，ESR1，AR，PTGS2，GSK3B |
| MOL80 | isoponcimarin | NOS2，F2，ESR1，AR，PTGS2，ADRB2，MAPK14，GSK3B |
| MOL81 | citrusin B | F2 |
| MOL82 | Ammidin | NOS2，F2，ESR1，AR，PPARG，PTGS2，ESR2，GABRA1，MAPK14，CCNA2，GSK3B，MAOB |
| MOL83 | hesperidin | BAX，CASP3，PTGS2，ICAM1，VCAM1 |

**Supplementary Table S4. The correspondence between gastric cancer-related pathways and targets**

| **No.** | **Pathway** | **Degree** | **Target** |
| --- | --- | --- | --- |
| 1 | TNF signaling pathway | 17 | ICAM1,CSF2,IL6,TNF,PTGS2,RELA,MMP9,NFKBIA,AKT1,VCAM1,MAPK1,FOS,CASP3,CASP7,JUN,MAPK14,CASP8 |
| 2 | Apoptosis | 14 | AKT1, CASP3, TNF, XIAP, CASP7, BCL2, RELA, BAX, CASP8, CYCS, TP53, NFKBIA, FASLG, BCL2L1 |
| 3 | T cell receptor signaling pathway | 17 | IL4, IL2, PPP3CA, MAPK3, IFNG, MAPK14, GSK3B, JUN, FOS, MAPK1, AKT1, IL10, CDK4, NFKBIA, RELA, TNF, CSF2 |
| 4 | PI3K-Akt signaling pathway | 29 | MCL1, F2R, IL2, MAPK3, VEGFA, GSK3B, CCND2, CCND1, CDKN1A, MAPK1, KDR, CDK4, TP53, MET, RELA, FLT1, IL6, IL4, EGFR, PRKCA, MYC, FGF2, EGF, NOS3, TEK, BCL2, AKT1, BCL2L1, FASLG |
| 5 | NOD-like receptor signaling pathway | 11 | MAPK1, CASP1, MAPK8, IL1B, NFKBIA, CASP8, MAPK3, RELA, MAPK14, TNF, IL6 |
| 6 | Toll-like receptor signaling pathway | 14 | IL6, MAPK8, IL1B, MAPK3, CASP8, MAPK14, JUN, FOS, MAPK1, AKT1, STAT1, NFKBIA, RELA, TNF |
| 7 | MAPK signaling pathway | 19 | PRKCA, EGFR, TNF, RELA, TP53, FASLG, TGFB1, AKT1, FOS, MAPK1, CASP3, PLA2G4A, JUN, MAPK14, MAPK3, IL1B, MAPK8, PPP3CA, EGF |
| 8 | p53 signaling pathway | 11 | CDK1, CDKN1A, CASP3, CCND1, CCND2, BAX, CYCS, CASP8, TP53, CDK4, IGFBP3 |
| 9 | Fc epsilon RI signaling pathway | 10 | PRKCA, IL4, AKT1, CSF2, MAPK1, PLA2G4A, TNF, MAPK14, MAPK3, MAPK8 |
| 10 | B cell receptor signaling pathway | 10 | AKT1, MAPK1, FOS, INPPL1, RELA, JUN, GSK3B, MAPK3, NFKBIA, PPP3CA |
| 11 | Cell cycle | 13 | CDK1, TP53, SMAD3, RB1, CDK4, TGFB1, CDKN1A, CCND1, CCND2, GSK3B, PCNA, MYC, CCNA2 |
| 12 | NF-kappa B signaling pathway | 11 | VCAM1, ICAM1, TNF, XIAP, PTGS2, RELA, BCL2, NFKBIA, IL1B, BCL2L1, PLAU |
| 13 | Ras signaling pathway | 17 | PRKCA, EGFR, FLT1, RELA, MET, FASLG, BCL2L1, KDR, AKT1, MAPK1, PLA2G4A, TEK, VEGFA, MAPK3, MAPK8, EGF, FGF2 |
| 14 | Jak-STAT signaling pathway | 13 | IL4, AKT1, CSF2, IL6, CCND1, CCND2, IFNG, BCL2L1, STAT1, MYC, IL10, STAT3, IL2 |
| 15 | Drug metabolism - cytochrome P450 | 9 | GSTM1, CYP3A4, GSTM2, CYP2B6, MAOA, MAOB, CYP2A6, CYP1A2, GSTP1 |
| 16 | Epithelial cell signaling in Helicobacter pylori infection | 8 | EGFR, CASP3, MAPK14, RELA, JUN, MET, NFKBIA, MAPK8 |
| 17 | Inflammatory mediator regulation of TRP channels | 8 | PRKCA, PLA2G4A, MAPK14, IL1B, BDKRB1, MAPK8, PRKCE, PRKCD |
| 18 | TGF-beta signaling pathway | 7 | MAPK1, TNF, MAPK3, IFNG, SMAD3, MYC, TGFB1 |
| 19 | Intestinal immune network for IgA production | 5 | IL4, IL6, TGFB1, IL10, IL2 |
| 20 | Platelet activation | 8 | AKT1, MAPK1, PLA2G4A, MAPK14, MAPK3, PTGS1, NOS3, F2R |
| 21 | mTOR signaling pathway | 5 | PRKCA, AKT1, MAPK1, TNF, MAPK3 |
| 22 | Bile secretion | 5 | LDLR, HMGCR, SLC2A1, CA2, SLC10A2 |
| 23 | Arginine and proline metabolism | 7 | ODC1, GOT1, NOS1, MAOA, MAOB, NOS3, NOS2 |
